# Combinatorial selective ER-phagy remodels the ER during neurogenesis

**DOI:** 10.1038/s41556-024-01356-4

**Published:** 2024-03-01

**Authors:** Melissa J. Hoyer, Cristina Capitanio, Ian R. Smith, Julia C. Paoli, Anna Bieber, Yizhi Jiang, Joao A. Paulo, Miguel A. Gonzalez-Lozano, Wolfgang Baumeister, Florian Wilfling, Brenda A. Schulman, J. Wade Harper

**Affiliations:** 1 Department of Cell Biology, Harvard Medical School, Boston, MA USA; 2 Aligning Science Across Parkinson’s (ASAP) Collaborative Research Network, Chevy Chase, MD USA; 3 Department of Molecular Machines and Signaling, Max Planck Institute of Biochemistry, Martinsried, Germany; 4 Department of Molecular Structural Biology, Max Planck Institute of Biochemistry, Martinsried, Germany; 5 Mechanisms of Cellular Quality Control, Max Planck Institute of Biophysics, Frankfurt am Main, Germany; 6 Present Address: Velia Therapeutics, San Diego, CA USA

**Keywords:** Endoplasmic reticulum, Macroautophagy

## Abstract

The endoplasmic reticulum (ER) employs a diverse proteome landscape to orchestrate many cellular functions, ranging from protein and lipid synthesis to calcium ion flux and inter-organelle communication. A case in point concerns the process of neurogenesis, where a refined tubular ER network is assembled via ER shaping proteins into the newly formed neuronal projections to create highly polarized dendrites and axons. Previous studies have suggested a role for autophagy in ER remodelling, as autophagy-deficient neurons in vivo display axonal ER accumulation within synaptic boutons, and the membrane-embedded ER-phagy receptor FAM134B has been genetically linked with human sensory and autonomic neuropathy. However, our understanding of the mechanisms underlying selective removal of the ER and the role of individual ER-phagy receptors is limited. Here we combine a genetically tractable induced neuron (iNeuron) system for monitoring ER remodelling during in vitro differentiation with proteomic and computational tools to create a quantitative landscape of ER proteome remodelling via selective autophagy. Through analysis of single and combinatorial ER-phagy receptor mutants, we delineate the extent to which each receptor contributes to both the magnitude and selectivity of ER protein clearance. We define specific subsets of ER membrane or lumenal proteins as preferred clients for distinct receptors. Using spatial sensors and flux reporters, we demonstrate receptor-specific autophagic capture of ER in axons, and directly visualize tubular ER membranes within autophagosomes in neuronal projections by cryo-electron tomography. This molecular inventory of ER proteome remodelling and versatile genetic toolkit provide a quantitative framework for understanding the contributions of individual ER-phagy receptors for reshaping ER during cell state transitions.

## Abstract (web summary)

Hoyer et al. establish that selective autophagy mechanisms are needed to remodel the ER and its proteome during in vitro neurogenesis across neuronal subcompartments and decode the substrate selectivity of ER-phagy receptors.

## Main

The endoplasmic reticulum (ER) network is shaped by proteins that promote tubule and sheet-like membrane structures, which in turn tailors ER function in a cell-type-specific manner to optimize protein secretion, calcium storage, lipid homeostasis and inter-organelle contacts^1–4^. ER-phagy is a mechanism through which ER can be remodelled, or superfluous ER proteins or lipids recycled^5–7^. Membrane-bound ER-phagy receptors include single-pass transmembrane (TM) segment containing proteins TEX264, CCPG1, SEC62 and reticulon-like hairpin domain (RHD) containing proteins FAM134A, B and C (also called RETREG2, 1, 3, respectively), Atlastin (ATL2) and RTN3L^7–15^. RHDs reside in the outer leaflet of the ER membrane to induce curvature^16–18^. All ER-phagy receptors contain cytosolic LC3-interaction region (LIR) motifs that bind to ATG8 proteins such as MAP1LC3B (also called LC3B) on the phagophore to promote ER capture^5^. The FAM134 class of receptors are thought to cluster through their hairpin RHDs into highly curved nanoscale membrane domains that recruit the autophagy machinery, thereby nucleating phagophore formation^5,6,19–21^. Phagophore closure around the ER is thought to be coupled to scission of the ER membrane, although the mechanism is unknown.

Central unanswered questions in the field include when, where and how individual receptors are used to remodel the ER during physiological changes in the cell state. In addition, the identity of ER-phagy ‘cargos’ in unique cell states is poorly understood. Although ER protein accumulation has been observed in ATG5^−/−^ mouse synaptic boutons^22^, this was attributed to non-selective autophagy rather than selective ER-phagy^22^. An understanding of ER-phagy is further confounded by ER membranes both serving as a source of phospholipids for autophagosome expansion^23^ and as being captured as cargo within a fully formed autophagosome via selective ER-phagy, as visualized by electron microscopy^9,10^. However, critical work has revealed that the process of lipid transfer from the ER to the growing autophagosome occurs without the incorporation of ER proteins into the phagophore membrane itself^23,24^. Thus, the process of ER-phagy receptor-facilitated ER protein clearance is functionally and mechanistically distinct from the use of ER membranes as a source for phospholipids in phagophore expansion.

In this Article we employ an in vitro neurogenesis system that recapitulates central autophagy-dependent features of ER remodelling^25^ to directly examine the role of ER-phagy receptors in this process. We identify redundant and selective ER-phagic cargo for individual receptors, demonstrate a role for multiple ER-phagy receptors in eliminating axonal ER, directly visualize ER-phagy receptors trafficking in autophagosomes in axons, and visualize tubular ER membranes captured within autophagosomes in neuronal projections via cryo-electron tomography (cryo-ET). We find that ER protein remodelling by autophagy during neurogenesis facilitates a continuum of small abundance changes in individual ER-resident proteins. We implement a quantitative proteomic framework capable of measuring and classifying these abundance changes across the ER proteome in the context of an allelic series of ER-phagy receptor mutants. We find that FAM134 family members play a dominant and largely redundant role in remodelling ER membrane proteins during neurogenesis, whereas CCPG1 is primarily responsible for autophagic turnover of lumenal ER proteins, thereby defining an underlying specificity for ER remodelling. These data provide a proteomic landscape for ER remodelling in induced neurons (iNeurons) and an experimental framework for elucidating how changes in cell state control the ER proteome via selective autophagy.

## Results

### ER remodelling by autophagy during in vitro neurogenesis

**Fig. 1:**
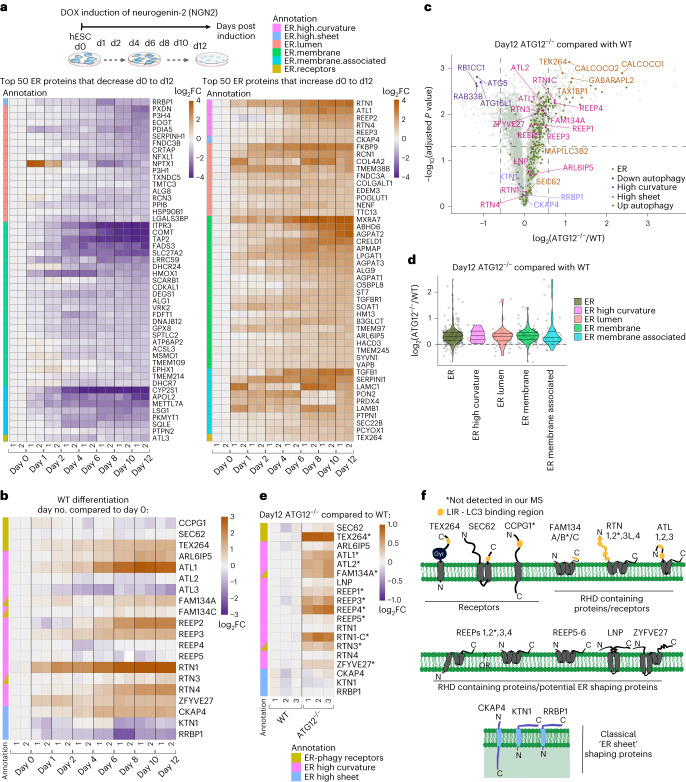
Landscape of ER remodelling via autophagy during hESC differentiation to iNeurons in vitro. **a**, Changes in abundance of the most highly remodelled ER proteins during conversion of WT hESCs to iNeurons are shown in heatmaps (log_2_ fold change (FC) at the indicated day of differentiation relative to hESCs). The top 50 proteins that either decrease or increase in abundance are shown (see Extended Data Fig. 1b for a full heatmap). Data are from our previous analysis of iNeuron differentiation. Annotations depicting the type of ER protein are indicated by the relevant colours. **b**, Heatmap (log_2_FC) of ER-shaping proteins specifically in differentiating iNeurons. **c**, Volcano plot (−log_10_(adjusted *P* value) versus log_2_FC (ATG12^−/−^/WT)) of day-12 WT and ATG12^−/−^ iNeuron total proteomes, displaying accumulation of autophagy-related and ER proteins (green dots) as a cohort. Each dot represents the average of triplicate TMT measurements. *P* values were calculated from the Student’s *t*-test (two sided) and adjusted for multiple hypothesis correction using the Benjamini–Hochberg approach. **d**, Violin plots for individual classes of ER proteins showing the relative increases in abundance in ATG12^−/−^ day-12 iNeurons compared with WT iNeurons. Each dot represents the average of triplicate TMT measurements. **e**, Heatmap (log_2_FC) of ER-shaping proteins specifically in day-12 WT versus ATG12^−/−^ iNeurons. An asterisk after a gene name indicates significant changes in abundance: *Adjusted *P* < 0.05, Student’s *t*-test (two-sided), multiple hypothesis correction using the Benjamini–Hochberg approach. **f**, Topology of ER-shaping proteins and ER-phagy receptors within the ER membrane. The annotation colour scheme for individual classes of ER proteins in **e** also applies to **b**. MS, mass spectrometry.

**Extended Data Fig. 1:**
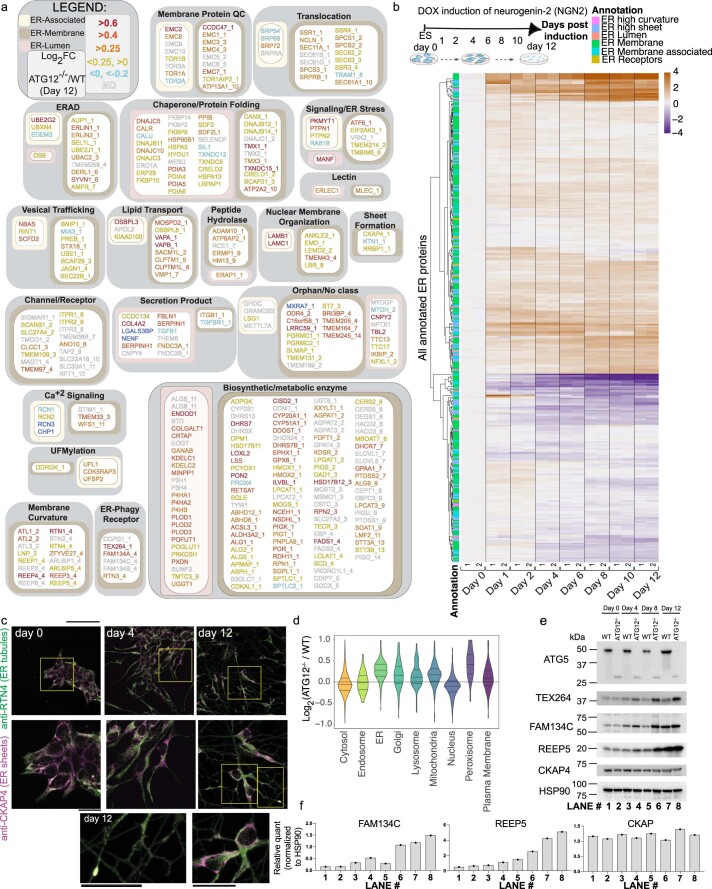
Landscape of ER remodelling via autophagy during hESC differentiation to iNeurons in vitro. **a**, Landscape of the ER proteome and the effect of autophagy on accumulation of individual proteins. The ER proteome (359 proteins, Supplementary Data Table 1) is organized into functional modules and protein attributes (involved in ER membrane curvature, ER-associated, ER-membrane, ER-Lumen or ER-phagy receptor) are indicated by the respective outline box colour (see inset legend). For proteins with transmembrane segments, the number of segments are indicated after the protein name (_1, _2, etc) based on data in Uniprot. The text of each protein name is coloured based on day12 ATG12^−/−^ vs WT Log_2_FC (see inset legend). (Supplementary Data Table 3). **b**, Changes in the abundance of the ER proteome (267 detected proteins) during conversion of WT hESCs to iNeurons are shown in as heatmaps (Log_2_FC) at the indicated day of differentiation relative to hESCs. Data are from our previous analysis of iNeuron differentiation. Annotations of the type of ER protein are indicated by the relevant colours. **c**, hESCs were differentiated to iNeurons and stained with antibodies against CKAP4 enriched in ER sheets (magenta) and RTN4 enriched in ER-tubules (green) at day 0, 4 and 12 of one differentiation. RTN4 staining is evident throughout neuronal projections. Scale bar, 100 microns in full images, 25 microns in all zooms. **d**, Violin plots for relative abundance of proteins located in the indicated organelles in ATG12^−/−^ versus WT day 12 iNeurons. **e**, Immunoblots of cell extracts from WT or ATG12 ^−/−^ hESCs for the indicated day of differentiation for one differentiation. Blots were probed with the indicated antibodies, with α-HSP90 employed as a loading control. **f**, For the immunoblots in **e**, relative levels of each protein to HSP90 were quantified. Source numerical data and unprocessed blots are available in source data. Source data

To examine the alterations in abundance for approximately 350 ER proteins^26^ (Extended Data Fig. 1a and Supplementary Table 1), we initially mined proteome abundance measurements from our previous human embryonic stem cell (hESC) neurogenesis resource^25^ (Fig. 1a). During a 12-day iNeuron differentiation, a diverse cohort of ER proteins within multiple functional categories increase or decrease in abundance (Fig. 1a,b, Extended Data Fig. 1b and Supplementary Table 2)^25^. Proteins undergoing the largest increase in abundance include enzymes involved in protein folding (for example, FKBP9), ion regulation (for example, RCN1) and collagen modification (for example, COL4A2), whereas other collagen-modifying proteins (PXDN and P3H4) decrease in abundance (Fig. 1a). The ER-shaping RHD proteins RTN1, RTN4 and REEP2 displayed the largest increase in abundance, with a greater than 1.4-fold increases from day-0 levels (that is, a log_2_(fold change day 12 versus day 0) greater than 0.5, more simply shown as log_2_FC > 0.5; Fig. 1b and Extended Data Fig. 1b). This is consistent with the formation of ER tubule networks within neuronal projections, as previously characterized^27^. Indeed, immunofluorescence revealed extensive α-RTN4-positive projections in iNeurons, while the ER sheet protein CKAP4 (also called CLIMP63) was largely confined to the soma (Extended Data Fig. 1c).

**Extended Data Fig. 2:**
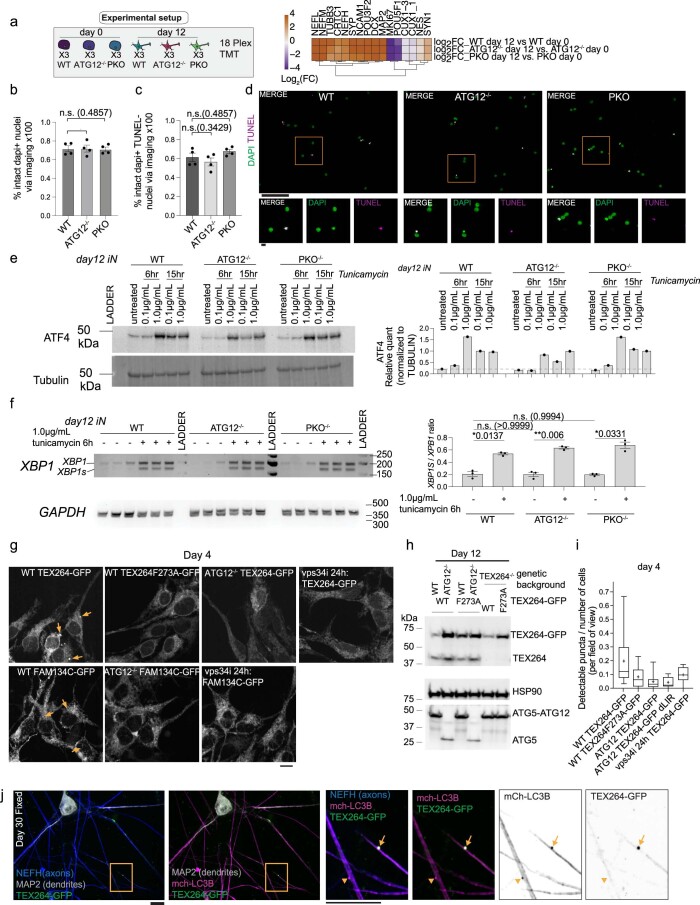
Quality control of in vitro neurogenesis methods. **a**, The indicated hESCs were either cultured in the pluripotent state (day 0) or converted to day 12 iNeurons prior to total proteome analysis by multiplex TMT in biological triplicate cultures (n=3). The relative abundance (Log_2_ FC) of the indicated neurogenesis or pluripotency factors at day 12 relative to day 0 is shown, indicating that all genotypes undergo differentiation to a similar extent as measured by markers of the process. **b**, Comparable number of viable iNeurons were observed upon quantitative analysis of intact DAPI-positive nuclei compared to all DAPI-positive (intact and fragmented) DNA structures in cultures of the indicated genotypes. Four points shown for each WT or KO condition represent the measured ratios from the same four independent differentiations also analysed for ER structure per nuclei in Fig. 2b. Data are presented as mean values +/-SEM. *, p<0.05, two sided Mann–Whitney test. **c,d** Quantification of DAPI -positive TUNEL-negative nuclei in day 20 iNeurons from the indicated genotypes (**c**) and representative images of DAPI-positive nuclei in green and TUNEL-stained DAPI-positive fragmented nuclei, with TUNEL signal in magenta (**d**). The lower panels are magnified regions (merges and separate image channels) of the area boxed in the respective image above. Four points shown for each WT or KO condition represent the measured values from four independent differentiations. Data are presented as mean values +/-SEM. *, p<0.05, two sided Mann–Whitney test. Scale bar, 100 microns (top), 10 microns (bottom) **e**, ATF4 abundance was examined by immunoblotting of extracts from day 12 iNeurons from the indicated genotypes with or without treatment with tunicamycin as a positive control for induction of the ER-stress stress response. Tunicamycin was used at 0.1 or 1.0 μg/ml for 6 or 12 h, as indicated. Blots were reprobed with α-tubulin as a loading control. The relative α-ATF4 signal, normalized for tubulin, is shown in the histogram (right panel) with the dashed line representing ATF4 signal in untreated WT cells. n=1. **f**, *XBP1* and *XBP1s* mRNA from the indicated iNeurons in biological triplicate (n=3) cultures was subjected to reverse transcription-PCR (see Methods) and examined by agarose gel electrophoresis to resolve spliced and unspliced *XBP1*. 1.0 μg/ml tunicamycin (6h) was employed as a positive control for induction of ER stress and *XBP1* splicing. *GAPDH* was used as a positive control. The *XBP1s*/*XBP1* ratio was quantified as shown in the histogram (right panel). No evidence of increased *XBP1* splicing was observed in any genotype under untreated conditions. Data are presented as mean values +/-SEM. *, p<0.05; **, p<0.01; n.s., not significant; Brown–Forsythe and Welch One-way ANOVA and Dunnett’s T3 multiple comparisons test. **g**, TEX264-GFP, TEX264^F273A^-GFP, or FAM134C-GFP were expressed in WT or ATG12^−/−^ hESCs and cells imaged at day 4 of differentiation to iNeurons. In some experiments, VPS34i was added to WT cells for 24h prior to imaging. Arrows mark examples of ER-phagy receptor puncta. **h**, Expression of TEX264-GFP was verified by immunoblotting of iNeuron extracts using α-HSP90 as a loading control. **i**, Number of TEX264-GFP puncta was quantified in day 4 iNeurons (n = 1 independent differentiation). Min-to-max box-and-whiskers representing detectable number of puncta per cell, with box representing the 25^th^ to 75^th^ percentile, whiskers going from min to max values, line at median and + at mean. **j**, Day 30 iNeurons expressing TEX264-GFP and mCh-LC3B were immunostained with α-MAP2 to detect dendrites (white) and α-NEFH to mark axons (blue) and projections imaged by confocal microscopy. One differentiation was fixed and imaged this way. Insets show TEX264-GFP/mCh-LC3B-positive puncta (arrows) or mCh-LC3B-positive but TEX264-GFP-negative puncta (arrowheads) in axons. All scale bars, 10 microns. Source numerical data and unprocessed blots are available in source data. Source data

We next compared wildtype (WT) and ATG12^−/−^ day-12 iNeurons using tandem mass tagging (TMT) proteomics (Fig. 1c and Supplementary Table 3). ATG12 is conjugated to ATG5 to support lipidation of ATG8 proteins. Consistent with reduced autophagic clearance^25^, ubiquitin-binding autophagy receptors (CALCOCO1, CALCOCO2, TAX1BP1) and the ATG8 protein GABARAPL2 accumulated in ATG12-deficient iNeurons, as did the ER-phagy receptors TEX264 and FAM134A (>1.4-fold change, log_2_FC > 0.49; Fig. 1c). Moreover, a cohort of ER proteins displayed increased abundance, as indicated by the rightward skew distribution in volcano plots of log_2_FC values for ATG12^−/−^/WT proteomes. Similarly, violin plots revealed an overall increase in ER protein abundance, which showed a mean log_2_FC of 0.33 (1.26-fold increase across all ER proteins; Fig. 1c,d and Extended Data Fig. 1d). Strikingly, RHD proteins accumulate to the greatest degree (including REEP1–4 and RTN1), whereas the ER sheet proteins CKAP4 and RRBP1 were unchanged (Fig. 1d–f). Alterations in abundance for TEX264, REEP5 and CKAP4 were verified by immunoblotting, as was the increased abundance of FAM134C (not detected by proteomics in this experiment; Extended Data Fig. 1e,f). Mapping the landscape of ER protein accumulation in ATG12 deletion iNeurons (log_2_FC from WT) revealed that, beyond ER curvature-shaping proteins, specific ER proteins assigned to several other structural or functional categories accumulate during differentiation in the absence of autophagy, including lumenal and TM segment-containing biosynthetic or metabolic proteins (Extended Data Fig. 1a). The differentiation efficiency of ATG12^−/−^ iNeurons was equivalent to WT iNeurons as assessed by the induction or loss of several differentiation/pluripotency factors (Extended Data Fig. 2a and Supplementary Table 4). Moreover, ATG12^−/−^ iNeuron viability was equivalent to WT iNeurons (Extended Data Fig. 2b–d). We also examined whether ATG12 deficiency promotes ER stress, but detected no increase in the ER stress response markers ATF4 (protein level expression) or *XBP-1* (mRNA splicing) when compared with WT iNeurons, although tunicamycin treatment induced both ATF4 expression and *XBP-1* splicing (Extended Data Fig. 2e,f). Thus, in vitro neurogenesis without autophagy is associated with alterations in the abundance of the ER proteome.

### Aberrant axonal ER accumulation in ATG12^−/−^ neurogenesis

**Fig. 2:**
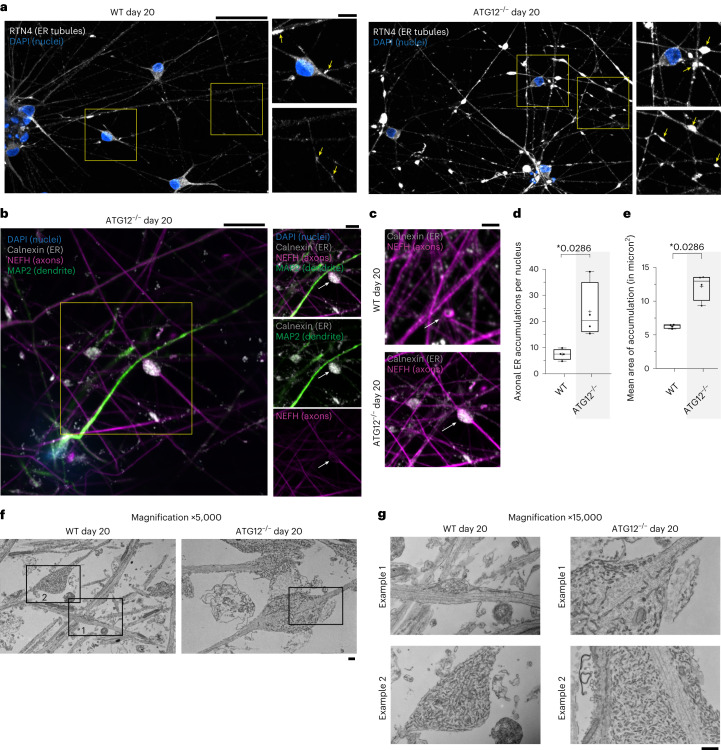
Autophagy-dependent clearance of ER in axons during iNeuron differentiation. **a**, WT or ATG12^−/−^ day-20 iNeurons immunostained with ER-tubule marker α-RTN4 (white) and with DAPI (nuclei, blue). Scale bars, 50 μm (full images) and 10 μm (zooms). **b**, Enlarged ER-positive structures in ATG12^−/−^ day-20 iNeuron axons revealed by immunostaining with α-calnexin, ER (white); α-MAP2, dendrites (green); α-NEFH, axons (magenta); and DAPI, nuclei (blue). Scale bars, 10 μm (full image) and 5 μm (zooms). **c**, As in **b**, day-20 iNeurons were immunostained with α-NEFH and α-calnexin to identify aberrant ER structures; here we compare the zoomed-in region of axons in ATG12^−/−^ iNeurons to a similar region for WT iNeurons. Scale bar, 5 μm. **d**, Min-to-max box-and-whiskers plot for the number of axonal ER accumulations per nucleus, where the box represents the 25th to 75th percentiles, whiskers extend from min to max values, the line represents the median and + the mean. Points represent mean values from four independent differentiations (*n* = 4). *P < 0.05, two-sided Mann–Whitney test. **e**, Min-to-max box-and-whiskers plot for the area of ER accumulations in axons, where the box represents the 25th to 75th percentiles, whiskers extend from min to max values, the line represents the median and + the mean. Four points for each condition give the resulting mean areas from four independent differentiations. *P < 0.05; two-sided Mann–Whitney test. **f**,**g**, Scanning transmission EM of thin sections from WT and ATG12^−/−^ iNeuron cultures (day 20, one differentiation). Panel **f** presents low-magnification images through multiple axons. Panel **g** presents high-magnification images of WT example 1 and example 2 and one ATG12 region, all outlined in **f**, as well as one additional zoom example 2 from another ATG12^−/−^ iNeuron field of view. Scale bars, 500 nm.

We next examined ER morphology in WT or ATG12^−/−^ day-20 iNeurons using α-calnexin or α-RTN4 as general or tubule-enriched markers for ER, respectively. We observed ER-positive accumulations that dilated the projections in autophagy-deficient cells that were larger and more numerous than seen in WT iNeurons (Fig. 2a–e). α-NEFH (high-molecular-weight neurofilament-H) staining verified that the dilations were present within axons, with NEFH filaments encasing ER dilations (Fig. 2b, inset). The mean area of ER accumulations dilating the axons in ATG12^−/−^ iNeurons was 12.2 μm^2^, whereas in WT iNeurons these were less abundant and consistently smaller (mean area, 6.3 μm^2^) (Fig. 2c–e). Consistent with light microscopy, scanning transmission electron microscopy (TEM) revealed frequent dilated ER-rich bulbous structures in ATG12^−/−^ iNeurons adjacent to continuous microtubules that were rare and smaller in WT processes (Fig. 2f,g). These ER-rich dilations are reminiscent of the previously observed axon boutons within mouse neurons lacking *Atg5*^22^.

### ER-phagic flux during differentiation and in iNeurons

**Fig. 3:**
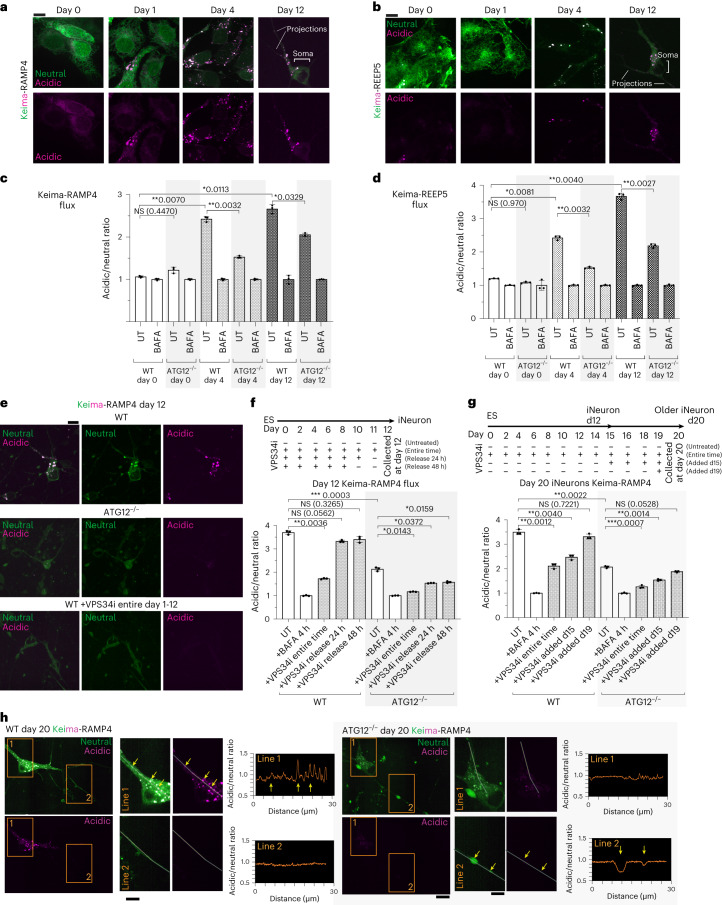
ER-phagic flux in iNeurons. **a**, hESCs expressing Keima-RAMP4 were differentiated to iNeurons. Keima was imaged at days 0, 1, 4 and 12. Scale bar, 10 μm. **b**, hESCs expressing Keima-REEP5 were differentiated to iNeurons and the Keima signal was imaged at days 0, 1, 4 and 12. Representative cell images are from one differentiation experiment. Scale bar, 10 μm. **c**,**d**, WT or ATG12^−/−^ Keima-RAMP4 flux (**c**) or Keima-REEP5 flux (**d**) was measured by flow cytometry at days 0, 4 and 12 of differentiation. The ratio of acidic to neutral Keima fluorescence was normalized to samples treated with BAFA (100 nM, 4 h). **e**, Images of reduced Keima-RAMP4 flux in ATG12^−/−^ iNeurons or upon VPS34 inhibitor (VPS34i, 1 μM) treatment. Scale bar, 10 μm. **f**, WT or ATG12^−/−^ hESCs differentiated with or without VPS34i as indicated in the scheme. In some conditions, VPS34i was washed out at the time indicated (24 or 48 h), before collection at day 12 and subsequent analysis by flow cytometry. In **f** and **g**, the ratio of acidic to neutral Keima fluorescence was measured via flow cytometry as in **c**. **g**, Ongoing ER-phagic flux in day 15 iNeurons was measured. WT or ATG12^−/−^ hESCs were differentiated in the presence or absence of VPS34i, as indicated in the scheme. In some cases, VPS34i was added at day 19 or day 15, before collection at day 20 and subsequent analysis by flow cytometry. In **c**, **d**, **f** and **g**, each point represents one of three biological triplicate measurements (*n* = 3). Data are presented as mean values ± s.d. *P < 0.05; **P < 0.01; ***P < 0.001; NS, not significant; Brown–Forsythe and Welch one-way analysis of variance (ANOVA) and Dunnett’s T3 multiple comparisons test. **h**, Live cells expressing Keima-RAMP4 in WT and ATG12^−/−^ day-20 iNeurons were imaged. Representative cell images are from three replicate differentiation experiments. Scale bars, 10 μm (full images) and 5 μm (zooms). Insets: the results of acidic/neutral ratiometric line-scan analysis for somata (lines labelled 1) or axons (lines labelled 2) of WT or ATG12^−/−^ iNeurons.

We next measured the extent to which certain ER proteins were cleared from the ER membrane to acidic lysosomes via autophagy, which we define here as ER-phagic flux. We measured ER-phagic flux at different stages of differentiation and in post-differentiated ‘established’ iNeurons using a pH-sensitive Keima reporter localized throughout the ER (Keima-RAMP4, a pan-ER reporter widely used in the ER-phagy field; Fig. 3a) or a reporter localized selectively in ER tubules (Keima-REEP5; Fig. 3b). More specifically, our ER-phagy flux readout was derived from a ratiometric comparision of acidified Keima-ER within lysosomes with non-acidified Keima-ER throughout the ER network^9,13,28^. Neither reporter underwent significant flux in hESCs, consistent with the absence of ER protein accumulation in ATG12^−/−^ hESCs^25^. However, during differentiation, we observed an increase in acidic Keima signal (increased acidic/neutral ratio, as defined in the Methods) for both ER reporters, with acidified puncta representing ER in lysosomes located primarily in the soma (Fig. 3a,b). Parallel flow cytometry experiments quantified the amount of ER flux to lysosomes upon differentiation using both reporters (Fig. 3c,d). Acidic signal was normalized to cells treated with bafilomycin A (BAFA, 4 h), which inhibits lysosomal acidification. This ER flux was reduced in cells lacking ATG12, and residual flux was eliminated by addition, throughout the differentiation time course, of the VPS34 PI3 kinase inhibitor SAR405 (VPS34i), which blocks phagophore initiation (Fig. 3e,f). The detectable flux in ATG12^−/−^ cells is consistent with the previous finding that loss of the ATG8 conjugation system does not fully block autophagosome formation^29^. Due to the long half-life of Keima in lysosomes^30^, detectable stable Keima within lysosomes over multiple days of differentiation was expected. Release from continuous VPS34 inhibition one or two days before (at day 10 or 11) iNeuron collection (at day 12) resulted in increased ER-phagic flux comparable to that of untreated cells; this increase was absent in cells lacking ATG12 (Fig. 3f). Finally, we examined whether ER-phagic flux was ongoing in established iNeurons. Keima flux measured in later-stage day-20 neurons was reduced by adding VPS34i at day 15 of differentiation, as compared with untreated cells (Fig. 3g). These results indicate that ER fluxes to lysosomes during differentiation in a process that requires canonical autophagy, and that autophagic ER flux is ongoing in established iNeurons.

### Receptor capture by autophagosomes in axons and somata

It is well known that autophagosomes can form in axons, and subsequently fuse with lysosomes, which acidify during retrograde trafficking en route to the soma^31–33^. Thus, ATG12-dependent acidic Keima-RAMP4-positive puncta in the soma (Fig. 3a,b,h) could reflect ER-phagy occurring locally in the soma or, alternatively, axonal ER-phagic capture within autophagosomes followed by retrograde transport to the soma with concomitant acidification.

To examine the spatial aspects of ER-receptor capture, we expressed TEX264-GFP or FAM134C-GFP in iNeurons (Extended Data Fig. 2g,h). Previous studies have demonstrated that TEX264 and FAM134 proteins can localize broadly throughout the ER network and can form coincident puncta that become engulfed by autophagosomes^7,9^. We observed TEX264-GFP punctate structures (indicated by arrowheads) both in projections and in the soma (day 4 of differentiation) that were rarely detected: (1) when TEX264’s LIR motif was mutated (F273A), (2) in ATG12^−/−^ cells or (3) in VPS34i-treated cells (Extended Data Fig. 2g–i). Thus, ER-phagy receptor puncta formation in iNeurons was probably due to active ER-phagy, as described in other cell systems with starvation as a trigger for ER-phagy^7,9^.

**Fig. 4:**
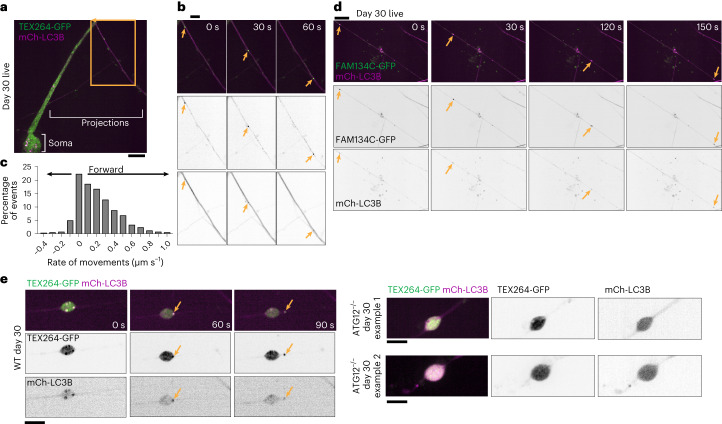
Axonal trafficking of TEX264-GFP and FAM134C-GFP-containing autophagosomes via live-cell imaging. **a**,**b**, TEX264-GFP (green) and mCh-LC3B (magenta) day-30 iNeurons imaged live (**a**), in a representative event from three replicate differentiation experiments. Inset in **b**: positions of mCh-LC3B/TEX264-GFP-positive puncta trafficking within an axon. Arrows indicate puncta positions over two indicated time sequences. Scale bars, 10 μm (**a**) and 5 μm (**b**). **c**, Rate of TEX264-GFP/mCh-LC3B-positive puncta movements (*n* = 429), and the percentage of events at the indicated speeds are binned in a histogram (events from three replicate differentiation experiments). **d**, As in **b**, but for FAM134C-GFP/mCh-LC3B-positive puncta, in a representative event from three replicate differentiation experiments. **e**, TEX264-GFP/mCh-LC3B-positive puncta are in dilated regions of WT iNeuron axons and traffic away (left), but puncta are not detected in ATG12^−/−^ iNeurons (right). Representative events from two replicate differentiation experiments are shown. Scale bars, 10 μm.

To verify TEX264-GFP puncta in autophagic structures, we co-expressed mCherry-LC3B (mCh-LC3B). Co-staining with α-NEFH in fixed cells verified the coincidence of mCh-LC3B and TEX264-GFP in axons (Extended Data Fig. 2j). We took advantage of the highly polarized axons in day-30 iNeurons to track the movement of mCh-LC3B/TEX264-GFP-positive puncta. Numerous GFP-TEX264 puncta trafficked with mCh-LC3-positive structures (Fig. 4a,b and Supplementary Video 1). Autophagosomes enriched in TEX264 moved unidirectionally (predominant movement in one direction on the track is defined here as ‘forward’), but we also recorded stops and some backward movements on these tracks (Fig. 4c). The median forward speed was 0.297 μm s^−1^ (Fig. 4c), similar to the speeds reported for autophagosomes undergoing microtubule-dependent trafficking in axons of mouse primary neurons^34^. Similarly, FAM134C-GFP-positive structures trafficking with mCh-LC3B puncta were also observed in day-30 iNeurons (Fig. 4d and Supplementary Video 2), indicating that multiple ER-phagy receptors may be operating within projections.

We next examined whether ER-rich axonal dilations might be sites of ER-phagic capture. Indeed, live-cell imaging revealed TEX264-GFP/mCh-LC3B-positive puncta emerging from axonal dilations in WT iNeurons (Fig. 4e and Supplementary Video 3). In contrast, although TEX264-GFP was present in regions with dilated axonal ER in ATG12^−/−^ iNeurons, TEX264-GFP/mCh-LC3B-positive puncta were not observed (Fig. 4e). These data suggest a role for ER-phagy receptor-dependent clearance of ER in axonal processes.

### ER within autophagosomes in neuronal projections by cryo-ET

**Fig. 5:**
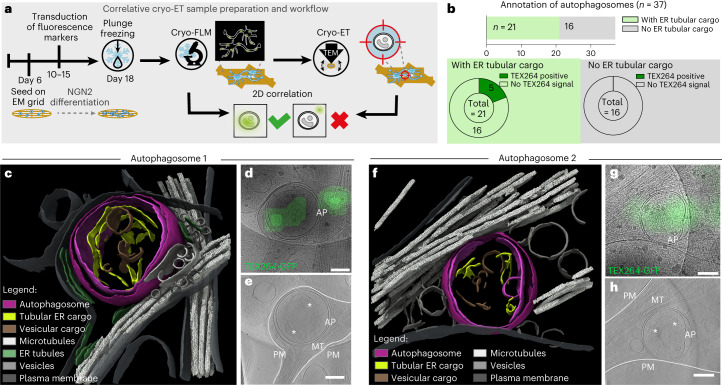
Observation of tubular ER within autophagosomes in neuronal projections by correlative cryo-ET. **a**, Experimental strategy used to capture autophagosomes in the projections of iNeurons. Induced pluripotent stem cells (iPSCs) were differentiated on EM grids and transduced with fluorescence markers before plunge-freezing at day 18. After imaging the sample by cryo-fluorescence microscopy (cryo-FLM), autophagosomes in the neuronal projections are identified in TEM images based on morphological features, and captured by cryo-ET. Two-dimensional (2D) correlation of TEM images with previously acquired fluorescence data shows whether the autophagosomes correlate or not with fluorescence markers such as TEX264-GFP. **b**, Cargo and TEX264-GFP correlation analysis of the captured autophagosomes. The barplot on top shows the number of autophagosomes in which ER tubular cargo is present (green, *n* = 21) or not (grey, *n* = 16). The pie charts show the number of structures corresponding to TEX264-GFP signal in each category. **c**–**h**, Examples of TEX264-GFP-positive autophagosomes with tubular ER cargo captured in situ by cryo-ET from one differentiation experiment. **c**,**f**, 3D segmentations reveal double-membrane autophagosomes (magenta) containing ER tubules as cargo (yellow) and close to microtubules (white). The tubular ER cargo of autophagosome 1 (**c**) exhibits a morphology similar to the adjacent cytosolic ER (green). For a full tomogram movie of autophagosome 1, see Supplementary Video 4. For full segmentation of the ER tubules, see Extended Data Fig. 3e. **d**,**g**, Zoomed-in 11500X TEM images corresponding to autophagosomes 1 (**d**) and 2 (**g**) overlaid with the TEX264-GFP cryo-fluorescence signal. For a complete view of fluorescence overlays, see Extended Data Fig. 3k,m. **e**,**h**, Tomogram slices of autophagosomes 1 (**e**) and 2 (**h**), denoised with cryo-CARE. White lines indicate the plasma membrane (PM) of the neuronal projections containing the autophagosomes. Asterisks indicate the tubular ER cargo visible in these slices. AP, autophagosome; MT, microtubule. All scale bars, 200 nm.

**Extended Data Fig. 3:**
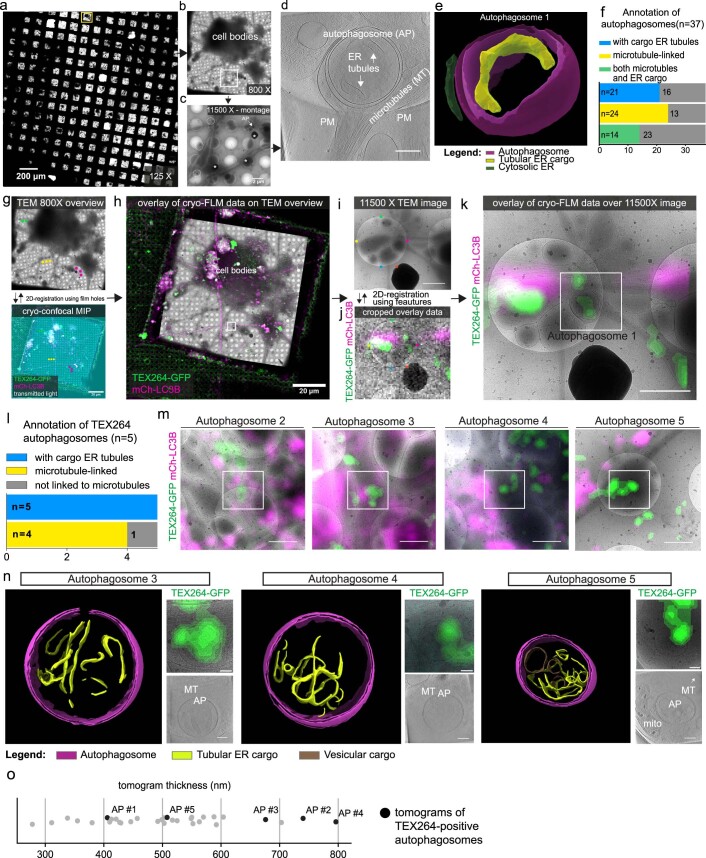
Analysis of autophagosomes in iNeurons by correlative cryo-ET. **a**,125X TEM image of the grid used in this study from one differentiation experiment. The yellow square indicates the region shown in b. **b**, 800X TEM overview of a grid square. The darker area corresponds to the thicker iNeuron cell bodies; around it, many thinner projections corresponding to axon and dendrites can be observed. The white square indicates a particular area rich in neuronal projections, shown in c. **c**, 11500X TEM montage acquired to map a potentially interesting area. Extracellular vesicles are also present (white dashed circles). Pieces of ice are sitting on top of the frozen sample (asterisks). Autophagosome 1 (AP) was found in this area. **d**, Slice of the tomogram of autophagosome 1, denoised with Cryo-CARE. Scale bar, 200 nm. **e**, 3D rendering of autophagosome 1 with manually refined segmentations of a cytosolic ER tubule (dark green) and a portion of the tubular ER cargo (yellow). **f**, Cumulative barplot showing number of autophagosomes containing tubular ER cargo (n=21), linked to microtubules (n=24) or both (n=14). **g**, Example of the 2D-correlation workflow for autophagosome 1. Correlation of the 800X TEM overview with cryo-confocal data, represented by a single in-focus slice of the transmitted light stack (cyan) and maximum intensity projection (MIP) of fluorescence channels. The coloured dots indicate the position of the 2 µm holes that were selected in both images for 2D-registration. After 2D registration, the TEM overview is transformed to match the fluorescence data. **h**, Overlay of the fluorescence data with the transformed TEM overview. The white square indicates the cropped area used for the second step of the correlation procedure. **i**, 11500X TEM image of the area around autophagosome 1. The coloured dots indicate features of the image that were used for finer correlation with the cropped fluorescence data. **j**, Cropped fluorescence data corresponding to the square in g. The colored dots indicate the same correlation points shown in h. The fluorescence data is subsequently transformed to match the 11500X TEM image. **k**, Final overlay of the fluorescence data over the 11500X TEM image. The white square represents the tomogram position. **l**, Cumulative barplot showing number of TEX264-GFP positive autophagosomes that contain ER tubular cargo (n=5) and are linked to microtubules (n=4). **m**, 11500X TEM and fluorescence overlays for TEX264-GFP-positive autophagosomes 2, 3, 4 and 5**. n**, 3D segmentations, zoomed-in 11500X TEM images overlaid with GFP cryo-fluorescence signal and cryo-CARE denoised slices for autophagosomes 3, 4, and 5. All images are rotated right by 90 degrees compared to their respective full 11500X overlays in **m**. Autophagosome 3 and 4 are microtubule-linked, while autophagosome 5 is distant from the microtubules (MT). **o**, Plot showing thickness of all tomograms (n=32) analysed in this work. Each dot represents a tomogram. Black dots indicate the tomograms corresponding to the five TEX264-GFP-positive autophagosomes (AP) shown in this study.

TEX264-GFP/mCh-LC3B trafficking led us to ask whether ER-containing autophagosomes could be visualized in projections by cryo-ET. iNeurons grown on EM grids were plunge-frozen at day 18, followed by cryo-fluorescence microscopy and cryo-ET of thin neuronal projections (Fig. 5a). We used an unbiased approach to survey axonal projections for autophagic structures, which we identified directly in TEM images. We then correlated autophagosome positions in the TEM images with cryo-fluorescence data to evaluate coincidence with fluorescence signal. Finally, neural network-based segmentation revealed the cargo and cellular surroundings of the captured autophagosomes, yielding 37 autophagosomes captured in situ in projections (Fig. 5b–h, Extended Data Fig. 3a–f and Methods). Many autophagic structures (24 out of 37) were proximal to microtubules within the axon, as would be expected during trafficking to the soma (Fig. 5c–h and Extended Data Fig. 3d,f). Interestingly, tubular ER is present as cargo inside 21 of the 37 autophagosomes analysed (Fig. 5b–h, Extended Data Fig. 3e,f and Supplementary Video 4), and regions with GFP signal were coincident with ER tubule-containing autophagic structures (Fig. 5b,d,g and Extended Data Fig. 3g–n). In particular, five out of 21 ER tubule-containing autophagic structures coincided with a TEX264-GFP signal, and four of the five were adjacent to microtubules. Absence of GFP-TEX264 signal in a subset of ER-containing autophagosomes may be due to a low expression level of lentiviral-transduced TEX264-GFP in individual iNeurons or due to selective capture by alternative ER-phagy receptors. Although TEX264-GFP displayed a punctate and distinct signal, the mCh-LC3B cryo-fluorescence signal appeared diffuse and could not be reliably used for correlation (Extended Data Fig. 3m and Methods). Notably, autophagosomes that did not contain tubular ER cargo never coincided with TEX264-GFP signal (Fig. 5b). Together, these data confirm capture of TEX264-GFP-positive ER by autophagy within axons and demonstrate selective ER-phagy in iNeurons.

### A genetic toolkit for ER-phagy receptor analysis in iNeurons

**Fig. 6:**
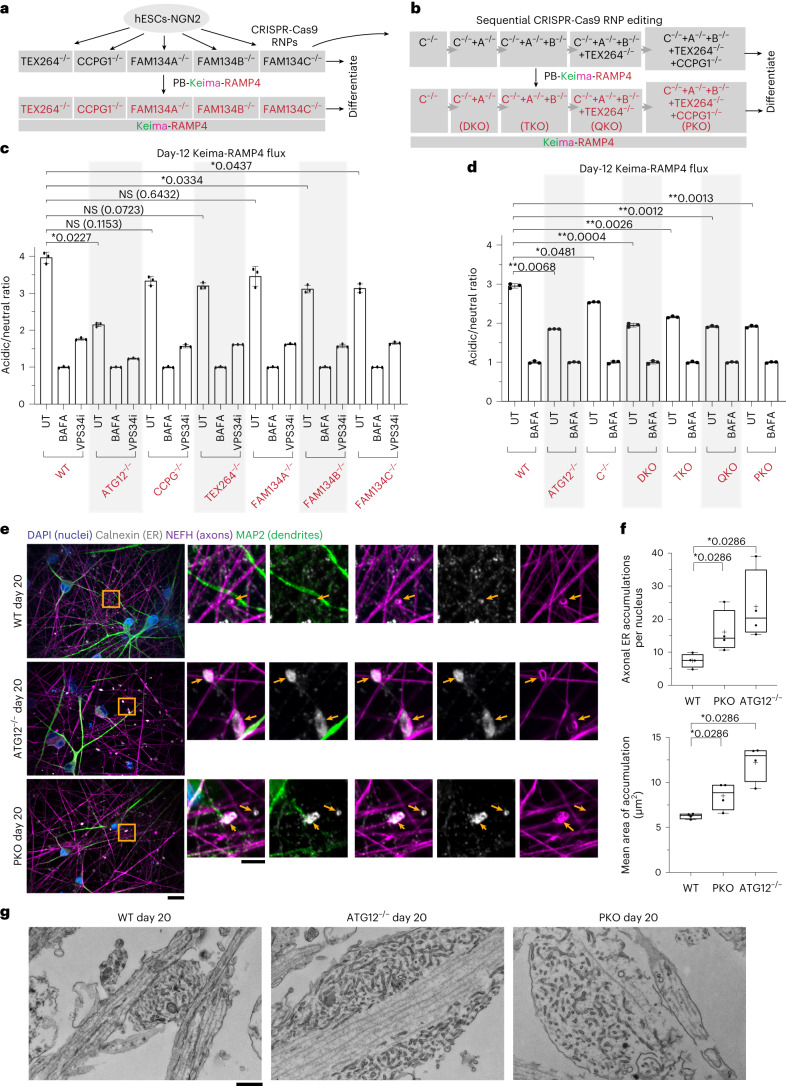
Combinatorial regulation of ER clearance via ER-phagy receptors during neurogenesis in vitro. **a**,**b**, A toolkit for analysis of ER-phagy receptors. hESCs were subjected to CRISPR-Cas9 gene editing to delete individual (**a**) or multiple (**b**) receptors. Keima-RAMP4 was expressed in each of the mutant hESCs, before analysis during differentiation. **c**,**d**, Ratiometric analysis of Keima-RAMP4 flux in the indicated WT or mutant hESCs was measured by flow cytometry at day 12 of differentiation. The ratio of acidic to neutral Keima fluorescence was normalized to samples treated with BAFA (100 nM) for 4 h. Each measurement reflects biological triplicate measurements. Data are presented as mean values ± s.d. *P < 0.05; **P < 0.01; NS, not significant; Brown–Forsythe and Welch one-way ANOVA and Dunnett’s T3 multiple comparisons test. **e**,**f**, PKO iNeurons accumulate aberrant ER structures, particularly in axons. Day 20 iNeurons of the indicated genotypes were immunostained with α-calnexin (ER, white), α-MAP2 (dendrites, green), α-NEFH (axons, magenta) and with DAPI (nuclei, blue) (**e**). A further zoomed-in region of the WT axonal region is also shown in Fig. 2 to compare only WT and ATG12^−/−^. Scale bars, 25 μm (full images) and 5 μm (zooms). The number of axonal ER accumulations per nucleus (**f**, top) or mean area of ER accumulation (**f**, bottom) are represented with min-to-max box-and-whiskers plots (the box represents the 25th to 75th percentiles, whiskers extend from min to max values, the line represents the median and + the mean). Four points shown for each WT or KO condition represent the measured values from four independent differentiations. *P < 0.05; two-sided Mann–Whitney test. **g**, TEM images of sections though WT, ATG12^−/−^ and PKO axons from one differentiation experiment containing enlarged structures with areas of ER membranes. Scale bar, 500 nm.

**Extended Data Fig. 4:**
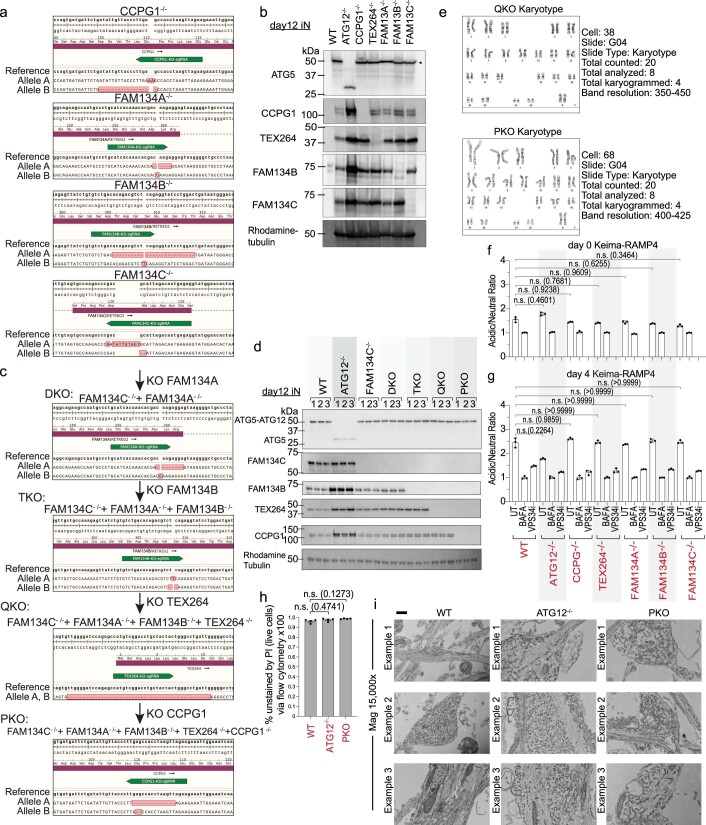
Generation of a genetic toolkit for functional analysis of ER-phagy receptors in iNeurons. **a**, MiSeq analysis of single ER-phagy receptor mutants in hESCs. The green highlights the target of the CRISPR gRNA. The sequence of the major MiSeq output is indicated for each allele. **b**, Immunoblot validation of targets knockout clones at day 12 of differentiation. Confirmation immunoblots for protein deletion in these different genetic backgrounds were performed for each proteomics experiment. Cell extracts were subjected to immunoblotting with the indicated antibodies, employing a Rhodamine-labelled α-tubulin as loading controls. *, position of the ATG12-ATG5 conjugate. **c**, MiSeq analysis of combinatorial ER-phagy receptor mutants in hESCs, as performed for the single knockouts in a. **d**, Immunoblot validation of targets in combinatorial knockout clones at day 12. Cell extracts were subjected to immunoblotting as in **b**. Confirmation immunoblots for protein deletion in these different genetic backgrounds were performed for each proteomics experiment. **e**, Karyotype analysis of QKO and PKO hESCs revealed no detectable alterations in chromosome number. **f**, Ratiometric flow cytometry analysis of Keima-RAMP4 flux was measured in WT, ATG12^−/−^, or the indicated ER-phagy receptor knockout ES cells (day 0 of differentiation). The ratio of acidic to neutral Keima fluorescence was normalized to samples treated with BAFA (100 nM) for 4 h prior to analysis, and where indicated, cells were cultured with VPS34i prior to analysis. Each measurement (represented by a point) reflects a biological triplicate sample. **g**, As in panel **f**, but at day 4 of differentiation to iNeurons. For **f**, and **g**, Data are presented as mean values +/−SD. n.s., not significant; Brown–Forsythe and Welch One-way ANOVA and Dunnett’s T3 multiple comparisons test. **h**, Day 12 iNeurons treated Propidium iodine staining and were analysed via flow cytometry. The same gating strategy for live cells was applied to all genotypes, as was done in all Keima flux experiments. Mean values +/-SEM of the percent of live cells (not stained with PI) is displayed. N=3 biological replicates; n.s. not significant; two sided Mann–Whitney test. **i**, Examples of enlarged axonal structures from WT, ATG12^−/−^ and PKO day 30 iNeurons containing dense tubular ER, as visualized by TEM. Some of these examples were also shown in Fig. 2 to compare only WT and ATG12^−/−^. Three independent examples are shown. Scale bar, 500nm. Source numerical data, flow cytometry gating strategy, and unprocessed blots are available in source data. Source data

To systematically explore the contributions of individual ER-phagy receptors to ER remodelling during iNeuron differentiation^35^, we used gene editing to create single-knockout hESCs for FAM134A, FAM134B, FAM134C, TEX264 or CCPG1, which were confirmed by sequence analysis and immunoblotting (Fig. 6a and Extended Data Fig. 4a,b). To address redundancy among the ER-phagy receptors^10^, we sequentially edited FAM134C^−/−^ cells to create double, triple, quadruple and penta receptor knockout lines: FAM134A/C^−/−^ (DKO), FAM134A/B/C^−/−^ (TKO), FAM134A/B/C/TEX264^−/−^ (QKO) and FAM134A/B/C/TEX264/CCPG1^−/−^ (PKO) (Fig. 6b and Extended Data Fig. 4c,d). Sequential deletion of ER-phagy receptors was verified by sequence analysis and immunoblotting; QKO and PKO mutants displayed normal karyotypes (Extended Data Fig. 4c–e). PKO iNeurons differentiated efficiently, displayed viability parameters equivalent to WT iNeurons, and displayed no evidence of ER stress as assessed by ATF4 or *XBP1s* induction (Extended Data Fig. 2a–f and Supplementary Table 4). Each mutant cell line was reconstituted with Keima-RAMP4 to measure ER-phagic flux (Fig. 6a,b).

### ER-phagy receptor control of ER-phagic flux in iNeurons

To examine individual receptor contributions to ER-phagy during differentiation, we measured Keima-RAMP4 flux in receptor mutant cells at day 0, 4 or 12 of differentiation using flow cytometry (Fig. 6c and Extended Data Fig. 4f,g). As expected, the Keima-RAMP4 flux increased from 2.5- to 4.0-fold in WT cells at days 4 and 12 of differentiation, which was substantially reduced in day-12 ATG12^−/−^ iNeurons (Fig. 6c and Extended Data Fig. 4f,g). All single mutants displayed Keima-RAMP4 flux comparable to WT at day 4 and >80% of WT at day 12 (Fig. 6c and Extended Data Fig. 4g). However, upon elimination of FAM134A/C, the level of Keima-RAMP4 flux approached that seen with ATG12^−/−^ cells at day 12 of differentiation, with a slight further reduction upon removal of additional receptors (Fig. 6d). Importantly, the reduction in ER-phagic flux measured for ATG12^−/−^ and PKO iNeurons was not skewed due to differential cell viability (Extended Data Fig. 4h).

Consistent with defective ER turnover, day-20 PKO iNeurons displayed more abnormally enlarged α-calnexin-marked ER structures in α-NEFH-positive axons (Fig. 6e). The number and size of these structures were intermediate between WT and ATG12^−/−^ iNeurons (Fig. 6f). TEM of thin sections through axons of PKO iNeurons also revealed examples of frequent dilated structures rich in tubular ER, albeit smaller than that observed in ATG12^−/−^ axons (Fig. 6g and Extended Data Fig. 4i).

### Combinatorial receptor control of the neuronal ER proteome

**Fig. 7:**
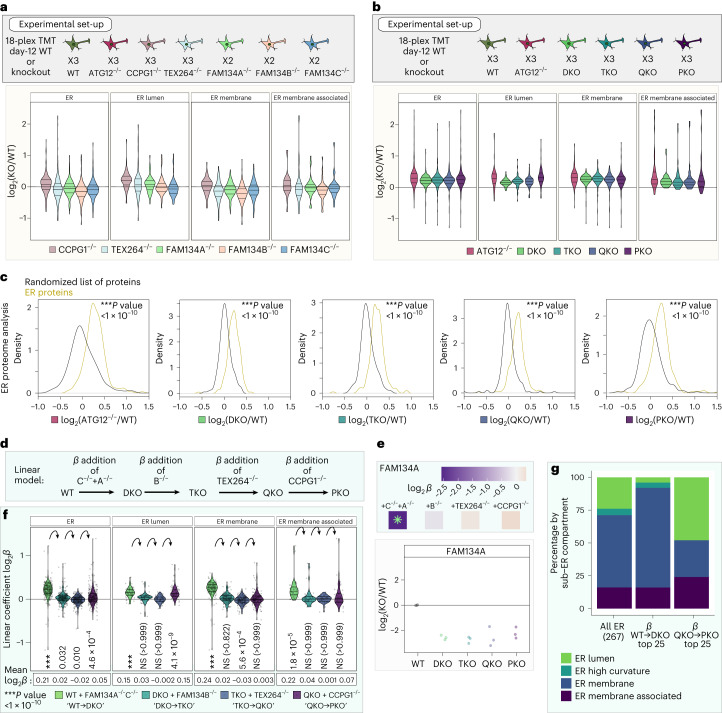
Selectivity of ER-phagy receptors in ER remodelling in iNeurons revealed by combinatorial multiplexed proteomics. **a**, Scheme depicting an 18-plex TMT experiment examining the total proteomes of the indicated single ER-phagy receptor mutant day-12 iNeurons. Violin plots (lower panel) depicting log_2_FC (mutant/WT) for the indicated classes of ER proteins in single-mutant iNeurons (day 12) are shown in the lower plot. **b**, Scheme depicting an 18-plex TMT experiment examining the total proteomes of the indicated combinatorial ER-phagy receptor mutant day-12 iNeurons. Violin plots (lower panel) depicting log_2_FC (mutant/WT) for the indicated classes of ER proteins in combinatorial mutant iNeurons (day 12) are shown in the lower plot. **c**, log_2_FC (mutant/WT) distributions of ER proteins compared to randomized selections of the same number of proteins (100 iterations). *P* values for each comparison are calculated with a Kolmogorov–Smirnov test (two-sided). **d**, Application of a linear model to identify selective cargo for individual ER-phagy receptors via quantitative proteomics. In the linear model, a coefficient FC (*β*) is calculated for sequential loss of ER-phagy receptors starting from WT to DKO, then DKO to TKO, then TKO to QKO, then QKO to PKO. **e**, *β* coefficient values (top panel) and log_2_FC (lower panel) for FAM134A. The green asterisk in the top panel indicates a significant change (adjusted *P* value of <0.05) in the *β* coefficient for that mutant. *P* values for *β* values extracted from the linear model are calculated with a Student’s *t*-test (two-sided), with multiple hypothesis correction using the Benjamini–Hochberg method. This analysis is distinct from traditional comparisons between each mutant and WT (lower panel). **f**, Violin plots depicting the *β* coefficient FC for the indicated classes of ER proteins. *P* values for each comparison are calculated with a one-sided Wilcoxon test with Bonferroni correction. **g**, Top 25 accumulating ER proteins in WT to DKO and QKO to PKO and their respective ER compartment compared to the landscape of the whole ER. The TMT ratio check and normalization are available in the source data. Source data

**Extended Data Fig. 5:**
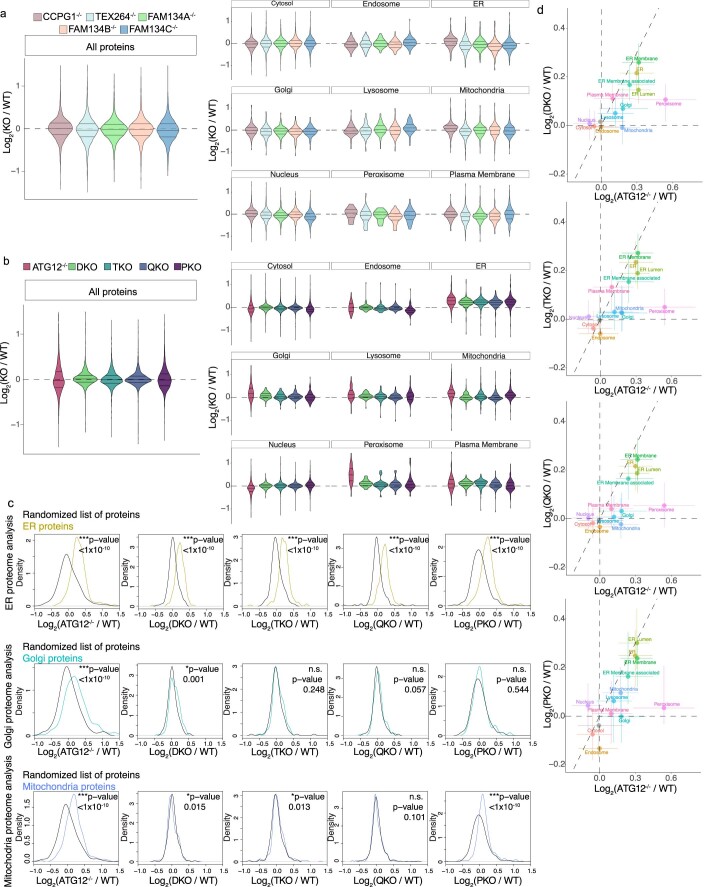
Combinatorial analysis of ER remodelling via ER-phagy receptors during neurogenesis in vitro. **a**, Violin plots for changes in individual organelle abundance in the indicated single ER-phagy knockout iNeurons (day 12). **b**, Violin plots for changes in individual organelle abundance in the indicated combinatorial ER-phagy knockout iNeurons (day 12). **c**, Log_2_FC (mutant/WT) distributions of ER proteins (top), Golgi proteins (middle), or mitochondria proteins (bottom) compared to randomized selections of the same number of proteins (100 iterations). p-values for each comparison are calculated with a Kolmogorov–Smirnov Test (two-sided). The ER protein section of the figure (top panel) is reproduced from Fig. 7c. **d**, Correlation plots for changes in organelle abundance (Log_2_FC) comparing DKO, TKO, QKO and PKO log_2_FCs from WT individually with ATG12^−/−^ log_2_FCs from WT.

**Extended Data Fig. 6:**
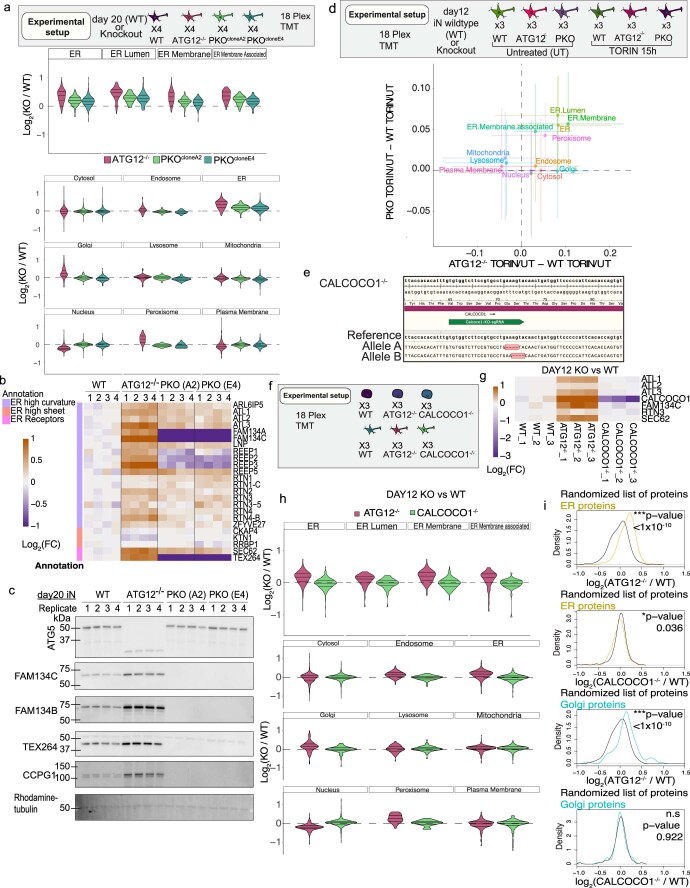
Analysis of ER-proteome remodelling in PKO and CALCOCO1^−/−^ iNeurons. **a**, Proteomic analysis of two PKO clones (A2 and E4) in parallel with ATG12^−/−^ and WT iNeurons (day 20). The upper panel provides a schematic of the TMT multiplex approach employed. n = 4 biological replicates. Middle panel displays Log_2_FC for ER protein and selected ER protein categories. Lower panel displays Log_2_FC for the indicated organelles. **b**, Heatmap for Log2FC values for the indicated proteins from the experiment in panel **a**. **c**, Immunoblot validating deletion of FAM134B, FAM134C, TEX264, and CCPG1 in both clone A2 and E4 for the PKO mutant. **d**, Modulation of iNeuron proteome in response to inhibition of MTOR with Torin1 (100 nM,15 h). Upper panel shows a schematic of the experimental set-up employing TMT based proteomics to quantity alterations in the proteome if WT or ATG12^−/−^, PKO iNeurons. Lower panel: Correlation plots comparing the effect of Torin1 on organelles of ATG12^−/−^ cells relative to WT cells and PKO cells relative to WT cells. **e**, MiSeq analysis of a CALCOCO1^−/−^ H9 hESC clone, showing the position of the gRNA used for CRISPR-Cas9 deletion, and the position of out of frame deletions in the two alleles of CALCOCO1. **f**, Schematic showing the TMT total proteome strategy for analysis of the effect of CALCOCO1 deletion on organelle abundance in hESCs and day 12 iNeurons (n=3 biological replicates). ATG12^−/−^ cells were included as a positive control. **g**, Heatmap of Log_2_FC values for selected proteins from the TMT experiment outlined in panel **f**, which also demonstrates loss of the CALCOCO1 protein in the CALCOCO1^−/−^ iNeurons. **h**, Violin plots (Log_2_FC) for the indicated organelles (top panel) and selected classes of ER proteins (lower panel) for ATG12^−/−^ and CALCOCO1^−/−^ iNeurons (day 12). Loss of CALCOCO1 does not affect the abundance of any of the organelles tested. **i**, Comparison of Log_2_FC (mutant/WT) distributions of ER proteins (top) or Golgi proteins (bottom) to distributions of randomized selections of the same number of proteins (100 iterations). p-values for comparisons were calculated with a Kolmogorov–Smirnov Test (two-sided). Unprocessed blots are available in source data. Source data

We next sought to define how the entire ER proteome is remodelled by individual ER-phagy receptors during differentiation and to unmask the potential selectivity of receptors for specific clients. We performed 18-plex TMT quantitative proteomics using single (Fig. 7a and Supplementary Table 5) and combinatorial (Fig. 7b and Supplementary Table 3) ER-phagy receptor mutants at day 12 of differentiation. ATG12^−/−^ iNeurons were included as a control for autophagy-dependent stabilization. The abundance of organelles at the global level, including ER, was largely unaffected in single ER-phagy mutants, as suggested by violin plots for individual organelle proteomes (Fig. 7a and Extended Data Fig. 5a). In contrast, and consistent with a more pronounced effect on Keima-RAMP4 flux and axonal ER accumulation, combinatorial mutants displayed an overall increase in ER protein abundance comparable to that seen in ATG12^−/−^ (Fig. 7b–c and Extended Data Fig. 5b–d). The distribution of ER proteins in ATG12^−/−^ or the DKO to PKO mutants significantly deviates from a randomized selection of proteins (randomized control) of the same number of proteins (Fig. 7c). However, the combinatorial mutants did not affect the distribution of the Golgi proteome, known to be regulated by loss of ATG12 in this system^25,36^, consistent with a specific role of ER-phagy receptors in ER turnover (Extended Data Fig. 5b–d). Importantly, we confirmed, using quantitative proteomics, that ER protein accumulation was maintained in later-stage day-20 ATG12^−/−^ and PKO iNeurons, corresponding to the time employed for several imaging experiments described above, and we confirmed that this ER accumulation occurs in an independent PKO clone (Extended Data Fig. 6a–c and Supplementary Table 6).

In cancer cell lines, MTOR inhibitor Torin1 induces a starvation-like response, leading to clearance of ER (among other organelles) and proteins via autophagy^37^. To further probe the susceptibility of ER to selective turnover via general autophagy as compared to selective ER-phagy, we examined the organelle and proteome abundance in iNeurons treated with Torin1 for 15 h. In ATG12^−/−^ iNeurons, organelle clearance was blunted when compared with WT iNeurons (Extended Data Fig. 6d and Supplementary Table 7). In contrast, PKO iNeurons treated with Torin1 demonstrated a defect in the clearance of ER proteins (similar to ATG12^−/−^), whereas other organelles were largely unaffected (Extended Data Fig. 6d and Supplementary Table 7), consistent with selective roles for ER-phagy receptors. The ubiquitin-binding autophagy receptor CALCOCO1 has been reported to function as a soluble receptor for both Golgi and ER turnover in response to nutrient stress^38,39^, although a general function in ER- or Golgiphagy has been questioned^36^. While CALCOCO1 accumulated in ATG12^−/−^ iNeurons during differentiation (Fig. 1c), iNeurons lacking CALCOCO1 displayed no global accumulation of ER or Golgi proteomes, unlike ATG12^−/−^ iNeurons examined in parallel (Extended Data Fig. 6e–i and Supplementary Table 8). Thus, CALCOCO1 alone is not necessary for ER or Golgi maintenance in this system.

### Quantitative modelling of ER proteome remodelling via ER-phagy

**Extended Data Fig. 7:**
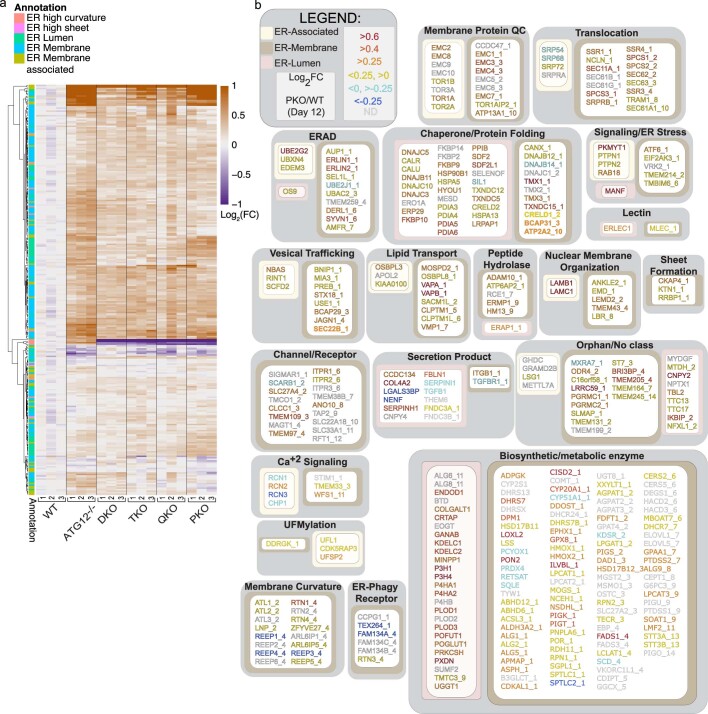
Overview of ER proteome remodeling via ER-phagy receptors during neurogenesis in vitro. **a**, Changes in the abundance (Log_2_FC) of the ER proteome (267 detected proteins) during conversion in ATG12^−/−^ or combinatorial ER-phagy receptor knockout iNeurons (day 12) are shown as heatmaps. Annotations of the type of ER protein are indicated by the relevant colours. **b**, Landscape of the ER proteome and the effect of deletion of five ER-phagy receptors (FAM134A/B/C, TEX263 and CCPG1) on accumulation of individual proteins. The ER proteome (359 proteins, Supplementary Data Table 1) is organized into functional modules, and protein attributes (involved in ER membrane curvature, ER-associated, ER-membrane, ER-Lumen or ER-phagy receptor) are indicated by the respective outline box colour (see inset legend). For proteins with transmembrane segments, the number of segments is indicated after the protein name (_1, _2, etc) based on data in Uniprot. The text of each protein name is colored based on day12 PKO vs WT Log_2_FC (see inset legend). (Supplementary Data Table 3).

**Extended Data Fig. 8:**
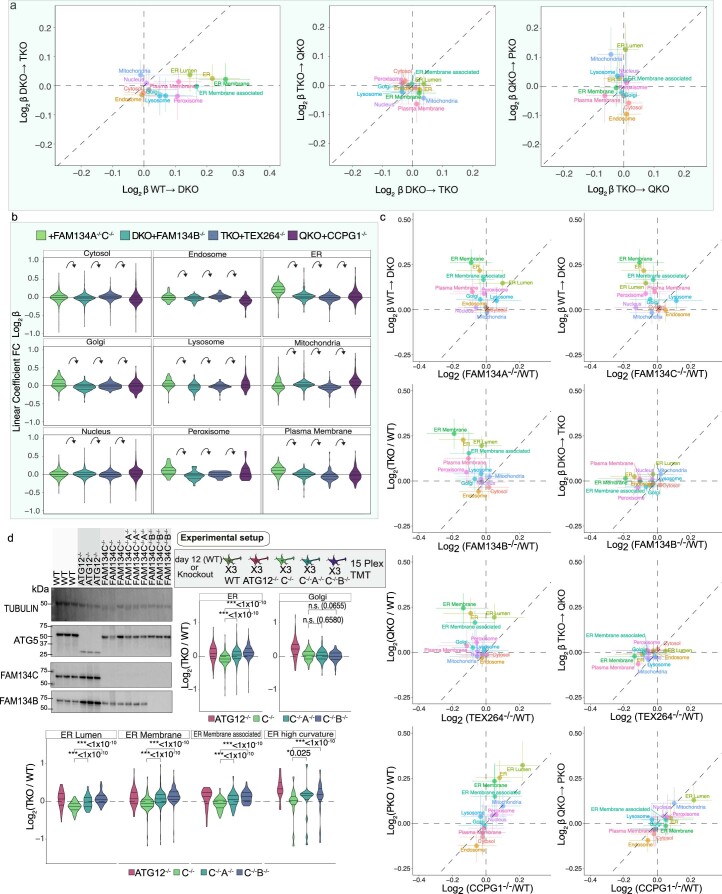
Application of a linear model for alterations in ER proteome abundance in sequential ER-phagy receptor knockout cells during iNeuron differentiation. **a**, Effect of sequential ER-phagy receptor deletion on the β coefficient values for individual organelles measured by quantitative proteomics in day 12 iNeurons. **b**, Violin plots reflecting changes in β coefficient values for individual organelles measured by quantitative proteomics in day 12 iNeurons. Curved arrows reflect sequential removal of the indicated ER-phagy receptor. **c**, Correlation plots for the indicated β coefficient or Log_2_FC plots comparing organelle abundance for combinatorial or single ER-phagy deletion iNeurons. **d**, Experiment characterizing FAM134C^−/−^, FAM134A/C^−/−^, and FAM134B/C^−/−^ day 12 iNeurons with ATG12^−/−^ iNeurons included as a control. Top left, Immunoblot of extracts from iNeurons of the indicated genotypes (n = 3) were probed with α-ATG5, α-FAM134B, α-FAM134C, or α-tubulin. ATG12^−/−^ cells display loss of the ATG12-ATG5 conjugate as observed with α-ATG5. Top right, experimental scheme for multiplexed total proteome analysis of FAM134C^−/−^, FAM134A/C^−/−^, and FAM134B/C^−/−^ iNeurons and ER proteome specific violin plots derived from this analysis. Bottom, organelle-specific violin plots from the experiment. p-values for comparisons between violins were calculated using paired two sided Wilcoxon tests. Unprocessed blots are available in source data. Source data

The behaviour of the ER proteome in single and combinatorial ER-phagy mutant iNeurons, with a range of altered protein abundances occurring across the ER proteome (Fig. 7a,b and Extended Data Fig. 7a,b), suggests both redundancy and selectivity for client turnover by receptors. To further probe this underlying specificity, we employed a linear model that measures the sequential effect of (1) FAM134A/C, (2) FAM134B, (3) TEX264 and (4) CCPG1 deletion. The model measures the positive or negative log_2_FC values, comparing each step, and assigns these changes with positive or negative *β* coefficients: (1) *β*^WT→DKO^; (2) *β*^DKO→TKO^, (3) *β*^TKO→QKO^ and (4) *β*^QKO→PKO^ (Fig. 7d, Extended Data Fig. 8a,b and Methods). The behaviour of FAM134A is an example of the applicability of the model (Fig. 7e): *β*^WT→DKO^ was strongly negative (−2.5) and significant (indicated by the asterisk), consistent with its deletion in the DKO mutant compared to WT, but *β* coefficient values in subsequent deletions was near zero and not significant, as expected, because FAM134A remains deleted and therefore remains at the same abundance throughout the remainder of the allelic series.

Global analysis revealed an increase in the mean *β*^WT→DKO^ coefficients for the ER proteome (0.21), which was primarily reflected in alterations in the abundance of ER-membrane and ER-lumen proteins (Fig. 7f). *β*^DKO→TKO^ and *β*^TKO→QKO^ coefficients reflecting the further deletion of FAM134B and TEX264, respectively, are near zero for the ER proteome as a whole and for specific ER subregions (Fig. 7f and Extended Data Fig. 8a), suggesting modest or no contributions to ER turnover in this context. In contrast, the mean *β*^QKO→PKO^ coefficient resulted in an increase (0.15) for a cohort of ER lumenal proteins (Fig. 7f and Extended Data Fig. 8a), indicating that CCPG1 and FAM134A/C independently control the abundance of a set of lumenal proteins based on either the magnitude of abundance change or protein identity, as explored further in the following. The effect of CCPG1 on lumenal ER protein abundance is further demonstrated by organelle point plots comparing *β*^TKO→QKO^ and *β*^QKO→PKO^, with significant displacement of ER lumen off the diagonal (Extended Data Fig. 8a). We next compared the organelle proteome abundance changes that occur when an individual deletion is made from the WT background to the organelle proteome abundance changes that occur when the same deletion is added to the sensitized background reflected by the *β* value for that deletion in the combinatorial deletion series (Extended Data Fig. 8c). The effect on the ER of single deletion of CCPG1 or of deletion of CCPG1 in the QKO to create the PKO suggests that CCPG1 can act alone as an ER-phagy receptor to clear luminal proteins during neuronal differentiation. However, the FAM134 family of receptors only yielded an increase in the ER network and different ER compartments when the FAM134 family was deleted in combination.

The finding that the combined loss of FAM134A and C leads to accumulation of a cohort of ER proteins and that the ER proteome was not substantially altered upon further deletion of FAM134B led us to ask whether FAM134A and B are functionally equivalent in this setting. We generated FAM134B/C^−/−^ cells and performed multiplexed proteomics comparing FAM134C^−/−^, FAM134A/C^−/−^ (DKO) and FAM134B/C^−/−^ iNeurons (day 12) (Extended Data Fig. 8d and Supplementary Table 9). Global ER and ER-membrane protein abundance in particular also increased in FAM134B/C^−/−^ iNeurons relative to FAM134C^−/−^ iNeurons (Extended Data Fig. 8d). Taken together, this suggests that FAM134 copy number, rather than the identity of the specific isoform, underlies ER proteome remodelling in this context.

### ER-phagy receptor substrate specificity

**Fig. 8:**
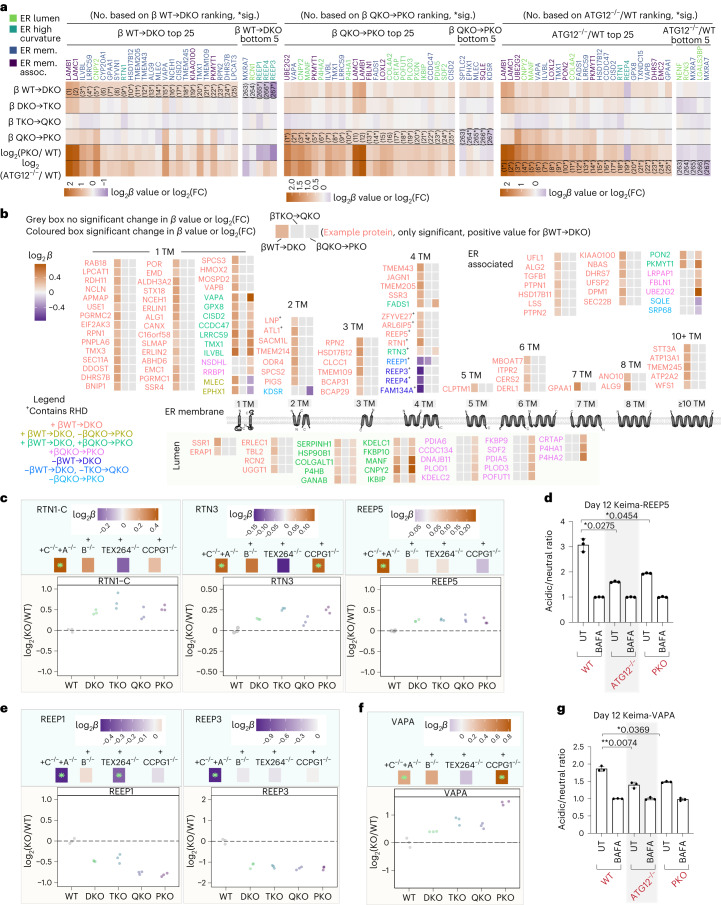
ER-phagy receptor remodelling of the ER proteome landscape and ER-phagy receptor cargo specificity during iNeuron differentiation. **a**, Top 25 accumulated and bottom five depleted ER proteins ranked on WT to DKO *β*-coefficient values (left panel), QKO to PKO *β-*coefficient values (middle panel) or on log_2_FC (ATG12^−/−^/WT). **b**, ER-associated, ER-membrane or ER-lumenal distribution and predicted TM character of ER proteins with significant *β*-coefficient values (*adjusted *P* value < 0.05) in WT to DKO (111 up, 4 down), TKO to QKO (1 down) and QKO to PKO (39 up, 5 down). Zero proteins were significant in DKO to TKO. Each protein name is coloured based on whether there is a significant change in these steps in the allelic series, as shown in the legend. The corresponding *β*-coefficient value heatmap for each protein is coloured in if there is a significant change and left blank if there is no significant change at that step in the allelic series (see legend). *P* values for the *β* values extracted from the linear model are calculated with a Student’s *t*-test (two-sided), and multiple hypothesis correction using the Benjamini–Hochberg method. **c**, Examples of ER-shaping proteins with significant *β* coefficients that accumulate at one or more steps in the allelic series. *β*-coefficient values (top panels) and log_2_FC (lower panels) are shown for single proteins, including RTN1-C, RTN3 and REEP5. **d**, Keima-REEP5 flux measurements in WT, ATG12^−/−^ and PKO iNeurons (day 12) using acidic/neutral ratios in the presence of BAFA for normalization. **e**, As in **c**, but for proteins REEP1 and REEP3 (ER-shaping proteins with significant *β* coefficients that decrease). **f**, As in **c** and **e**, but for VAPA (an ER-membrane protein that forms contact sites with other organelles). **g**, Autophagic flux assay for Keima-VAPA in WT, ATG12^−/−^ or PKO iNeurons (day 12). For the individual protein plots in **c**, **e** and **f**, the green asterisks in the top panels indicate a significant change (*adjusted *P* value < 0.05) in *β* coefficients for each mutant, Student’s *t*-test (two-sided), with multiple hypothesis correction using the Benjamini–Hochberg method. For autophagic flux experiments using Keima-REEP5 and Keima-VAPA, *n* = 3, data are presented as mean values ± s.d. *P < 0.05; **P < 0.01; Brown–Forsythe and Welch one-way ANOVA and Dunnett’s T3 multiple comparisons test.

**Extended Data Fig. 9:**
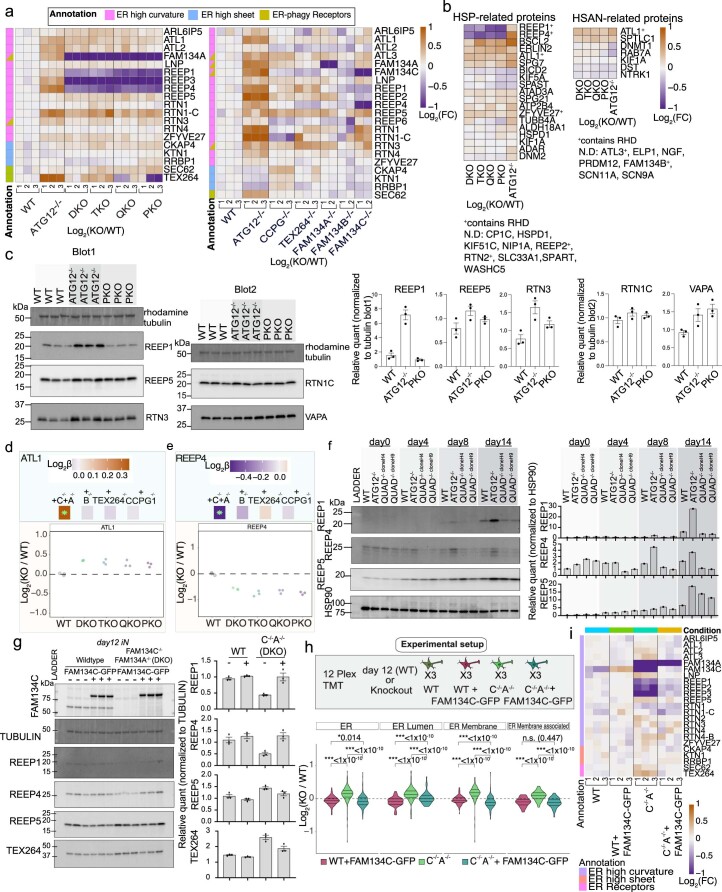
Differential regulation of ER membrane shaping and disease-linked proteins upon loss of ER-phagy receptors. **a**, Heatmaps displaying Log_2_ FC values for selected ER-shaping proteins for the indicated iNeuron genotypes. **b**, Heatmaps displaying Log_2_ FC values for Hereditary Spastic Paraplegia (HSP)-linked proteins or Hereditary Sensory and Autonomic Neuropathy (HSAN)-linked proteins for the indicated iNeuron genotypes. **c**, Immunoblot of extracts from WT, ATG12^−/−^, or PKO day 12 iNeurons (n=3) were probed with α-REEP1, α-REEP5, α-RTN3 or α-tubulin as a loading control (blot1) or were probed with α-RTN1C, α-VAPA or α-tubulin as a loading control (blot2). The relative levels of each protein to tubulin were quantified (right). Data are presented as mean values +/-SEM. **d-e**, Further examples ER shaping proteins that accumulate (**d**) or decrease (**e**) with additional ER-phagy receptor knockout. **f**, Immunoblot of cell extracts isolated from the indicated time point during differentiation (n=1), using α-HSP90 as a loading control. The relative levels of proteins were quantified (right). **g**, Immunoblot of extracts from WT or FAM134A/C^−/−^ (DKO) iNeurons with or without expression of FAM134C-GFP using a PiggyBac vector (n = 3). Blots were probed with α-TEX264, α-REEP1, α-REEP4 or α-tubulin as a loading control. The relative levels of proteins to tubulin were quantified in the lower panel. Data are presented as mean values +/-SEM. **h**, Experimental scheme for multiplexed total proteome analysis of WT or FAM134A/C^−/−^ with or without expression of FAM134C-GFP iNeurons (n = 3) and ER protein-specific violin plots derived from this analysis. p-values for comparisons between violins were calculated using paired two sided Wilcoxon tests**. i**, Heatmap displaying Log_2_ FC values for selected ER-shaping proteins for the indicated iNeuron genotypes. Source numerical data and unprocessed blots are available in source data. Source data

To directly examine the substrate selectivity of the ER-phagy receptors, we first explored the top 25 ranked proteins with positive *β* coefficients for both *β*^WT→DKO^ and *β*^QKO→PKO^. When compared with all ER proteins, those with positive *β*^WT→DKO^ coefficients were particularly enriched in ER-membrane proteins, whereas proteins with positive *β*^QKO→PKO^ coefficients were enriched in lumenal proteins (Fig. 7g). Heatmaps revealing the identity of these top accumulators highlight the degree of change in the *β* coefficients, with significantly changing proteins marked with an asterisk (*adjusted *P* value < 0.05; positive or negative *β* coefficients, Fig. 8a). The extent of accumulation of these top-ranked proteins in PKO cells was similar to that seen with ATG12^−/−^ iNeurons (Fig. 8a), indicating that the PKO mutant closely approximates the biochemical phenotype of ATG12 deficiency for ER turnover. Globally, we identified 84 membrane proteins with significantly (*adjusted *P* value < 0.05) positive or negative *β*^WT→DKO^ coefficients, which were distributed across multiple functional categories and contained varying numbers of TM segments (Fig. 8b and Supplementary Table 3). Given that several of the ER-shaping proteins with RHDs are within this group of significant changers (Fig. 8b) and that ATG12 deficiency strongly affects ER-shaping proteins with RHDs (Fig. 1e), we examined this class of proteins further. In addition, we examined ER proteins that are specifically related to two neurological disorders, hereditary spastic paraplegia (HSP) and hereditary sensory and autonomic neuropathy (HSAN) (a subset of which are also ER curvature-shaping proteins that contain RHDs). Heatmaps of log_2_FC values for these specific ER proteins are provided in Extended Data Fig. 9a,b), and immunoblotting of selected proteins confirmed accumulation both in ATG12^−/−^ and PKO iNeurons (Extended Data Fig. 9c). First, we found that a subset of ER-curvature proteins specifically increased in *β*^WT→DKO^, including RTN1-C (log_2_FC = 0.44; Fig. 8a–c and Extended Data Fig. 9c,d). Similarly, REEP5 accumulated—albeit to a lesser extent—in PKO iNeurons (Fig. 8b,c and Extended Data Fig. 9a,c), and Keima-REEP5 flux measurements revealed decreased flux in PKO cells, approaching that observed in ATG12^−/−^ iNeurons (Fig. 8d). Second, a distinct set of RHD proteins (REEP1, REEP3 and REEP4) decrease in abundance, and display negative *β* coefficients for DKO (Fig. 8a,b,e and Extended Data Fig. 9a–c,e). REEP1 also further decreases upon deletion of TEX264, as indicated by a significant negative *β* coefficient and log_2_FC (Fig. 8e). Because members of the RHD protein family (for example, REEP1) are strongly upregulated during iNeuron differentiation (Fig. 1a–d and Extended Data Fig. 9f), alterations in abundance across the REEP family indicate distinct pathways for controlling ER shape remodelling for neurons, specifically via ER-phagy. Whereas the collective ER proteome did not increase with the single FAM134C deletion, abundance alterations for ER-shaping proteins specifically were observed with just the single deletion (Extended Data Fig. 9a), indicating that FAM134C probably contributes substantially to the differential regulation of shaping proteins during neurogenesis (Fig. 8c). Interestingly, ATG12^−/−^ iNeurons display increases in abundance for all REEP proteins, indicating that a broad block to autophagy can mask otherwise distinct proteome remodelling events relevant to an individual ER-phagy receptor (Extended Data Fig. 9a–c,f).

To examine whether the loss of specific REEP family members is directly due to the loss of FAM134, we ectopically expressed FAM134C-GFP in either WT or DKO hESCs using a PiggyBac vector and converted the cells to iNeurons (day 12) (Extended Data Fig. 9g–i). Immunoblotting of the cell extracts revealed that the increase in TEX264 abundance and the decrease in REEP1 or REEP4 in DKO cells is reversed by the re-introduction of FAM134C-GFP (Extended Data Fig. 9g,i). Similarly, proteomics revealed that FAM134C-GFP expression in DKO iNeurons reversed the global accumulation of a cohort of ER proteins, in particular ER-membrane proteins (Extended Data Fig. 9h and Supplementary Table 10). Proteomics also validated rescue in the expression of REEP1/2/3; REEP4 was not detected in this specific experiment (Extended Data Fig. 9i).

**Extended Data Fig. 10:**
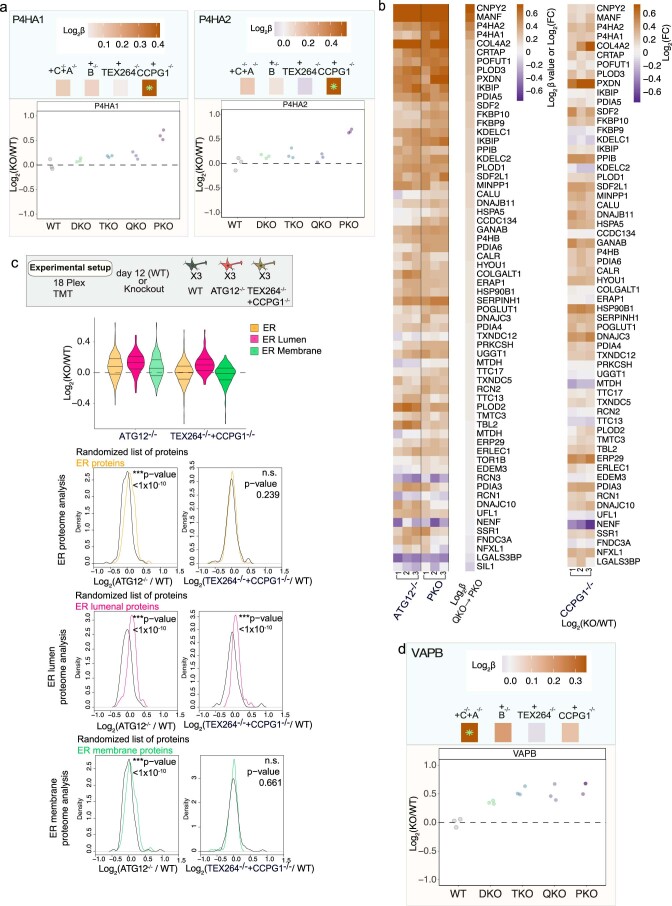
Differential regulation of ER membrane shaping proteins upon loss of ER-phagy receptors. **a**, ER lumenal proteins that accumulate with additional ER-phagy receptor knockout. Top panels are β coefficient values and lower panels are Log_2_FC; green asterisks in β coefficient for single protein heatmaps indicate significant change (adjusted p-value < 0.05) in β coefficient. **b**, ER lumenal protein heatmaps reflecting the change in abundance (Log_2_FC) for deletion of ATG12 or PKO, reflecting β coefficient values for QKO to PKO, (left panel) or reflecting the change in abundance (Log_2_FC) for single deletion of CCPG1 (right panel). **c**, Experimental scheme for multiplexed total proteome analysis of WT, ATG12^−/−^ or TEX264^−/−^/CCPG1^−/−^ iNeurons (n = 3) and violin plots for total ER, ER-lumen, and ER-membrane proteins derived from this analysis. Violin plots from a TMT 18-plex experiment comparing WT, ATG12^−/−^, and another ER-phagy receptor allelic combination (TEX264^−/−^ +CCPG1^−/−^) with complementary comparisons of Log_2_FC (mutant/WT) distributions of ER proteins (top), ER lumenal proteins (middle), or ER membrane proteins (bottom) to distributions of randomized selections of the same number of proteins (100 iterations). p-values for each comparison are calculated with a Kolmogorov–Smirnov Test (two-sided) test. Together these reflect accumulation of the ER, ER lumen and ER membrane for Log2FC (ATG12^−/−^/WT) but only the ER lumenal proteome accumulates for Log_2_FC (TEX264^−/−^ +CCPG1^−/−^ /WT). **d**, ER contact site protein VAPB accumulates with additional ER-phagy receptor knockout. Plot is annotated as in panel **a**.

The ER lumenal compartment is primarily responsible for folding and modification of secretory and membrane proteins, but proteins in this compartment have also been reported to undergo autophagic trafficking^8,40,41^. We identified two major patterns of ER lumenal protein abundance changes, reflected in the *β*^WT→DKO^ and *β*^QKO→PKO^ coefficients. In total, 25 ER lumen proteins (primarily lacking a TM) were stabilized in the DKO mutant, and ten of these were further stabilized in PKO mutants (Fig. 8b and Supplementary Table 3). In contrast, a distinct cohort of 16 ER lumen proteins was stabilized specifically in the PKO mutant, with no significant effect observed with DKO, TKO or QKO mutants (for example, P4HA1 and P4HA2) (Fig. 8a,b, Extended Data Fig. 10a,b and Supplementary Table 3), and the log_2_FC for these lumenal proteins was also stabilized in ATG12^−/−^ iNeurons (Extended Data Fig. 10b). These findings suggest both redundant and specific lumenal cargo for FAM134A/C and CCPG1 receptors. We also found it compelling that deletion of CCPG1 alone or in the context of a TEX264^−/−^/CCPG1^−/−^ double mutant resulted in increased abundance of a subset of lumenal proteins with significant similarities to that seen with the PKO mutant (Extended Data Figs. 8c and 10b,c and Supplementary Table 11).

Intriguingly, the single TM segment proteins VAPA and VAPB, which mediate contact site interactions between ER and a number of other organelles, including mitochondria, via an interaction with VPS13 and other lipid transfer proteins^42^, have a positive *β* coefficient in DKO and/or PKO mutants, indicating that VAPs undergo multiple modes of ER-phagic turnover (Fig. 8a,b,f and Extended Data Fig. 10d). An increase in VAPA abundance was also observed by immunoblotting in both ATG12^−/−^ and PKO iNeurons (Extended Data Fig. 9c). To directly examine the hypothesis that VAPA is an ER-phagy client, we created a Keima-VAPA reporter construct that was expressed via PiggyBac in WT, ATG12^−/−^ and PKO iNeurons. We found that Keima-VAPA autophagic flux was reduced in PKO iNeurons to an extent similar to that seen with ATG12^−/−^ iNeurons, consistent with the idea that VAPA is a substrate for ER-phagy (Fig. 8g). VAPA abundance was also increased in ATG5^−/−^ cerebellar granule neurons in culture^22^. In parallel, *β*-coefficient correlation plots for organelles revealed selective accumulation of mitochondria as a result of CCPG1 deletion (Extended Data Figs. 5c and 8a–c). These findings encourage further study into how ER-phagy mechanisms are regulating the ER architecture to facilitate functions like maintaining robust yet dynamic contact sites with other organelles.

## Discussion

Previous studies have indicated that loss of autophagy pathways in neurons from mice or in iNeurons from hESCs leads to increased accumulation of ER proteins^22,25^, but the extent to which this reflects non-specific macroautophagy or selective ER-phagy was unknown. The use of a genetically tractable iNeuron system, which displays a dramatic accumulation of axonal ER in the absence of a functional autophagy system^25^, has allowed us to examine roles for multiple ER-phagy receptors during ER remodelling associated with neurogenesis.

We found that FAM134C and TEX264 are mobilized into LC3B-positive vesicles that traffic in axons. During nutrient stress, FAM134 and TEX264 are concentrated into the same ER structures that are captured during ER-phagy, whereas CCPG1 forms distinct domains^9^. Current models indicate that ER serves as a source of lipids for phagophore formation, but that ER-membrane proteins themselves are not incorporated into autophagosomal membranes^23,24^. Thus, we conclude that FAM134C- and TEX264-positive puncta reflect ER-phagy rather than the process of autophagosome biogenesis as previously observed in distal axons^34^. Importantly, we observed numerous autophagosomes in axons via cryo-ET, some of which contain ER membranes and TEX264-GFP in correlative imaging. Tomogram reconstruction revealed the presence of membranes consistent with tubular ER (Fig. 5). Mutation of FAM134A and C or FAM134B and C was sufficient to produce a global increase in the ER proteome, with the TM ER proteome featured prominently among the most stabilized proteins. In contrast, deleting CCPG1 in different allelic backgrounds revealed CCPG1’s primary role in clearing lumenal proteins. Unlike FAM134 family members and TEX264, CCPG1 contains a lumenal domain that has been suggested to associate with lumenal autophagy substrates^8,40,43^. Our proteomic analysis validates previously reported CCPG1 cargo (for example, P3H4)^40^ and provides additional candidates for further analysis. Unlike ER-phagy in response to nutrient stress^9,10^, it does not appear that loss of TEX264 alone affects ER network clearance during neurogenesis, and our results suggest that TEX264 ER-phagic clearance is dependent on the FAM134 ER-phagy receptor family in this context.

FAM134 proteins are thought to cluster into highly curved membranes during an early step in ER-phagy initiation, thereby promoting ER-membrane budding and scission of ER membrane into autophagosomes^15,21^. Other RHD-containing proteins, including REEP5 and ARL6P1/5^21,44^, can associate with FAM134C. Although the majority of REEP proteins and several RTN proteins accumulated in ATG12^−/−^ iNeurons, cells lacking FAM134A/C accumulate REEP5 and RTN1 but display loss of REEP1–4. REEP1–4 protein abundance was rescued upon expression of FAM134C-GFP in FAM134A/C^−/−^ cells. One possible explanation for this result is that FAM134 proteins facilitate REEP1–4 trafficking or stability. Unlike REEP5/6, which contain four reticulon helices within the outer leaflet of the ER membrane, REEP1–4 contain only three such helices and therefore may have distinct functional properties. REEP1–4 orthologues bind to highly curved membranes, including small vesicles, in contrast to REEP5/6^45,46^. Future studies are required to understand the distinct properties of REEP proteins observed here and to understand any role of receptor phosphorylation^8,47,48^ in neuronal ER-phagy. This Article provides a versatile resource for further interrogating how ER remodelling is optimized for various cell states via selective ER-phagy.

## Methods

Research within this publication complies with relevant ethical regulations. Commercially sourced H9 hESCs (WiCell) were exempted by Harvard University Embryonic Stem Cell Research Oversight Committee under approval no. E00051 as human subjects research due to patient de-identification.

Protocols associated with this work can be found on protocols.io at 10.17504/protocols.io.81wgbx13nlpk/v3.

### Reagents

The following chemicals, peptides and recombinant proteins were used: DAPI (Thermo Fisher Scientific, D1306); TMTpro 16plex Label Reagent Set (Thermo Fisher Scientific, A44520); Q5 Hot Start High-Fidelity DNA Polymerase (New England BioLabs, M0493); Gateway LR Clonase II Enzyme Mix (Thermo Fisher Scientific, 11791020); NEBuilder HiFi DNA Assembly Master Mix (E2621s); MiSeq Reagent Nano Kit v2 (300 cycles; Illumina, MS-103-1001); bafilomycin A1 (Cayman Chemical, 88899-55-2); Sar405 selective ATP-competitive inhibitor of Vps34 (Apexbio, A8883); DAPI (4′,6-diamidino-2-phenylindole, dihydrochloride; Thermo Fisher Scientific, D1306); 16% paraformaldehyde (EM grade; Electron Microscopy Science, 15710), PhosSTOP (Sigma-Aldrich, T10282); protease inhibitor cocktail (Roche, 4906845001); TCEP (Gold Biotechnology), formic acid (Sigma-Aldrich; 94318); trypsin (Promega, V511C); Lys-C (Wako Chemicals, 129-02541); urea (Sigma, U5378); EPPS (Sigma-Aldrich, E9502); 2-chloroacetamide (Sigma-Aldrich, C0267); Trypan Blue stain (Thermo Fisher Scientific, Wako Chemicals, 129-02541w); urea (Sigma, U5378); EPPS (Sigma-Aldrich, E9502); 2-chloroacetamide (Sigma-Aldrich, C0267); Empore SPE Disks C18 3M (Sigma-Aldrich, 66883-U); GeneArt Precision gRNA synthesis kit (Thermo Fisher Scientific, A29377); 12-well glass-bottom plate with high performance #1.5 cover glass (Cellvis, P12-1.5H-N); Nunc Cell-Culture Nunclon Delta treated 6-well plate (Thermo Fisher Scientific, 140685); Nunc Cell-Culture Nunclon Delta treated 12-well plate (Thermo Fisher Scientific, 150628); 100 × 21-mm dish, Nunclon Delta (Thermo Fisher Scientific, 172931); Corning Matrigel Matrix, growth factor reduced (Corning, 354230); DMEM/F12 (Thermo Fisher Scientific, 11330057); neurobasal (Thermo Fisher Scientific, 21103049); non-essential amino acids (NEAAs, Life Technologies, 11140050); GlutaMAX (Life Technologies, 35050061); N-2 supplement (Thermo Fisher Scientific, 17502048); neurotrophin-3 (NT3) (Peprotech, 450-03); brain-derived neurotrophic factor (BDNF; Peprotech, 450-02); B27 (Thermo Fisher Scientific, 17504001); Y27632 dihydrochloride (ROCK inhibitor; PeproTech, 1293823); Cultrex 3D culture matrix laminin I (R&D Systems, 3446-005-01); accutase (StemCell Technologies, 7920); FGF3 (in-house, N/A); human insulin (Sigma-Aldrich, I9278-5ML); transforming growth factor-β (PeproTech, 100-21C); holo-transferrin human (Sigma-Aldrich, T0665); sodium bicarbonate (Sigma-Aldrich, S5761-500G); sodium selenite (Sigma-Aldrich, S5261-10G); doxycycline (Sigma-Aldrich, D9891); recombinant SpCas9^49^; hygromycin B (Thermo Fisher Scientific, 10687010); UltraPure 0.5 M EDTA, pH 8.0 (Thermo Fisher Scientific, 15575020); GlutaMAX (Thermo Fisher Scientific, 35050061); Dulbecco’s MEM (DMEM), high-glucose pyruvate (GIBCO/Invitrogen, 11995); Lipofectamine 3000 (Invitrogen, L3000008); Click-iT Plus TUNEL assay (Invitrogen, C10617, with Alexa Fluor 488); tunicamycin (Cell Signaling, 12819S); RNAeasy Qiagen kit (Qiagen, 74104); Qiashredder columns (Qiagen, 79654); DNAseI (Thermo, EN0521); oligo dT_20_ primers (Invitrogen, 79654); dNTPs (NEB, N0447L).

### Plasmids

Plasmids constructed for and used in this manuscript will be available at Addgene upon final publication. These include pAC150-Keima-RAMP4 (this Article, Addgene 201929, RRID:Addgene_201929); pAC150-Keima-VAPA (this Article, Addgene 212096, RRID:Addgene_212096); pAC150-Keima-REEP5 (this Article, Addgene 201928, RRID:Addgene_201928); pAC150-FAM134C-GFP (this Article, Addgene 201932, RRID:Addgene_201932); pAC150- TEX264- GFP (this Article, Addgene 201931, RRID:Addgene_201931); pAC150-TEX264(deltaLIR, F273A)-GFP (this Article, Addgene 201930, RRID:Addgene_201930), pHAGE-FAM134C-GFP (this Article, Addgene 201927, RRID:Addgene_201927); pHAGE-TEX264-GFP (Addgene 201925, RRID:Addgene_201925)^9^; pHAGE-TEX264(deltaLIR,F273A)-GFP (Addgene 201926, RRID:Addgene_201926)^9^; pHAGEmCherry-LC3B (Addgene 201924, RRID:Addgene_201924)^9^.

### Cell culture

hESCs (H9, WiCell Institute, WA9, RRID CVCL_9773) or iPSCs (KOLF2.1, Jackson Labs CVCL_D1J6) were cultured in E8 medium on Matrigel-coated plates, as described in ref. ^25^. Cells were split when they reached 80% confluency (every 2–4 days) using 0.5 mM EDTA in 1× Dulbecco’s Phosphate-Buffered Saline (Thermo Fisher Scientific).

### Neural differentiation of AAVS1-TRE3G-NGN2 pluripotent stem cells

TRE3G-NGN2 was integrated into the adeno-associated virus integration site (AAVS) of the hESCs and iPSCs as previously described^50^. To start differentiation to iNeurons from stem cells (day 0), cells were plated at 2 × 10^5^ cells ml^−1^ onto Matrigel-coated plates into ND1 medium (DMEM/F12, 1X N2 (Thermo Fisher Scientific), human BDNF (10 ng ml^−1^; PeproTech), human neurotrophin-3 (NT3, 10 ng ml^−1^; PeproTech), 1X NEAA, human laminin (0.2 μg ml^−1^) and doxycycline (2 mg ml^−1^) also containing Y27632 (ROCK inhibitor, 10 mM). The medium was replaced with ND1 without Y27632 the next day. The following day, the medium was replaced with ND2 (neurobasal medium, 1X B27, 1X GlutaMAX, BDNF (10 ng ml^−1^), NT3 (10 ng ml^−1^) and doxycycline at 2 mg ml^−1^. On days 4 and 6, 50% of the medium was changed with fresh ND2. On any day in the day 4–7 range, cells were replated at 4 × 10^5^ cells well^−1^ in ND2 medium with Y27632. The medium was replaced the next day with fresh ND2 (without Y27632). Every other day, 50% of the medium was changed with ND2. At day 9 and onwards, doxycycline was removed from the ND2 mixture. iNeurons were fed every other day with 50% medium change until the experimental day (day 12 of differentiation, unless otherwise noted).

### Molecular cloning

Plasmids were made using either Gateway technology (Thermo Fisher Scientific) or Gibson assembly (New England Biolabs) in the pHAGE backbone (for lentivirus transduction) or in the pAC150 piggyBac backbone (for stable hESC generation). Entry clones from the human ORFeome collection, version 8, were obtained and cloned via in vitro recombination between each entry clone which contains the gene of interest flanked by attL sites and to various destination vectors containing attR sites (LR cloning).

### Viral transduction of iNeurons

Lentiviral vectors were packaged in the HEK293T cell line (ATCC, CRL-1573, RRID: CVCL_0045) by co-transfection of pPAX2 (Addgene 12259, RRID:Addgene_12259), pMD2 (Addgene 12260, RRID:Addgene_12260) and the vector of interest in a 4:2:1 ratio using Lipofectamine 3000. One day after transfection, the medium was changed to ND2 (no doxycycline), then, the following day, virus containing supernatant was collected, filtered through a 0.22-μm syringe filter and frozen at −80 °C. hESCs or iPSCs were differentiated to neurons as described above. At day 11 (two days after doxycycline removal) the iNeurons were transduced. iNeurons were imaged one day after transduction or on any following day (the experimental day is noted in each figure).

### Stable hESC population generation

The piggyBac plasmids freshly Maxiprepped at high concentrations were electroporated into hESCs using the 10-μl Neon Thermo Fisher kit and Thermo Fisher Neon Electroporator, then 1.5 μg of pAC150 piggyBac vectors for ER proteins (Keima-RAMP4, TEX264-GFP, FAM134C-GFP and 1 μg of pCMV-hyPBase hyperactive piggyBac vector). Next, 2 × 10^5^ cells in 10 μl of buffer R were used for each electroporation. Program 13 was used from the optimization tab for the electroporation parameters (voltage, 1,100; pulse width, 20; pulse number, 2). We plated the electroporated ESCs into Matrigel-coated plates containing E8 with Y27632 (ROCK inhibitor, 10 mM) and the cells were placed in a low-O_2_ incubator for two to four days. After four days with regular E8 medium changes daily (or when the cells reached 80% confluency), the cells were split into selection medium (E8 with Y27632 and 50 μg ml^−1^ hygromycin B). The cells were grown in the selection medium for seven to ten days until there was no longer any cell death, then the cells were further selected to obtain a fluorophore-positive population via flow cytometry with a Sony Biotechnology (SH800S) cell sorter.

### Gene editing

Gene editing in the hESCs was performed as in ref. ^51^. Guide RNAs (sgRNAs) were generated using the GeneArt Precision gRNA synthesis kit (Thermo Fisher Scientific), then 0.6 μg of sgRNA was incubated with 3 μg of SpCas9 protein for 10 min at room temperature and electroporated into 2 × 10^5^ H9 cells using a Neon transfection system (Thermo Fisher Scientific). The cells were placed in a low-O_2_ incubator and allowed to recover for 24–72 h, then they were single-cell-sorted into 96-well plates with the Sony Biotechnology (SH800S) cell sorter and grown up for 7–12 days. Individual clones were verified for out-of-frame deletions by DNA-sequencing with an Illumina MiSeq system, and protein deletion was verified by immunoblotting. The sgRNA target sequences were as follows: CCPG1 sgRNA TTCTAACTTAGGTGGCTCAA, TEX264 sgRNA CATGTCGGACCTGCTACTAC, FAM134A sgRNA TGCATCACAAACACGACAAGAGG, FAM134B sgRNA GTCTGACACAGACGTCTCAG, FAM134C sgRNA CTCTCATTGTCTAATGCGTC and sgRNA CALCOCO1 TGTGGTCTTCCGTGCCTGAAAGTA. The cell lines reported here are available upon request, but transfer requires that the recipient have a licence for H9 ESCs from WiCell.

### Antibodies

The following antibodies were used: ATG5 rabbit monoclonal (D5F5U) antibody (Cell Signaling Technology 12994S, lot 5, western blot (WB) 1:1,000, RRID: AB_2630393); FAM134B rabbit polyclonal antibody (Proteintech 21537-1-AP, lot 00100765, WB 1:1,000 RRID: AB_2878879); FAM134C rabbit polyclonal antibody (Sigma-Aldrich HPA016492, lot R06641, WB 1:1,000, RRID: AB_1853027); CCPG1 rabbit monoclonal (E3C5G) antibody (Cell Signaling Technology 80158, lot 1, WB 1:1,000, RRID: AB_2935809); TEX264 rabbit polyclonal antibody (Sigma-Aldrich HPA017739, lot 000012723, WB 1:1,000, RRID :AB_1857910); REEP1 rabbit polyclonal antibody (Sigma-Aldrich HPA058061, lot R81573, WB 1:1,000, RRID: AB_2683591); REEP4 rabbit polyclonal antibody (Sigma-Aldrich HPA042683, lot R39936, WB 1:1,000, RRID: AB_2571730); REEP5 rabbit polyclonal antibody (Proteintech 14643-1-AP, lot 00050540, WB 1:1,000, RRID: AB_2178440); RTN3 mouse monoclonal (F-6) antibody (Santa Cruz sc-374599, lot 10922, WB 1:1,000, RRID: AB_10986405); CKAP4/p63 sheep polyclonal antibody (RD Biosciences AF7355, lot CGDGG012105B, WB 1:1,000, RRID: AB_10972125); CKAP4 rabbit polyclonal antibody (Proteintech 16686-1-AP, lot 0052093, WB 1:1,000, RRID: AB_2276275); hFAB rhodamine anti-tubulin antibody (BioRad 12004166, lot 64512247, WB 1:10,000, RRID: AB_2884950); HSP90 mouse monoclonal (4F10) antibody (Santa Cruz sc-69703, lot J2721, WB 1:10,000, RRID: AB_2121191); GAPDH XP rabbit monoclonal (D16H11) antibody (Cell Signaling Technology 5174, lot 8, WB 1:1,000, RRID: AB_10622025); CREB-2/ATF4 mouse monoclonal (B-3) antibody (Santa Cruz, sc-390063, lot J2021, WB 1:1,000, RRID: AB_2810998); VAPA rabbit monoclonal (EPR13589(B)) antibody (Abcam ab181067, lot GR164232-2, WB 1:1,000, RRID: AB_3073850); RTN1 (isoform RTN1-C) rabbit polyclonal antibody (Proteintech 15048-1-AP, lot 00043268, WB 1:1,000, RRID: AB_2185981); goat anti-rabbit immunoglobulin-G (IgG) horse radish peroxidase (HRP) conjugate (BioRad 1706515, lot 64559210, WB 1:3,000, RRID: AB_11125142); goat anti-mouse IgG HRP conjugate (BioRad 1706516, lot 64526160; WB 1:3,000, RRID: AB_11125547); neurofilament heavy polypeptide mouse monoclonal (NF-01) antibody (Abcam ab7795, lot GR3448163-1, IF 1:300, RRID: AB_306084); MAP2 guinea pig polyclonal antibody (Synaptic Systems 188004, lot 6-49, IF 1:300, RRID: AB_2138181); Nogo-A (RTN4) mouse monoclonal (C-4) antibody (Santa Cruz sc-271878, lot D2420, IF 1:300, RRID: AB_10709573); calnexin rabbit polyclonal antibody (Proteintech 10427-2-AP, lot 00094417, IF 1:300, RRID: AB_2069033); goat anti-mouse Alexa488 (Thermo Fisher Scientific A-11001, lot 2379467, IF 1:300, RRID: AB_2534069); goat anti-chicken Alexa488 (Thermo Fisher Scientific A11039, lot 218068, IF 1:300, RRID: AB_2534096); goat anti-rabbit Alexa568 (Thermo Fisher Scientific A-11011, lot 2500544, IF 1:300, RRID: AB_143157); goat anti-rabbit Alexa647 (Thermo Fisher Scientific A27040, lot 2659317, IF 1:300, RRID: AB_2536101); goat anti-guinea pig Alexa488 (Thermo Fisher Scientific A-11073, lot 38320A, IF 1:300, RRID: AB_2534117); goat anti-guinea pig Alexa647 (Thermo Fisher Scientific A-21450, lot 2446026, IF 1:300, RRID: AB_141882).

### Western blotting

Cell pellets were resuspended in 8 M urea buffer (8 M urea, 150 mM Tris pH, 150 mM NaCl) supplemented with protease and phosphatase inhibitor tablets and then sonicated twice, 10 s each, on ice. The lysates were clarified via centrifugation at 20,000*g* for 10 min at 4 °C. bicinchoninic acid (BCA) assays were performed on clarified lysates, and normalized lysate amounts were boiled in 1X SDS containing Laemmeli buffer. Lysates were run on 4–20% Tris glycine gels (BioRad) and transferred via wet transfer onto polyvinylidene difluoride membranes for immunoblotting with the indicated antibodies. Images of blots were acquired using enhanced chemiluminescence or using the rhodamine channel on a BioRad ChemiDoc imager, and the images were quantified and converted to jpeg for publication using BioRad Image Lab Software v5.2.5 RRID:SCR_014210.

### Flow cytometry

hESCs that were converting to neurons were grown in six-well plates and treated with various drugs for the indicated time points, then cell pellets were collected at the indicated day of neuronal differentiation. These were resuspended in FACS buffer (1X PBS, 2% FBS). At least 10,000 cells were analysed on an Attune NxT flow cytometer (Thermo Fisher Scientific, cat. no. A28993). The neutral Keima signal was measured at an excitation of 445 nm and emission of 603 nm with 48-nm bandpass, and the acidic Keima signal was measured at an excitation of 561 nm and emission of 620 nm with 15-nm bandpass. The resulting cell-population Keima ratio was analysed as previously described^52^. In brief, FCS files were exported into FlowJo (Version 10.5.2, RRID:SCR_008520, https://www.flowjo.com/solutions/flowjo), where the cells were gated for live cells, single cells and Keima-positive cells. The 561 nm (acidic) to 445 nm (neutral) excitation ratio was calculated by dividing the mean values of the 561-nm excited cells by the mean values of the 445-nm excited cells.

### Imaging

Cells were plated onto 6-well, 12-well or 24-well glass-bottom plates with high-performance #1.5 cover glass (CellVis). Live cells were imaged at 37 °C at 5% CO_2_. For the immunofluorescence experiments, cells were fixed at room temperature with 4% paraformaldehyde in PBS, solubilized in 0.1% Triton-X in PBS and blocked with 1% BSA/0.1% Triton-X in PBS. Cell were then immunostained with anti-primary antibodies used at 1:500 and then Alexa Fluor-conjugated antibodies (Thermo Fisher) used at 1:300. The primary and secondary antibodies used in this study are identified in the Antibodies section above and described for each experiment detailed in the following. Fixed cell images were captured at room temperature. Cells were imaged using a Yokogawa CSU-X1 spinning disk confocal unit on a Nikon Ti-E inverted microscope at the Nikon Imaging Center in Harvard Medical School. The Nikon Perfect Focus System was used to maintain cell focus over time. The microscope was equipped with a Nikon Plan Apo ×40/1.30 NA or ×100/1.40 NA objective lens and 445-nm (75 mW), 488-nm (100 mW), 561-nm (100 mW) and 642-nm (100 mW) laser lines controlled by Acousto-Optic Tunable Filter system. All images were collected with a Hamamatsu ORCA-Fusion BT sCMOS camera (6.45-µm^2^ photodiode) with Nikon Elements (version AR, RRID:SCR_014329) image acquisition software.

#### Analysis of ER structures in axons

hESC-derived iNeurons were imaged at the indicated day in neuronal differentiation. Cells were fixed and stained as described above with α-calnexin to detect ER, α-MAP2 to detect dendrites, α-NEFH to mark axons, and DAPI to detect nuclei. The *z* stacks were acquired with the parameters stated above in the Imaging section, and the *z* series are displayed as maximum *z* projections, with the brightness and contrast adjusted for each image equally and then converted to rgb for publication using Fiji software (Version 2.0.0, RRID:SCR_014329, http://fiji.sc). Fiji software was also used to split the *z* projections into individual channels for downstream image analysis in CellProfiler Image Analysis Software (Version 4.2.5, RRID:SCR_007358, http://cellprofiler.org)^53^. Each field of view for all genetic backgrounds was thresholded in the same way with a consistent pipeline. The ‘identify primary objects’ tool was used to find nuclei, axons, dendrites and ER structures. The α-NEFH-positive axon object regions were used to create an axon mask, and ER structures within this mask were counted. The area of each ER structure was also measured. The number of ER axonal structures was then compared to the number of detected nuclei.

#### Analysis of cell nuclei using size

We assayed whether nuclei were intact in the images used to assess the amount and size of ER structures in the axons, as described above in the Imaging section (α-calnexin to detect ER, α-MAP2 to detect dendrites, α-NEFH to mark axons, DAPI to detect nuclei, and with *z* projections already split into individual channels as detailed above for downstream image analysis in CellProfiler^53^). The DAPI channel images for all genetic backgrounds were thresholded in the same way with the following pipeline. Two different ‘identify primary objects’ modules were used to find and count nuclei structures. In one, only larger ‘intact’ nuclei were selected and counted (as was done previously for the analysis of ER structures in axons to determine ER structures per nuclei). In the second, smaller fragmented nuclei were included in the thresholding method. The ratio of intact to total DAPI-positive nuclei structures was calculated and reported for each condition.

#### Analysis of cell nuclei using TUNEL

As secondary confirmation that the intact nuclei that we were assaying were indeed healthy, we performed a Click-iT Plus TUNEL (terminal deoxynucleotidyl transferase dUTP nick end labelling) assay (Invitrogen, C10617, Alexa Fluor 488), which detects DNA breaks formed when DNA fragmentation occurs at the end of apoptosis. We prepared four new differentiations of WT, ATG12 and PKO neurons (hESC-derived) at day 20 to perform this staining. In short, following the kit protocol, after fixing and permeabilizing the iNeurons as already described, we followed the kit directions to first perform a TdT reaction. In this reaction, the TdT enzyme takes EdUTP (a dUTP modified with a small, bio-orthogonal alkyne moiety) and incorporates it at the 3′-OH ends of fragmented DNA. Next, we performed the click reaction, a copper-catalysed covalent reaction occurring between the Alexa Fluor picolyl azide dye and an alkyne. Detection of the DNA break is based on the Alexa Fluor signal at that site. After performing this Click-iT Plus TUNEL reaction, we next stained with DAPI to label all DNA structures (this labels both intact and fragmented DNA). The *z* stacks were acquired with the parameters stated above. The *z* series are displayed as maximum *z* projections and brightness, and the contrast was adjusted for each image equally and then converted to rgb for publication using Fiji software. Fiji software was also used to split the *z* projections into individual channels for downstream image analysis in CellProfiler^53^. For the TUNEL channel images, images from all genetic backgrounds were thresholded in the same way using an ‘identify primary objects’ module to find and count all damaged DNA structures, including larger and smaller structures. For the DAPI channel images, two different ‘identify primary objects’ modules were used to find and count the DAPI structures. In one, only larger ‘intact’ DAPI-positive nuclei were selected. In the second, smaller fragmented DAPI-positive nuclei were included in the thresholding method. To calculate the total nuclei number, the number of damaged TUNEL-positive DNA structures was added to the number of intact DAPI nuclei. In the final analysis, the ratio of intact DAPI-positive nuclei structures to total nuclei (damaged TUNEL-positive nuclei plus intact) was calculated and reported for each condition.

#### Visualizing Keima-ER in neuronal differentiation

Live cells (hESC-derived) stably expressing Keima-RAMP4 (localizes to all ER) or Keima-REEP5 (localizes to ER tubules specifically) were imaged at the indicated day in neuronal differentiation. Pairs of images for ratiometric imaging of Keima-RAMP4 fluorescence were collected sequentially using 100-mW 442-nm (neutral Keima excitation) and 100-mW 561-nm (acidic Keima excitation) solid-state lasers, and the emission was collected with a 620/60-nm filter (Chroma Technologies). The *z* stacks were acquired with a Nikon Plan Apo ×40/1.45-NA oil-objective lens. The *z* series are displayed as maximum *z* projections, and the brightness and contrast were adjusted for each image equally and then converted to rgb for publication using Fiji software. Fiji software was also used to split the *z* projections into individual channels. For each channel, complementary line scans, 30 μm long and 1.7 μm wide, were drawn in either the soma or projection of the iNeurons. The 561-nm or 442-nm grey values along these lines were measured using ‘plot profile’ in Fiji. The 561/442 ratio of these values at each complementary point along the line was calculated and plotted in Microsoft Excel (Version 16.81, RRID:SCR_016137, https://www.microsoft.com/en-gb/).

#### Characterizing the spatial and temporal properties of ER-phagy receptors

hESCs with WT or ATG12^−/−^ genetic background stably expressing WT or mutant TEX264-GFP or FAM134C-GFP were converted to neurons and treated with various drugs for the indicated time points, and imaged at the indicated day in neuronal differentiation. The *z* stacks were acquired with the parameters stated above. The *z* series are displayed as maximum *z* projections and brightness and contrast were adjusted for each image equally and then converted to rgb for publication using Fiji software.

For day-4 cells (untreated or treated with the indicated drugs), the number of GFP puncta per cell was quantified using CellProfiler. Each field of view for all genetic backgrounds and drug treatments was thresholded in the same way with a consistent pipeline. Using the ER-phagy receptor (488 nm excitation, GFP channel) max *z*-projection image, the ‘identify primary objects’ tool was used to detect cells (the receptor labels the whole ER membrane, which can be used to identify cells) and to detect puncta (small bright circles found within the ER membrane). The puncta were linked to each cell, and the puncta per cell number were exported.

Autophagosome (LC3B) and ER-phagy receptor (TEX264 or FAM134C) co-labelling was achieved by transducing with mCh-LC3B and receptor-GFP lentivirus. Day-30 neurons were imaged live for 30 min with an image acquired every 30 s. Fiji was used to track GFP- and mCh-positive puncta. Lines between each frame were used to measure the distance travelled by the puncta from frame to frame. The forward direction is reported as a positive value in micrometres and the backward direction as a negative value. Events in neurons from three independent differentiations were captured. The events were binned based on their speed of movement in units of micrometres per second. The percentage of events at each speed was plotted as using GraphPad Prism (9.5.0).

After live-cell imaging at day 30, the ER-phagy receptor and mch-LC3B-positive transduced neurons were fixed as described above. The iNeurons were immunostained with α-MAP2 to detect dendrites and α-NEFH to mark axons. The *z* stacks were acquired with the parameters stated above. The *z* series are displayed as maximum *z* projections, and the brightness and contrast were adjusted for each image equally and then converted to rgb for publication using Fiji software.

### RNA extraction, RT–PCR, DNA gel electrophoresis

At day 12, iNeurons (hESC-derived) of each genotype were left untreated or treated with tunicamycin. After 4 h, all the cells were scraped off the dishes, pelleted and washed three times with PBS. The number of cells was determined and the pellets were snap-frozen in liquid nitrogen and stored at −80 °C for a few days before use. The cell pellets were thawed and resuspended in freshly prepared RNeasy Lysis Buffer (350 µl per sample for 1 × 10^6^ cells) from the RNAeasy Qiagen kit (Qiagen, 74104). Dnase1 digestion buffer was then added, and the cells were lysed by passage through a Qiashredder column (Qiagen, 79654). One volume of 70% ethanol was added to the lysate, and the lysate–EtOH solution was transferred to an RNAeasy spin column. The following spins including on column DNAseI (Thermo Fisher Scientific, EN0521) digestion, buffer washes and RNA elution, were performed following the RNAeasy Qiagen kit directions. The concentration of final extracted RNA for each condition was measured using a NanoDrop spectrophotometer. Reverse transcription reactions for each condition (using the same amount of starting micrograms of RNA, 0.5 µg, in each reaction) were performed with Superscript III reverse transcriptase master mix (Invitrogen, 18080-051) using oligo dT_20_ primers (Invitrogen, 79654) and dNTPs (NEB, N0447L) to create complementary DNA (cDNA). With the cDNA, PCR reactions were performed to amplify cDNA from GAPDH mRNA (forward 5′-GGATGATGTTCTGGAGAGCC-3′; reverse 5′-CATCACCATCTTCCAGGAGC-3′) or to amplify cDNA from unspliced XBP1 mRNA or spliced XBP1 mRNA (forward 5′-CCTTGTAGTTGAGAACCAGG-3′; reverse 5′-GGGGCTTGGTATATATGTGG-3′) (as performed in refs. ^54,55^). The PCR products were electrophoresed on a 2.5% agarose gel. The size difference between the spliced and unspliced XBP1 was 26 nucleotides.

### Transmission electron microscopy

#### Cell preparation

iNeurons were grown on Aclar plastic discs in 12-well plates coated with Matrigel. At day 20, the iNeurons were fixed with 2.5% glutaraldehyde, 1.25% paraformaldehyde and 0.03% picric acid in 0.1 M sodium cacodylate buffer (pH 7.4). A 2× solution was diluted 1:1 with the cell medium in the dish. Cells were fixed at room temperature for 1 h.

#### Epon embedding

The following steps were performed by the Harvard Medical School Electron Microscopy Core: cells were washed in 0.1 M sodium cacodylate buffer (pH 7.4), post-fixed for 30 min in 1% osmium tetroxide (OsO_4_)/1.5% potassium ferrocyanide (KFeCN_6_), washed twice in water and once in maleate buffer and incubated in 1% uranyl acetate in maleate buffer for 30 min followed by two washes in water and subsequent dehydration in grades of alcohol (5 min each; 50%, 70%, 95%, 2 × 100%). The samples were subsequently embedded in TAAB Epon (TAAB Laboratories Equipment, https://taab.co.uk) and polymerized at 60 °C for 48 h. Note on embedding—a drop of Epon was placed onto a clean piece of Aclar, then the coverslip was removed from 100% EtOH with a pair of fine-tipped forceps. Excess EtOH was removed by quickly blotting the side of the coverslip onto filter paper (to make sure the cells do not dry out) and the coverslips were placed (cell side down) onto the Epon drop. A small weight on top helped with keeping it flat. After polymerization, the Aclar was peeled off, a small area (~1 mm) of the flat embedded cells was cut out with a razor blade and remounted on an Epon block. Ultrathin sections (~80 nm) were cut on a Reichert Ultracut-S microtome, picked up onto copper grids stained with lead citrate and examined in a TecnaiG² Spirit BioTWIN and images recorded with an AMT 2k charge-coupled device camera. Regions close to the coverslip were specifically targeted to capture neuronal processes, not somata.

### Cryo-electron tomography

#### Cryo-ET sample preparation and freezing

AAVS-TREG3-NGN2 non-embryonic and internationally accepted iPSCs were differentiated to iNeurons (KOLF2.1) and cultured on EM grids as described in detail in the following protocol: https://www.protocols.io/view/neural-differentiation-on-em-grids-ineurons-sample-5jyl8jz36g2w/v2. In this particular case, a 200-mesh gold grid with silicon dioxide R2/1 film (Quantifoil) was coated with Matrigel as reported in the protocol. The iPSC-derived iNeurons on the grid were transduced at day 12 with lentiviruses carrying mCherry-LC3B and TEX264-GFP. In the following days, the medium was gradually and completely exchanged with ND2 without phenol red (prepared using phenol red-free Neurobasal; Thermo Fisher, Gibco 12348017). The iNeurons were plunge frozen on DIV 18 using a Vitrobot Mark IV unit (Thermo Fisher Scientific) with application of 4 μl of phenol red-free ND2 medium and with the following settings: room temperature, humidifier 70%, Blot-Force 8, Blot-Time 9 s.

#### Cryo-fluorescence data acquisition and processing

Fluorescence stacks of the grid squares of interest were acquired before tilt-series acquisition on an SP8 cryo-confocal laser scanning microscope equipped with a cryo stage and a ×50/0.9-NA objective (Leica). Stacks were acquired sequentially in the red (excitation, 552 nm/emission, 598–625 nm) and green (excitation, 488 nm/emission, 498–525 nm) channels using hybrid detectors, with an *x*/*y* pixel size of 60 nm and a *z* step size of 400 nm. Transmitted light data were collected simultaneously to visualize the positions of the support film holes for 2D registration and correlation. Fluorescence data were deconvolved with Huygens (SVI) (Version 21.10.0p0, RRID:SCR_014237, https://svi.nl/HuygensSoftware) using a theoretical PSF and CMLE method. Maximum intensity projections (MIPs) of the fluorescence channel stacks were generated in Fiji^56^. For transmitted light data, one slice focused on the holes of the support film was selected and then used for the 2D-correlation procedure.

#### Cryo-electron tomography data acquisition and processing

TEM data acquisition was performed on a Krios G4 microscope at 300 kV with a Selectris X energy filter and Falcon 4i camera (Thermo Fisher Scientific) using Tomo5 (version 5.12.0, Thermo Fisher Scientific, RRID:SCR_021359, https://tomopy.readthedocs.io/). Tilt series were acquired at a nominal magnification of ×42,000 (pixel size, 2.93 Å) using a dose-symmetric tilt scheme with an angular increment of 2°, a dose of 2 e^−^ Å^−2^ per tilt and a target defocus between −2.5 and −4 µm. Tilt series were collected from −60° to 60° with a total dose of 120 e^−^ Å^−2^, and frames were saved in the EER file format. The positions for tilt-series acquisition were determined by visual inspection of ×11,500 magnification ‘search’ montage maps acquired in thin areas of the sample (Extended Data Fig. 3a). Tilt series were recorded where double-membrane vesicle structures could be seen inside intact iNeuron projections, with continuous plasma membrane and microtubules bundles. Most of the autophagosomes were captured in areas in which the sample was thicker than 400 nm (Extended Data Fig. 3o). Although such a high sample thickness is generally not recommended for subtomogram averaging of particles because of the low signal-to-noise ratio (SNR) of the resulting images, it still allowed neural-network-based segmentation and visualization of autophagosomes and their membrane cargo. The tilt-series frames were motion-corrected with Relion’s implementation of Motioncorr2 (Version 4.0, RRID:SCR_016499, https://emcore.ucsf.edu/cryoem-software) for EER files^57^, and reconstruction was performed in IMOD (v.4.10.49, RRID: SCR_003297, https://bio3d.colorado.edu/imod/) using the TomoMAN wrapper scripts 10.5281/ZENODO.4110737. Tomograms at 2× binning (IMOD bin 4) with a nominal pixel size of 1.172 nm were denoised using cryo-CARE^58^ (https://github.com/juglab/cryoCARE_T2T).

#### Cryo-electron tomography dataset annotation and analysis

All double-membrane compartments were relatively tight and had regular intermembrane spacing and near-spherical shapes were identified as autophagosomes. Note that although some autophagosomes presented a tight and homogeneous intermembrane distance (Extended Data Fig. 3n, autophagosomes 3 and 4), others presented variable intermembrane distances, or small bumps in the inner membrane (Fig. 5f), which might suggest that they are amphisomes—autophagosomes that have already fused with one or a few small lysosomes^59^. Thirty-two tomograms contained autophagosomes, and some of them contained more than one, leading to total count of 37 autophagosomes. All structures were first annotated by cargo. Tubular membrane cargo structures, morphologically similar to the ER tubules present in the cytoplasm of the same tomograms (Fig. 5c and Extended Data Fig. 3e), were labelled as ‘tubular ER cargo’. Note that the autophagosomes often contained single-membrane vesicle cargo next to the tubular ER (Fig. 5c,f and Extended Data Fig. 3n). Second, autophagosomes were annotated as ‘microtubule-linked’ when microtubules were found at a distance closer than 20 nm from their outer membrane (Extended Data Fig. 3f). Tomogram thickness was determined in 3Dmod (IMOD) by measuring the distance between the upper and lower boundaries of the sample, where small pieces of ice contamination are often visible (Extended Data Fig. 3o). Plots were generated with Python Programming Language (Version 3.9.7, RRID:SCR_008394, https://www.python.org/downloads/release/python-360/) using pandas 1.3.0 (https://pandas.pydata.org/, RRID: SCR_018214)^60^, Matplotlib 3.3.0 (https://matplotlib.org/,RRID:SCR_008624)^61^ and seaborn 0.11.0 (https://seaborn.pydata.org/, RRID: SCR_018132)^62^ packages.

#### 2D correlation of cryo-fluorescence data on TEM images

Correlation of all autophagosomes with the previously acquired cryo-fluorescence data was investigated through a two-step procedure using Fiji’s BigWarp plugin (Version 9.0.0, https://github.com/saalfeldlab/bigwarp)^63^ as follows. First, the centres of different holes on the support film were selected both in a ×800 TEM ‘overview’ image and in a transmitted light image previously acquired on the cryo-confocal microscope (Extended Data Fig. 3g). After registration, the ×800 TEM image was transformed (affine transformation) and overlaid on the green and red fluorescence MIPs (Extended Data Fig. 3h). The overlay was then cropped to obtain a subregion around the tomogram position, which was used for the second correlation with the ×11,500 TEM ‘search’ image (Extended Data Fig. 3i). This time, fine landmarks such as pieces of ice, features of the cellular sample and positions along the hole were selected and used for transforming the cropped fluorescence data (affine transformation). A final overlay of the ×11,500 TEM search image with the cryo-fluorescence data was generated to visualize the correlation of the autophagosomes with the fluorescence signals (Extended Data Fig. 3k,m). This procedure yielded a total of *n* = 5 out of 37 autophagosomes coinciding with distinct TEX264-GFP signal peaks (Fig. 5b). All five tomograms coinciding with TEX264 contained tubular ER cargo, and four of them were in close proximity to microtubules (Extended Data Fig. 3l). Note that the LC3B cryo-fluorescence signal often appears diffuse and bright in the cytosol, and it was difficult to distinguish peaks of signal corresponding to the autophagic structures (Extended Data Fig. 3k,m). This might be due to LC3B also localizing on microtubules or being distributed throughout the cytosol and its involvement in other cellular processes, such as non-canonical autophagy^33^ and LC3-associated phagocytosis^64^. Consequently, the LC3B-mCherry signal could not be used for reliable cryo-correlation in this case. Moreover, extracellular debris, pieces of ice and other intracellular structures can often be autofluorescent at cryogenic temperatures in the green and/or red channels. This phenomenon has been reported previously by others and increases the noise in cryo-fluorescence images^65^.

#### Tomogram segmentation

Segmentations were carried out for the five tomograms of autophagosomes coinciding with the TEX264 signal. The membranes were segmented with Membrain-Seg (https://github.com/teamtomo/membrain-seg), a U-Net based tool for membrane segmentation in cryo-ET data, using the publicly available ‘best’ pretrained model (v9). This method reliably detects membranes, even in very thick tomograms (Extended Data Fig. 3n,o). However, it often merges membranes corresponding to different compartments and sometimes picks cytoskeletal components such as microtubules and neurofilaments in the very crowded neuronal subcellular environment. To separate the different membrane compartments, a watershed segmentation was performed on the original Membrain-seg output, using as seeds a ‘one-click’ rough segmentation of different membranes generated in Amira (Thermo Fisher Scientific) from the output of TomoSegMemTV^66^. For thicker tomograms, such as autophagosome 3 and 4 (Extended Data Fig. 3n,o), automated segmentations of the cargo ER were manually refined in Amira (Version 2021.2, https://www.thermofisher.com/id/en/home/electron-microscopy/products/software-em-3d-vis/amira-software.html). For autophagosomes 1 and 2 (Fig. 5c,f), microtubules were segmented automatically with Dragonfly (version 2022.2, Comet Technologies Canada, https://www.theobjects.com/dragonfly/index.html). A custom model was trained for each tomogram, following a previously described protocol^67^. 3D renderings of the segmentations were generated in UCSF ChimeraX (Version 1.6.1, RRID:SCR_015872, https://www.cgl.ucsf.edu/chimerax/)^68^.

### Quantitative proteomics

#### Sample preparation for mass spectrometry

Cell pellets were resuspended in 8 M urea buffer (8 M urea, 150 mM Tris pH, 150 mM NaCl) supplemented with protease and phosphatase inhibitor tablets and then sonicated twice, 10 s each, on ice. The lysates were clarified by centrifugation at 20,000*g* for 10 min at 4 °C. BCA assays were performed on clarified lysates, then 100 μg of each sample was taken and the total volume increased to 100 μl total. The samples were reduced using TCEP (0.5 M for 30 min at room temperature) and alkylated (with chloroacetamide, 20 mM for 20 min at room temperature) before methanol-chloroform precipitation with 3:1 methanol, 1:1 chloroform and 2.5:1 water added. The aqueous and organic phases were separated by centrifugation for 5 min at 14,000*g*. Liquid around the protein layer was removed and this protein layer was washed with 1 ml of methanol and then pelleted for 5 min at 14,000*g*. The supernatant was removed. The pellets were then resuspended in 50 μl, 200 mM 4-(2-Hydroxyethyl)-1-piperazinepropanesulfonic acid, 4-(2-Hydroxyethyl)piperazine-1-propanesulfonic acid, N-(2-Hydroxyethyl)piperazine-N′-(3-propanesulfonic acid) (EPPS), pH 8.5. Peptide digestion was carried out using LysC (1:100) for 2 h at 37 °C, followed by trypsin (1:100) overnight, then 25 μl of the digested peptides were labelled by adding 5 μl 100% acetonitrile (ACN) and 7 μl of TMT reagent (20 mg ml^−1^ stock in ACN) for 2 h, and the reaction was quenched using hydroxylamine at a final concentration of 0.5% (wt/vol) for 20 min.

#### Basic pH reversed-phase HPLC

Samples were combined 1:1 such that each channel consisted of the same amount of peptide. The pooled peptide sample was desalted with a 100-mg Sep-Pak solid phase extraction column and then fractionated with basic pH reversed-phase (BPRP) HPLC. Fractionation was executed using an Agilent 1200 pump with an Agilent 300 Extend C18 column (3.5-μm particles, inner diameter of 2.1 mm and length of 250 mm). A 50-min linear gradient from 5% to 35% ACN in 10 mM ammonium bicarbonate pH 8 at a column flow rate of 0.25 ml min^−1^ was used for peptide fractionation. A total of 96 fractions were collected and then concatenated down to 24 superfractions, as described previously^69^. These 24 superfractions were divided into two sets of 12 non-adjacent superfractions and were acidified by adding formic acid to a concentration of 1%. One set of fractions (*n* = 12) were vacuum-centrifuged to near dryness, and each was desalted via StageTip, dried by vacuum centrifugation, and reconstituted in 5% acetonitrile, 5% formic acid before LC-MS/MS analysis.

#### Mass spectrometry data acquisition and processing

Mass spectrometric data were collected on an Orbitrap Fusion Lumos mass spectrometer coupled to a Proxeon NanoLC-1200 UHPLC and a FAIMSpro interface^70^. The 100-μm capillary column was pulled in-lab and packed with 35 cm of Accucore 150 resin (2.6 μm, 150 Å; Thermo Fisher Scientific). Peptides were eluted over a gradient (90 or 110 min) consisting of 5% ACN to 30% ACN in 0.125% formic acid. The scan sequence began with an MS1 spectrum (Orbitrap analysis, resolution 60,000, scan range 350–1,350 or 400–1,600 Th, automatic gain control (AGC) target set as ‘standard’, maximum injection time set to auto). SPS-MS3 analysis was used to reduce ion interference^71,72^. MS2 analysis consisted of collision-induced dissociation (CID) and quadrupole ion trap analysis (AGC 2 × 10^4^, normalized collision energy (NCE) 35, *q*-value 0.25, maximum injection time 35 ms, isolation window 0.7 Th). Following the acquisition of each MS2 spectrum, we collected an MS3 spectrum in which multiple MS2 fragment ions were captured in the MS3 precursor population using an isolation waveform with multiple frequency notches. MS3 precursors were fragmented by higher-energy collisional dissociation (HCD) and analysed using the Orbitrap (NCE 55, AGC 1.5 × 10^5^, maximum injection time 150 ms, resolution 50,000). We used the Real Time Search (RTS) using Orbiter^73^ with a *Homo sapiens* database (UniProt, downloaded August 2020) and we limited MS3 scans to two peptides per protein per fraction. A total of 24 RAW files were collected, with data for 12 non-adjacent superfractions acquired using a compensation voltage (CV) set of −40/−60/−80 V with a 1.25-s TopSpeed cycle used for each CV.

Spectra were converted to mzXML via MSconvert (Version 3.0, https://bio.tools/msconvert)^74^. Database searching included all *H. sapiens* entries from UniProt. The database was concatenated with one composed of all protein sequences in that database in reversed order. Searches were performed using a 50-ppm precursor ion tolerance for total protein level profiling. The product ion tolerance was set to 0.9 Da. These wide mass tolerance windows were selected to maximize sensitivity in conjunction with SEQUEST^75^ (v2.3.0.420) searches and linear discriminant analysis^76,77^. TMT labels on lysine residues and peptide N termini (+304.207 Da) and carbamidomethylation of cysteine residues (+57.021 Da) were set as static modifications, and oxidation of methionine residues (+15.995 Da) was set as a variable modification. Peptide-spectrum matches (PSMs) were adjusted to a 2% false discovery rate (FDR)^78,79^. PSM filtering was performed using a linear discriminant analysis, also as described previously^77^, and then assembled further to a final protein-level FDR of 2%^79^.

#### Proteomics data analysis

PSMs were filtered for summed SNR (SNR > 200) across the TMT plex and for precursor signals that contained an isolation purity of >0.5 of the MS1 isolation window. To normalize protein input across TMT channels, all PSM intensities were summed, and the total intensity per channel was sum normalized to the median summed intensity across the TMTpro plex. Protein intensities were generated by summing input-normalized TMT intensities for the constituent peptide PSMs^80^, serving as a weighted average quantification. Comparison among experimental conditions (*n* = 3–4 biological replicates) were conducted by performing a Student’s two-sided *t*-test of normalized log_2_-transformed protein TMT intensities. The resulting *P* values were adjusted for multiple hypothesis correction using the Benjamini–Hochberg approach^81^. For heatmap generation or linear model analysis, replicate protein report ion intensities were normalized to the mean of the biological replicates of either day 0 for the differentiation experiment or to WT control day-12 iNeuron replicates within a given TMTpro plex.

To conduct the linear regression analysis using a single model for the additive combinatorial ER receptor knockout TMT data, we incorporated indicators/dummy variables that can take on one of two possible numerical values (1: contains addition of an ER receptor knockout(s); 0: does not). All replicates were normalized to the mean of the WT control, which was centred at 0, essentially removing the intercept estimation (*ꞵ*_0_) from the model. This was because the TMT protein reporter intensities are not indicative of absolute abundance, and we are interested in understanding the fold change contribution from the addition of each ER receptor knockout. The following indicators/dummy variables and model are presented in Supplementary Note 1. In R using the *lm* function, the beta (*ꞵ*) coefficients and *P* values were extracted from the model, and the *ꞵ* coefficients and Benajmini–Hochberg^81^-adjusted *P* values were leveraged for downstream analysis and figure generation. One can interpret the *ꞵ*_TKO⟶QKO_ for instance as the average FC from the triple knockout to the quadruple knockout, due to the addition of TEX264 KO on the FAM134A^−/−^/B^−/−^/C^−/−^ knockout cells.

Classifications of proteins to various organellar locations or functional groups were performed using manually curated databases from UniProt and are listed in the relevant Supplementary tables. Subcellular annotations were derived from ref. ^26^ with additional cytosol protein location designations from UniProt. ER high sheet and high curvature annotations were extracted from ref. ^2^.

### Statistics and reproducibility

Proteomics data analysis was performed using R Project for Statistical Computing (Version 4.2.2, RRID:SCR_001905, https://www.r-project.org/) within the Rstudio IDE (2022.12.0 Build 353, Posit, RRID:SCR_000432 https://posit.co/). Data visualizations in the form of heatmaps, volcano plots, violin plots, protein abundance profiles and subcellular localization plots were generated using the following R packages: tidyverse (Version 2.0.0, RRID:SCR_019186 https://CRAN.R-project.org/package=tidyverse), dplyr (Version 1.0.10, RRID:SCR_016708 https://cran.r-project.org/web/packages/dplyr/index.html), cowplot (Version 1.1.1, RRID:SCR_018081, https://cran.r-project.org/web/packages/cowplot/index.html), pheatmap (Version 1.0.12, RRID:SCR_016418, https://www.rdocumentation.org/packages/pheatmap/versions/0.2/topics/pheatmap), stringr (Version 1.5.0, RRID:SCR_022813, https://stringr.tidyverse.org/), RColorBrewer (Version 1.1-3, RRID:SCR_016697) https://cran.r-project.org/web/packages/RColorBrewer/index.html), ggrepel (Version 0.9.2, RRID:SCR_017393, https://cran.r-project.org/package=ggrepel), ggplot2 (Version 3.4.1, RRID:SCR_014601, https://cran.r-project.org/web/packages/ggplot2/index.html), purr (Version 1.0.1, https://cran.r-project.org/package=purrr) and tibble (Version, 3.1.8, https://cran.r-project.org/package=tibble). For imaging statistics, GraphPad Prism (Version 9.5.0, RRID:SCR_002798, http://www.graphpad.com/) was used. Mean (for the number of ER structures per nuclei), mean (for the area of axonal ER structures), percent intact nuclei and percent TUNEL-negative nuclei values from each replicate differentiation experiment (*n* = 4 in each experiment) were compared between each knockout and WT using a Mann–Whitney test. For flow cytometry quantification, GraphPad Prism (9.5.0) was used. Each condition had three biological replicates. Brown–Forsythe and Welch one-way ANOVA and Dunnett’s T3 multiple comparisons test (assuming a Gaussian distribution) were used to compare each condition. For imaging and flow cytometry analysis, *P < 0.05; **P < 0.01 and ***P < 0.001. For proteomics datasets, the alpha used for FDR cutoffs was adjusted *P* < 0.05 to consider significance. To compare log_2_FCs for each organelle proteome to a random distribution, a randomized protein selection was generated (100 iterations) keeping the same number of proteins as the perspective organelle. The log_2_FC distribution of this random protein set was compared to the organelle log_2_FC distribution using a Kolmogorov–Smirnov test (two-sided). For analysis of violins, when calculating the degree of change of each ER compartment’s *ꞵ* values from no change (zero), a Wilcoxon one-sided test was used with Bonferroni *P*-value correction applied due to the multiple comparisons. For other analyses of violins comparing the log_2_FCs between two genotypes for each ER compartment, the comparison was made using paired Wilcoxon two-sided tests. All data figures were generated in Adobe Illustrator (Version CS515.0.0, RRID:SCR_010279, http://www.adobe.com/products/illustrator.html) using R (4.1.3), Rstudio IDE (2021.09.3 Build 396, Posit), Fiji Image J (V.2.0.0) and GraphPad Prism (9.5.0).

Unless stated otherwise, all quantitative experiments were performed in triplicate and averages with s.e.m. or s.d., as indicated in the legends. For proteomics experiments, we chose *n* = 2, 3 or 4 biological replicates given the limitation of the available TMT channels and as extensive work in the field has shown that this approach provides the necessary statistical significance. The number of replicates for all TMT experiments is shown in the schematic in the relevant figure. For flow-cytometry experiments, we analysed >10,000 cells with triplicate experiments, which showed consistent results throughout the replicates. Confocal microscopy experiments were performed in triplicate or quadruplicate with *n* biological replicated differentiations unless otherwise noted. The number of replicates for immunoblotting experiments is provided in the figure legends, which were performed in triplicate unless otherwise noted. The number of data points in each plot represents the number of replicates used.

### Reporting summary

Further information on research design is available in the Nature Portfolio Reporting Summary linked to this Article.

## Online content

Any methods, additional references, Nature Portfolio reporting summaries, source data, extended data, supplementary information, acknowledgements, peer review information; details of author contributions and competing interests; and statements of data and code availability are available at 10.1038/s41556-024-01356-4.

## Supplementary information

Supplementary Information Supplemental note with formulae.

Reporting Summary

Supplementary Video 1 Image sequence played at 2 frames per second. Circles indicate co-trafficking of TEX264-GFP/mCh-LC3B positive puncta. Scale bar, 10 μm.

Supplementary Video 2 This example movie includes the cropped region represented in Fig. 4d. Image sequence played at 2 frames per second. Circles indicate co-trafficking FAM134C-GFP/mCh-LC3B positive puncta. Scale bar, 10 μm.

Supplementary Video 3 This example movie is the region represented in Fig. 4e where a TEX264-GFP/mCh-LC3B positive puncta leaves the dilated axonal region. Image sequence played at 2 frames per second. Scale bar, 5 μm.

Supplementary Video 4 See text for details.

Supplementary Tables **Supplementary Table 1**. ER protein designation table. The ER proteome (359 proteins) is organized into functional modules and protein attributes (involved in ER membrane curvature, ER-associated, ER-membrane, ER-lumen or ER-phagy receptor). For proteins with transmembrane segments, the number of segments is indicated after the protein name (_1, _2, and so on) based on data in UniProt. Relevant to assigning ER designations in all other Supplementary Data tables. In .xlsx format.
**Supplementary Table 2**. Total proteome analysis for hESC conversion to iNeurons, WT day 0 to day 12. Relevant to Fig. 1a,b and Extended Data Fig. 1b. In .xlsx format. Details of the experiment underlying these data are provided in a tab in the table. *P* values corresponding to individual protein log_2_FCs were calculated with a Student’s *t*-test (two-sided) and adjusted for multiple hypothesis correction using the Benjamini–Hochberg approach.
**Supplementary Table 3**. Total proteome analysis for WT, ATG12^−/−^, DKO, TKO, QKO, PKO day12 iNeurons. ATG12^−/−^ versus WT relevant to Fig. 1c–e and Extended Data Fig. 1a. ATG12^−/−^, DKO, TKO, QKO, PKO versus WT relevant to Figs. 7b–g, 8a–c,e,f; Extended Data Figs. 5b–d, 7, 8a–c, 9a,b,d, 10a,b,d. In .xlsx format. Details of the experiment underlying the data are provided in a tab in the table. *P* values corresponding to individual protein log_2_FCs were calculated with a Student’s *t*-test (two-sided) and adjusted for multiple hypothesis correction using the Benjamini–Hochberg approach.
**Supplementary Table 4**. Total proteome analysis for WT, ATG12^−/−^, PKO day 0 ESC and day12 iNeurons. Relevant to Extended Data Fig. 2a. In .xlsx format. Details of the experiment underlying the data are provided in a tab in the table. *P* values corresponding to individual protein log_2_FCs were calculated from a Student’s *t*-test (two-sided) and adjusted for multiple hypothesis correction using the Benjamini–Hochberg approach.
**Supplementary Table 5**. Total proteome analysis for WT, ATG12^−/−^, CCPG1^−/−^, TEX264^−/−^, FAM134A^−/−^, FAM134B^−/−^ and FAM134C^−/−^ day-12 iNeurons. Relevant to Fig. 7a and Extended Data Figs. 5a, 8c, 9a, 10b. In .xlsx format. Details of the experiment underlying the data are provided in a tab in the table. *P* values corresponding to individual protein log_2_FCs were calculated from a Student’s *t*-test (two-sided) and adjusted for multiple hypothesis correction using the Benjamini–Hochberg approach.
**Supplementary Table 6**. Total proteome analysis for WT, ATG12^−/−^, PKO^cloneA2^ and PKO^cloneE4^ day-20 iNeurons. Relevant to Extended Data Fig. 6a–c. In .xlsx format. Details of the experiment underlying the data are provided in a tab in the table. *P* values corresponding to individual protein log_2_FCs were calculated from a Student’s *t*-test (two-sided) and adjusted for multiple hypothesis correction using the Benjamini–Hochberg approach.
**Supplementary Table 7**. Total proteome analysis for WT, ATG12^−/−^ and PKO day-12 iNeurons with and without Torin1 inhibitor. Relevant to Extended Data Fig. 6d. In .xlsx format. Details of the experiment underlying the data are provided in a tab in the table. *P* values corresponding to individual protein log_2_FCs were calculated from a Student’s *t*-test (two-sided) and adjusted for multiple hypothesis correction using the Benjamini–Hochberg approach.
**Supplementary Table 8**. Total proteome analysis for WT, ATG12^−/−^ and CALCOCO1^−/−^ day-0 and day-12 iNeurons. Relevant to Extended Data Fig. 6e–i. In .xlsx format. Details of the experiment underlying the data are provided in a tab in the table. *P* values corresponding to individual protein log_2_FCs were calculated from a Student’s *t*-test (two-sided) and adjusted for multiple hypothesis correction using the Benjamini–Hochberg approach.
**Supplementary Table 9**. Total proteome analysis for WT, ATG12^−/−^, FAM134C^−/−^, FAM134CA^−/−^(DKO) and FAM134CB^−/−^ day-12 iNeurons. Relevant to Extended Data Fig. 8d. In .xlsx format. Details of the experiment underlying the data are provided in a tab in the table. *P* values corresponding to individual protein log_2_FCs were calculated from a Student’s *t*-test (two-sided) and adjusted for multiple hypothesis correction using the Benjamini–Hochberg approach.
**Supplementary Table 10**. Total proteome analysis for WT, WT+FAM134C-GFP, FAM134CA^−/−^(DKO) and FAM134CA^−/−^(DKO)+FAM134C-GFP day-12 iNeurons. Relevant to Extended Data Fig. 9g–i. In .xlsx format. Details of the experiment underlying the data are provided in a tab in the table. *P* values corresponding to individual protein log_2_FCs were calculated from a Student’s *t*-test (two-sided) and adjusted for multiple hypothesis correction using the Benjamini–Hochberg approach.
**Supplementary Table 11**. Total proteome analysis for WT, ATG12^−/−^ and TEX264^−/−^+CCPG1^−/−^ day-12 iNeurons. Relevant to Extended Data Fig. 10c. In .xlsx format. Details of the experiment underlying the data are provided in a tab in the table. *P* values corresponding to individual protein log_2_FCs were calculated from a Student’s *t*-test (two-sided) and adjusted for multiple hypothesis correction using the Benjamini–Hochberg approach.

## Source data

Source Data Extended Fig. 1 Unprocessed western blots.

Source Data Extended Fig. 2 Unprocessed western blots.

Source Data Extended Fig. 4 Unprocessed western blots and flow cytometry gating strategy.

Source Data Extended Fig. 6 Unprocessed western blots.

Source Data Extended Fig. 8 Unprocessed western blots.

Source Data Extended Fig. 9 Unprocessed western blots.

Source Data Fig. 7 Proteomics barplot ratio check and normalization strategy.

Source Data Statistical Extended Data Fig. 1 Statistical source data for Extended Fig. 1.

Source Data Statistical Extended Data Fig. 2 Statistical source data for Extended Fig. 2.

Source Data Statistical Extended Data Fig. 4 Statistical source data for Extended Fig. 4.

Source Data Statistical Extended Data Fig. 9 Statistical source data for Extended Fig. 9.

Source Data Statistical Fig. 2 Statistical source data for Fig. 2.

Source Data Statistical Fig. 3 Statistical source data for Fig. 3.

Source Data Statistical Fig. 4 Statistical source data for Fig. 4.

Source Data Statistical Fig. 6 Statistical source data for Fig. 6.

Source Data Statistical Fig. 8 Statistical source data for Fig. 8.

## Data Availability

We used canonical protein entries from the human reference proteome database in our study (UniProt Swiss-Prot – 2019-01; https://ftp.uniprot.org/pub/databases/uniprot/previous_major_releases/release-2019_01/). The mass spectrometry proteomics data have been deposited to the ProteomeXchange Consortium via the PRIDEpartner repository^82^ with the dataset identifiers PXD041069 (Supplementary Data Tables 2, 3, 5, 7 and 11) and PXD046646 (Supplementary Tables 4, 6, 8, 9 and 10). Representative tomograms are available in the Electron Microscopy Data Bank under the following accession codes: EMD-19346 (Fig. 5c–e) and EMD-19194 (Fig. 5f–h). Previously published proteomics data^25^ that were re-analysed in Fig. 1 are available under accession code MSV000087961. Data supporting the findings of this study are available from the corresponding author on request. Source data are provided with this paper. Additional source data for this Article can also be found at 10.5281/zenodo.10606989.

## Code availability

Code for proteomics data analysis and relevant figure generation is available at 10.5281/zenodo.10606794 and on Github at https://github.com/harperlaboratory/iNeuron_ERphagy.git.
