# Author Correction: Combinatorial selective ER-phagy remodels the ER during neurogenesis

**DOI:** 10.1038/s41556-025-01670-5

**Published:** 2025-04-15

**Authors:** Melissa J. Hoyer, Cristina Capitanio, Ian R. Smith, Julia C. Paoli, Anna Bieber, Yizhi Jiang, Joao A. Paulo, Miguel A. Gonzalez-Lozano, Wolfgang Baumeister, Florian Wilfling, Brenda A. Schulman, J. Wade Harper

**Affiliations:** 1 Department of Cell Biology, Harvard Medical School, Boston, MA USA; 2 Aligning Science Across Parkinson’s (ASAP) Collaborative Research Network, Chevy Chase, MD USA; 3 Department of Molecular Machines and Signaling, Max Planck Institute of Biochemistry, Martinsried, Germany; 4 Department of Molecular Structural Biology, Max Planck Institute of Biochemistry, Martinsried, Germany; 5 Mechanisms of Cellular Quality Control, Max Planck Institute of Biophysics, Frankfurt am Main, Germany; 6 Present Address: Velia Therapeutics, San Diego, CA USA

**Keywords:** Endoplasmic reticulum, Macroautophagy

Correction to: *Nature Cell Biology* 10.1038/s41556-024-01356-4, published online 1 March 2024.

In the version of the article initially published, in the “Gene editing” section of the Methods, the FAM134A sgRNA target sequence was listed as “TAATACGACTCACTATAG” but should have been “TGCATCACAAACACGACAAGAGG” and the FAM134C sgRNA target sequence was listed as “AACTTGAGCTGTCAGACCAACA” but should have been “CTCTCATTGTCTAATGCGTC”. These errors have now been corrected in the HTML and PDF versions of the article.

