# Supplementary material for: Combinatorial selective ER-phagy remodels the ER during neurogenesis
Source: Nat Cell Biol. 2024 Mar 1;26(3):378–92. doi: 10.1038/s41556-024-01356-4 (PMC10940164; doi:10.1038/s41556-024-01356-4)
Supplement: Supplementary file 1 — Supplemental note with formulae. [file 41556_2024_1356_MOESM1_ESM.pdf]

nature cell biology

Article https://doi.org/10.1038/s41556-024-01356-4

Combinatorial selective ER-phagy remodels the ER during neurogenesis

In the format provided by the authors and unedited

Supplemental note 1. Equations used for the linear model.

Indicators/dummy variables:

$$x_{i1} = \begin{cases} 1 & \text{if } i\text{th sample contains FAM134A}^{-/-}/\text{C}^{-/-} \\ 0 & \text{if } i\text{th sample does not contain FAM134A}^{-/-}/\text{C}^{-/-} \end{cases}$$

$$x_{i2} = \begin{cases} 1 & \text{if } i\text{th sample contains FAM134A}^{-/-}/\text{B}^{-/-}\ /\text{C}^{-/-} \\ 0 & \text{if } i\text{th sample does not contain FAM134A}^{-/-}/\text{B}^{-/-}\ /\text{C}^{-/-} \end{cases}$$

$$x_{i3} = \begin{cases} 1 & \text{if } i\text{th sample contains FAM134A}^{-/-}/\text{B}^{-/-}\ /\text{C}^{-/-}\ /\text{TEX264}^{-/-} \\ 0 & \text{if } i\text{th sample does not contain FAM134A}^{-/-}/\text{B}^{-/-}\ /\text{C}^{-/-}/\text{TEX264}^{-/-} \end{cases}$$

$$x_{i4} = \begin{cases} 1 & \text{if } i\text{th sample contains FAM134A}^{-/-}/\text{B}^{-/-}\ /\text{C}^{-/-}\ /\text{TEX264}^{-/-}/\text{CCPG1}^{-/-} \\ 0 & \text{if } i\text{th sample does not contain FAM134A}^{-/-}/\text{B}^{-/-}\ /\text{C}^{-/-}/\text{TEX264}^{-/-}/\text{CCPG1}^{-/-} \end{cases}$$

The model below was then used to estimate beta coefficients (β) for each step-wise addition of ER receptor knockout(s) with inherent technical noise from the MS acquisition and reporter quantification.

$$Y = \beta_0 + \beta_{WT\rightarrow DKO}x_{i1} + \beta_{DKO\rightarrow TKO}x_{i2} + \beta_{TKO\rightarrow QKO}x_{i3} + \beta_{QKO\rightarrow PKO}x_{i4} + \epsilon_i$$

Thus:

$$\beta_0 + \epsilon_i \quad \longrightarrow \text{if } i\text{th sample is WT}$$

$$\beta_0 + \beta_{WT\rightarrow DKO}x_{i1} + \epsilon_i \quad \longrightarrow \text{if } i\text{th sample is a FAM134A}^{-/-}/\text{C}^{-/-}\ \text{KO}$$

$$\beta_0 + \beta_{WT\rightarrow DKO}x_{i1} + \beta_{DKO\rightarrow TKO}x_{i2} + \epsilon_i \quad \longrightarrow \text{if } i\text{th sample is a FAM134A}^{-/-}/\text{B}^{-/-}\ /\text{C}^{-/-}\ \text{KO}$$

$$\beta_0 + \beta_{WT\rightarrow DKO}x_{i1} + \beta_{DKO\rightarrow TKO}x_{i2} + \beta_{TKO\rightarrow QKO}x_{i3} + \epsilon_i$$
$$\longrightarrow \text{if } i\text{th sample is a FAM134A}^{-/-}/\text{B}^{-/-}\ /\text{C}^{-/-}/\text{TEX264}^{-/-}\ \text{KO}$$

$$\beta_0 + \beta_{WT\rightarrow DKO}x_{i1} + \beta_{DKO\rightarrow TKO}x_{i2} + \beta_{TKO\rightarrow QKO}x_{i3} + \beta_{QKO\rightarrow PKO}x_{i4} + \epsilon_i$$

$\longrightarrow$ if ith sample is a FAM134A$^{-/-}$/B$^{-/-}$ /C$^{-/-}$/TEX264$^{-/-}$/CCPG1$^{-/-}$ KO
