# Supplementary material for: Combinatorial selective ER-phagy remodels the ER during neurogenesis
Source: Nat Cell Biol. 2024 Mar 1;26(3):378–92. doi: 10.1038/s41556-024-01356-4 (PMC10940164; doi:10.1038/s41556-024-01356-4)
Supplement: Supplementary file 2 — Reporting Summary [file 41556_2024_1356_MOESM2_ESM.pdf]

nature research

Corresponding author(s): J. Wade Harper

Last updated by author(s): 3/31/2023

Reporting Summary

Nature Research wishes to improve the reproducibility of the work that we publish. This form provides structure for consistency and transparency in reporting. For further information on Nature Research policies, see Authors & Referees and the Editorial Policy Checklist.

Statistics

For all statistical analyses, confirm that the following items are present in the figure legend, table legend, main text, or Methods section.

n/a	Confirmed	
☐	☒	The exact sample size (*n*) for each experimental group/condition, given as a discrete number and unit of measurement
☐	☒	A statement on whether measurements were taken from distinct samples or whether the same sample was measured repeatedly
☐	☒	The statistical test(s) used AND whether they are one- or two-sided<br>*Only common tests should be described solely by name; describe more complex techniques in the Methods section.*
☒	☐	A description of all covariates tested
☐	☒	A description of any assumptions or corrections, such as tests of normality and adjustment for multiple comparisons
☐	☒	A full description of the statistical parameters including central tendency (e.g. means) or other basic estimates (e.g. regression coefficient) AND variation (e.g. standard deviation) or associated estimates of uncertainty (e.g. confidence intervals)
☐	☒	For null hypothesis testing, the test statistic (e.g. *F*, *t*, *r*) with confidence intervals, effect sizes, degrees of freedom and *P* value noted<br>*Give P values as exact values whenever suitable.*
☒	☐	For Bayesian analysis, information on the choice of priors and Markov chain Monte Carlo settings
☒	☐	For hierarchical and complex designs, identification of the appropriate level for tests and full reporting of outcomes
☒	☐	Estimates of effect sizes (e.g. Cohen's *d*, Pearson's *r*), indicating how they were calculated

Our web collection on statistics for biologists contains articles on many of the points above.

Software and code

Policy information about availability of computer code

Data collection	Orbitrap Eclipse Tribrid Mass Spectrometer (Cat#FSN04-10000) with FAIMS Pro Interface (#FMS02-10001) - Thermo Fisher Scientific<br>Orbitrap Fusion Lumos Tribrid MS (Cat#IQLAAEGAAPFADBMBHQ) with or without FAIMS Pro Interface (#FMS02-10001) - Thermo Fisher Scientific<br>BioRad ChemiDoc Imaging System<br>Yokogawa CSU-X1 spinning disk confocal on a Nikon Ti-E inverted microscope equipped with Nikon Plan Apo 40x/1.30 N.A or 100x/1.40 N.A objective, 445nm (75mW), 488nm (100mW), 561nm (100mW) & 642nm (100mW) laser lines controlled by AOT, Nikon Perfect Focus System, Hamamatsu ORCA-Fusion BT sCMOS (6.45 µm2 photodiode)<br>Attune NxT Flow Cytometer (Cat#A28993)- Thermo Fisher Scientific<br>Illumina MiSeq<br>Sony Biotechnology SH800S Cell Sorter<br>Vitrobot Mark IV – Thermo Fisher Scientific<br>SP8 cryo-confocal laser scanning microscope with cryo stage -Leica<br>Krios G4 with Selectris X energy filter and Falcon 4i camera -Thermo Fisher Scientific
Data analysis	1. Prism; GraphPad, v9.5.0 https://www.graphpad.com/scientific-software/prism/ (RRID:SCR_002798)<br>2. SEQUEST-HT ; Eng et al., (1994) J Am Soc Mass Spectrom. 5 (11): 976-989. Implementation in Proteome Discoverer (v2.3.0.420 – Thermo Fisher<br>3. Comet (v2018.01 rev. 2); Eng, J.K. et al. (2013), Proteomics 13, 22-24.<br>4. FlowJoTM; V10.5.2 https://www.flowjo.com (RRID:SCR_008520)<br>5. BioRad Image Lab Software v5.2.5<br>6. FiJi ImageJ V.2.0.0 https://imagej.net/Fiji (RRID:SCR_002285)<br>6. Rstudio (2022.12.0 Build 353) https://posit.co/ (RRID:SCR_000432) + R(4.2.2) https://www.r-project.org/ (RRID:SCR_001905)

7. R package: tidyverse (2.0.0) https://CRAN.R-project.org/package=tidyverse (RRID:SCR_019186)
8. R package: dplyr (1.0.10) https://cran.r-project.org/web/packages/dplyr/index.html (RRID:SCR_016708)
9. R package: cowplot (1.1.1) https://cran.r-project.org/web/packages/cowplot/index.html (RRID:SCR_018081)
10. R package: pheatmap (1.0.12) https://www.rdocumentation.org/packages/pheatmap/versions/0.2/topics/pheatmap (RRID:SCR_016418)
11. R package: stringr (1.5.0) https://stringr.tidyverse.org/ (RRID:SCR_022813)
12. R package: RColorBrewer (1.1-3) https://cran.r-project.org/web/packages/RColorBrewer/index.html (RRID:SCR_016697)
13. R package: ggrepel (0.9.2) https://cran.r-project.org/package=ggrepel (RRID:SCR_017393)
14. R package: ggplot2 (3.4.1) https://cran.r-project.org/web/packages/ggplot2/index.html (RRID:SCR_014601)
15: R package: tibble (3.1.8) https://cran.r-project.org/package=tibble
16. R package: purr (1.0.1) https://cran.r-project.org/package=tibble
17. Adobe Illustrator (CS5(15.0.0)) http://www.adobe.com/products/illustrator.html (RRID:SCR_010279)
18. CellProfiler (4.2.5) http://cellprofiler.org (RRID:SCR_007358)
19. https://github.com/harperlaboratory/iNeuron_ERphagy.git (code for analysis of data and figure generation, R-scripts)
20. Huygens Deconvolution software (version 21.10.0p0, SVI, The Netherlands) https://svi.nl/HuygensSoftware (RRID:SCR_014237)
21. Tomo5 (Version 5.12.0, Thermo Fisher Scientific). https://tomopy.readthedocs.io/ (RRID:SCR_021359)
22. IMOD (v.4.10.49, RRID:SCR_003297, https://bio3d.colorado.edu/imod/ (RRID:SCR_003297)
23. TomoMAN wrapper scripts Version 08042020 (https://doi.org/10.5281/ZENODO.4110737)
24. MATLAB (Version R2019b) (RRID:SCR_001622)
25. Relion Motioncorr2 (version 4.0) https://emcore.ucsf.edu/cryoem-software (RRID:SCR_016499)
26. cryo-CARE v0.0.1 (https://github.com/juglab/cryoCARE_T2T)
27. ChimeraX (version 1.6.1, https://www.cgl.ucsf.edu/chimera/ (RRID:SCR_015872)
28. Dragonfly (version 2022.2, Comet Technologies Canada Inc., Montreal, Canada.) https://www.theobjects.com/dragonfly/index.html
29. TomosegmemTV version 04-23 (https://sites.google.com/site/3demimageprocessing/tomosegmemtv)
30. Membrain-Seg (https://github.com/teamtomo/membrain-seg), pre-trained model:Version 9
31. Amira Imaging software (Thermo Fisher Scientific), version 2021.2 https://www.thermofisher.com/id/en/home/electron-microscopy/products/software-em-3d-vis/amira-software.html
32. Fiji BigWarp plugin (version 9.0.0)
33. Python version 3.9.7 with pandas version 1.3.0 (https://pandas.pydata.org/, RRID:SCR_018214)
34. Matplotlib (version 3.3.0, https://matplotlib.org/,RRID:SCR_008624)
35. Seaborn (version 0.11.0, https://seaborn.pydata.org/, RRID:SCR_018132)
36. Nikon Elements (version AR) RRID:SCR_014329
37. Microsoft Excel (version 16.81) https://www.microsoft.com/en-gb/ (RRID:SCR_016137)
38. Msconvert (Version 3.0) https://bio.tools/msconvert

For manuscripts utilizing custom algorithms or software that are central to the research but not yet described in published literature, software must be made available to editors/reviewers. We strongly encourage code deposition in a community repository (e.g. GitHub). See the Nature Research guidelines for submitting code & software for further information.

Data

Policy information about availability of data

All manuscripts must include a data availability statement. This statement should provide the following information, where applicable:

- Accession codes, unique identifiers, or web links for publicly available datasets
- A list of figures that have associated raw data
- A description of any restrictions on data availability

Data Availability
We used canonical protein entries from the Human reference proteome database in our study (UniProt Swiss-Prot – 2019-01; https://ftp.uniprot.org/pub/databases/uniprot/previous_major_releases/release-2019_01/). The mass spectrometry proteomics data have been deposited to the ProteomeXchange Consortium via the PRIDEpartner repository82 with the dataset identifiers PXD041069 (Supplementary data table 2, 3, 5, 7, 11) and PXD046646 (Supplemental tables 4, 6, 8, 9, 10). Previously published proteomics data25 that were re-analysed in Figure 1 are available under accession code MSV000087961. Representative tomograms are available in the Electron Microscopy Data Bank under the following accession codes: EMD-19346 (Fig.5c-e), EMD-19194 (Fig. 5f-h). Source data have been provided in Source Data. All other data supporting the findings of this study are available from the corresponding author on reasonable request.

Field-specific reporting

Please select the one below that is the best fit for your research. If you are not sure, read the appropriate sections before making your selection.

☒ Life sciences ☐ Behavioural & social sciences ☐ Ecological, evolutionary & environmental sciences

For a reference copy of the document with all sections, see nature.com/documents/nr-reporting-summary-flat.pdf

Life sciences study design

All studies must disclose on these points even when the disclosure is negative.

Sample size	No sample-size calculation was performed. The sample size for each experiment is included in the respective figure legend. For proteomics experiments, we chose n=2, 3 or 4 biological replicates given the limitation of the available TMT channels and extensive work in the field has shown that this approach provides the necessary statistical significance. The number of replicates for all TMT experiments is shown in the schematic in the relevant figure. For Flow-cytometry experiments, we analyzed >10,000 cells with triplicate experiments which showed consistent results through-out the replicates. Confocal microscopy experiments were done in triplicate or quadruplicate with n=biological

replicated differentiations unless otherwise noted. The number of replicates for immunoblotting experiments is provided in the figure legends and is performed in triplicate unless otherwise noted. The number of data points in each plot represents the number of replicates used. Sample size was determined based on similar studies in this field. e.g. An et al Systematic quantitative analysis of ribosome inventory during nutrient stress. Ordureau A, Kraus F, Zhang J, An H, Park S, Ahfeldt T, Paulo JA, Harper JW. Temporal proteomics during neurogenesis reveals large-scale proteome and organelle remodeling via selective autophagy. Mol Cell. 2021 Dec 16;81(24):5082-5098.e11. doi: 10.1016/j.molcel.2021.10.001.

Data exclusions	No data were excluded from the analyses.
Replication	We confirm that all attempts at replication were successful. The number of biological replicates is provided for each experiment in the figure legend.
Randomization	No randomization was necessary. Mass spectrometry and immunoblot samples were measured sequentially. Images were automatically acquired for the data analysis by high throughput imaging based methods.
Blinding	Blinding was not relevant in this study, because all the data were analyzed using unbiased methods.

Reporting for specific materials, systems and methods

We require information from authors about some types of materials, experimental systems and methods used in many studies. Here, indicate whether each material, system or method listed is relevant to your study. If you are not sure if a list item applies to your research, read the appropriate section before selecting a response.

Materials & experimental systems

n/a	Involved in the study	
☐	☒	Antibodies
☐	☒	Eukaryotic cell lines
☒	☐	Palaeontology
☒	☐	Animals and other organisms
☒	☐	Human research participants
☒	☐	Clinical data

Methods

n/a	Involved in the study	
☒	☐	ChIP-seq
☐	☒	Flow cytometry
☒	☐	MRI-based neuroimaging

Antibodies

Antibodies used

ATG5 Rabbit Monoclonal (D5F5U) Antibody Cell Signaling Technology 12994S, Lot5, WB 1:1000, RRID:AB_2630393;
FAM134B Rabbit Polyclonal Antibody Proteintech 21537-1-AP, lot00100765, WB 1:1000 RRID:AB_2878879;
FAM134C Rabbit Polyclonal Antibody Sigma-Aldrich HPA016492, lotR06641, WB 1:1000, RRID:AB_1853027;
CCPG1 Rabbit Monoclonal (E3C5G) Antibody Cell Signaling Technology 80158, lot1, WB 1:1000, RRID:AB_2935809;
TEX264 Rabbit Polyclonal Antibody Sigma-Aldrich HPA017739, lot000012723, WB 1:1000, RRID:AB_1857910;
REEP1 Rabbit Polyclonal Antibody Sigma-Aldrich HPA058061, lotR81573, WB 1:1000, RRID:AB_2683591;
REEP4 Rabbit Polyclonal Antibody Sigma-Aldrich HPA042683, lotR39936, WB 1:1000, RRID:AB_2571730;
REEP5 Rabbit Polyclonal Antibody Proteintech 14643-1-AP, lot00050540, WB 1:1000, RRID:AB_2178440;
RTN3 Mouse Monoclonal (F-6))Antibody Santa Cruz sc-374599, lot10922, WB 1:1000, RRID:AB_10986405;
CKAP-4/p63 Sheep Polyclonal Antibody RD Biosciences AF7355, lotCGDGG012105B, WB 1:1000, RRID:AB_10972125;
CKAP4 Rabbit Polyclonal Antibody Proteintech 16686-1-AP, lot0052093, WB 1:1000, RRID:AB_2276275;
hFAB™ Rhodamine Anti-Tubulin Antibody BioRad 12004166, lot64512247, WB 1:10,000, RRID:AB_2884950;
HSP90 mouse monoclonal (4F10) Antibody Santa Cruz sc-69703, lotJ2721, WB 1:10,000, RRID:AB_2121191;
GAPDH XP Rabbit Monoclonal (D16H11) Antibody Cell Signaling Technology 5174, lot8, WB 1:1000, RRID:AB_10622025;
CREB-2/ATF-4 Mouse Monoclonal (B-3) Antibody sc-390063, lotJ2021, WB 1:1000, RRID:AB_2810998;
VAPA Rabbit monoclonal (EPR13589(B)) Antibody Abcam ab181067, lotGR164232-2, WB 1:1000, RRID: AB_3073850;
RTN1 (Isoform RTN1-C) Rabbit Polyclonal Antibody Proteintech 15048-1-AP, lot00043268, WB 1:1000, RRID:AB_2185981;
Goat anti-Rabbit IgG HRP conjugate Bio-Rad 1706515, lot64559210, WB 1:3000, RRID:AB_11125142;
Goat anti-Mouse IgG HRP conjugate Bio-Rad 1706516, lot64526160; WB 1:3000, RRID:AB_11125547;
Neurofilament heavy polypeptide mouse monoclonal (NF-01) antibody Abcam ab7795, lotGR3448163-1, IF 1:300, RRID:AB_306084;
MAP2 Guinea Pig Polyclonal Antibody Synaptic systems 188004, lot6-49, IF 1:300, RRID:AB_2138181;
Nogo-A (RTN4) Mouse Monoclonal (C-4) Antibody Santa Cruz sc-271878, lotD2420, IF 1:300, RRID:AB_10709573;
Calnexin Rabbit Polyclonal Antibody Proteintech 10427-2-AP, lot00094417, IF 1:300, RRID:AB_2069033;
Goat anti-mouse Alexa488 Thermo Fisher Scientific A-11001, lot2379467, IF 1:300, RRID:AB_2534069;
Goat anti-chicken Alexa488 Thermo Fisher Scientific A11039, lot218068, IF 1:300, RRID:AB_2534096;
Goat anti-rabbit Alexa568 Thermo Fisher Scientific A-11011, lot2500544, IF 1:300, RRID:AB_143157;
Goat anti-rabbit Alexa647 Thermo Fisher Scientific A27040, lot2659317, IF 1:300, RRID:AB_2536101;
Goat anti-guinea pig Alexa488 Thermo Fisher Scientific A-11073, lot38320A, IF 1:300, RRID:AB_2534117;
Goat anti-guinea pig Alexa647 Thermo Fisher Scientific A-21450, lot2446026, IF 1:300, RRID:AB_141882

Validation

CCPG1, TEX264, FAM134B and FAM134C specificity determined in this study by CRISPR deletion or tagging of endogenous gene.

For all other antibodies, the supplier website provided validation of the antibody for use in human cell lines and articles citing the use of the antibody were also listed.

Eukaryotic cell lines

Policy information about cell lines

Cell line source(s)	HEK293T ATCC CRL-3216 CVCL_0063<br>H9 hESC Wicell WA9 CVCL_9773<br>KOLF2.1 iPS cells (Jackson Labs) CVCL_D1J6
Authentication	ATCC preforms quality testing to ensure authentication of the HEK293T cell line using Short Tandem Repeat Analysis (STR). KOLF2.1 cells were provided by Jackson Labs and are authenticated at Jackson Labs using a Fluidigm assay. H9 ES cells (from WiCell) are authenticated by WiCell using G-band karyotyping and Short Tandem Repeat Analysis (STR). For genetically edited H9 hESCs we confirm via karyotyping and those results are in the extended figure 4e. Successful conversion of stem cells to NGN2 induced neurons was confirm via mass spectrometry analysis of neuronal markers. No additional authentications were preformed.
Mycoplasma contamination	All cell lines were found to be free of mycoplasma using Mycoplasma Plus PCR assay kit (Agilent).
Commonly misidentified lines<br>(See ICLAC register)	none

Flow Cytometry

Plots

Confirm that:

☒ The axis labels state the marker and fluorochrome used (e.g. CD4-FITC).

☒ The axis scales are clearly visible. Include numbers along axes only for bottom left plot of group (a 'group' is an analysis of identical markers).

☒ All plots are contour plots with outliers or pseudocolor plots.

☒ A numerical value for number of cells or percentage (with statistics) is provided.

Methodology

Sample preparation	No tissue processing were used. This study did not involve any human subjects, as stated in the ethical regulations statement at the beginning of the METHODS section as the source of the commercial stem cells have been de-identified.
Instrument	Attune NxT Flow Cytometer- Thermo Fisher Scientific
Software	FlowJoTM; V10.5.2 https://www.flowjo.com
Cell population abundance	10,000 cells were recorded per replicate
Gating strategy	1. live cells were gated by SSC1 hight/FSC1 hight (G1) followed by live cells by SSC1 hight/SSC1-width (G2). 2. Keima signal was measured by 405ex/620(20)em and 561ex/620(20)em and data exported to prism for ratio-metric calculation.

☒ Tick this box to confirm that a figure exemplifying the gating strategy is provided in the Supplementary Information.
