# Supplementary figures and images for: Combinatorial selective ER-phagy remodels the ER during neurogenesis
Source: Nat Cell Biol. 2024 Mar 1;26(3):378–92. doi: 10.1038/s41556-024-01356-4 (PMC10940164; doi:10.1038/s41556-024-01356-4)

Extended  
Figure  
1e

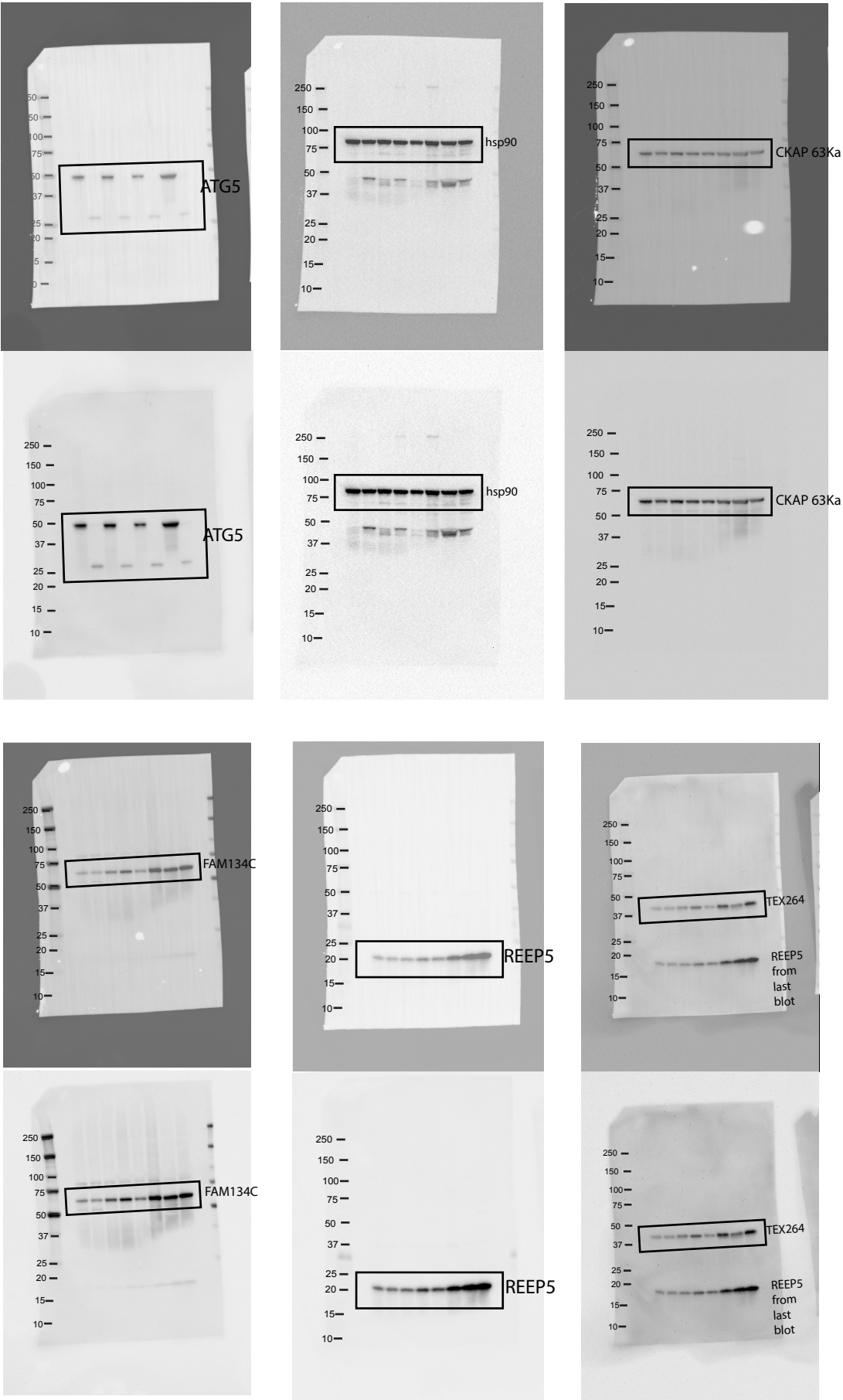

Supplement: Supplementary file 8 — Unprocessed western blots. [file 41556_2024_1356_MOESM8_ESM.pdf]

Extended  
Figure  
6c

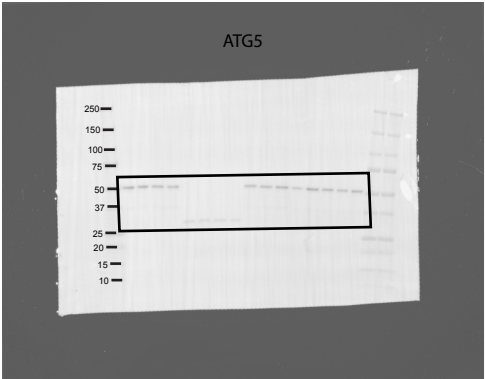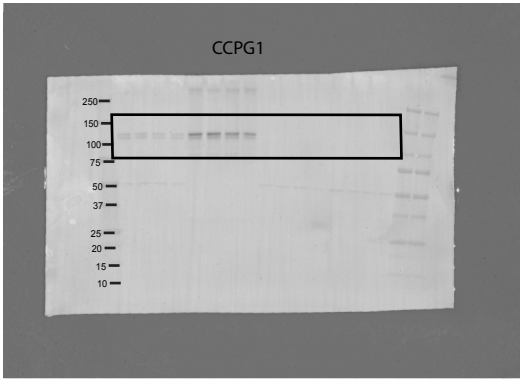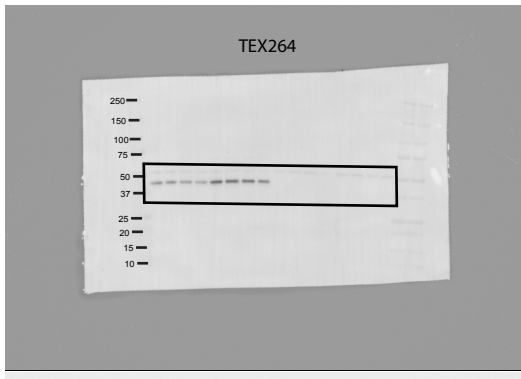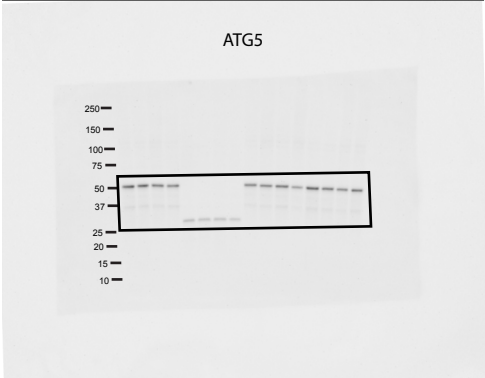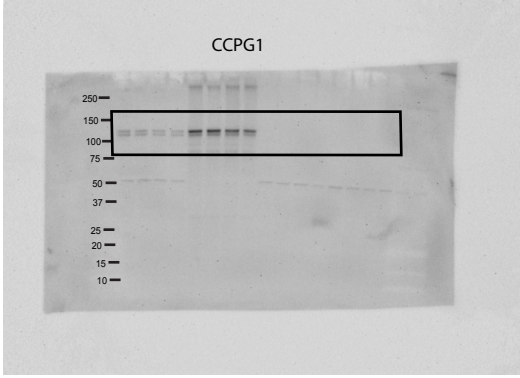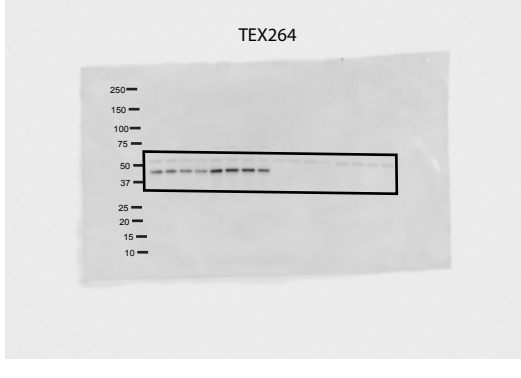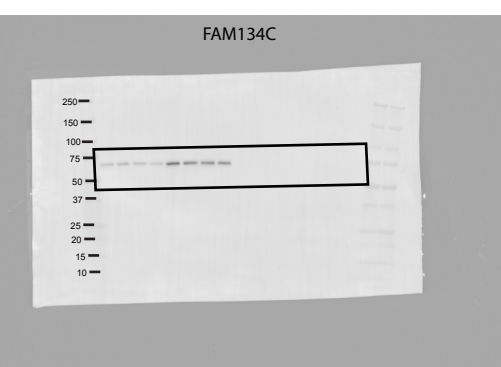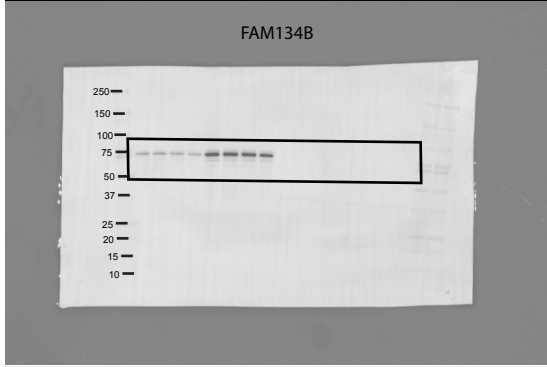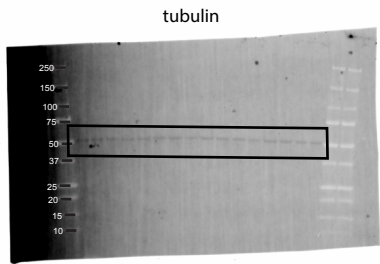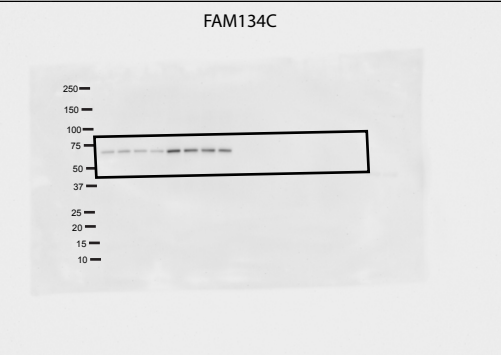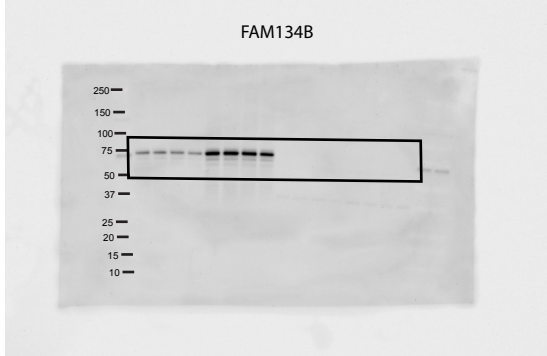

Supplement: Supplementary file 11 — Unprocessed western blots. [file 41556_2024_1356_MOESM11_ESM.pdf]

Extended Figure 9c

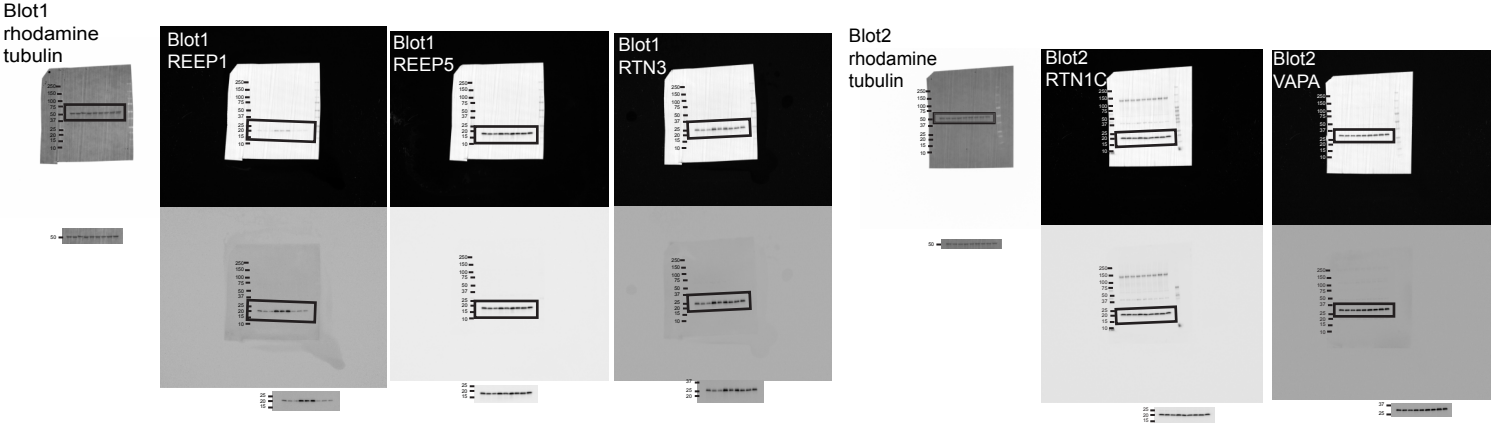

Extended Figure 9f

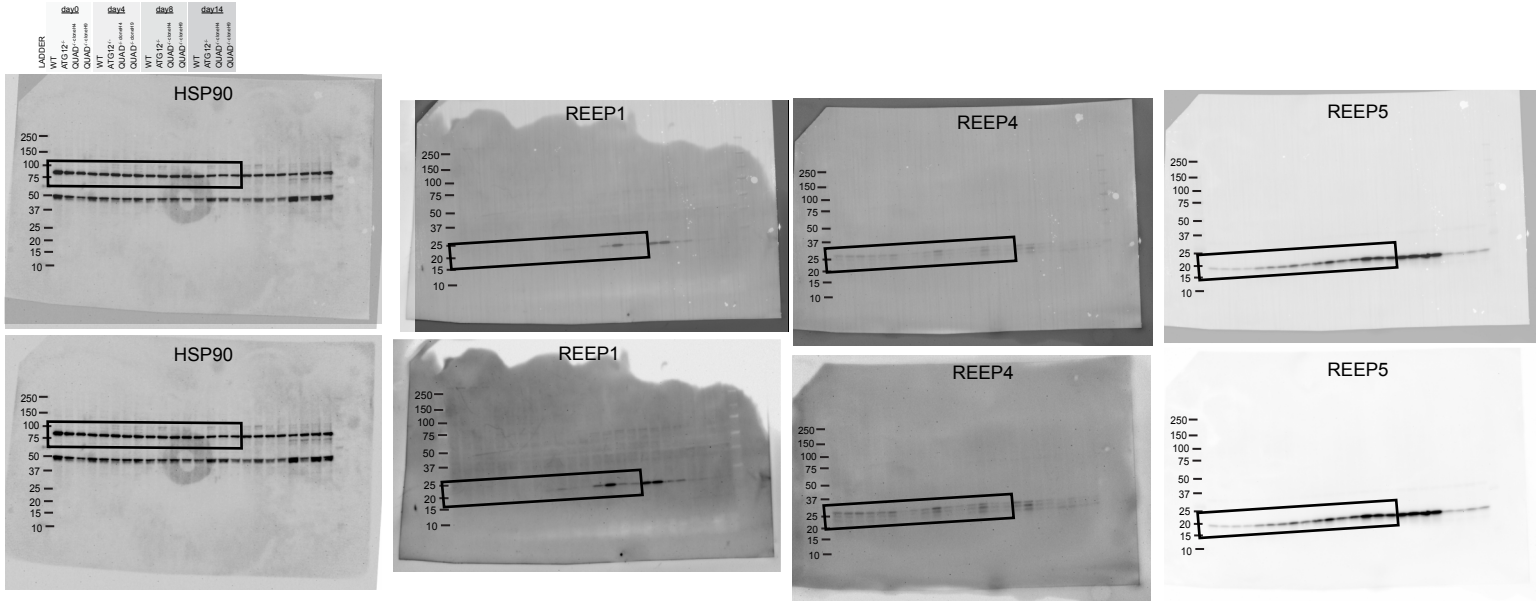

Extended Figure 9g

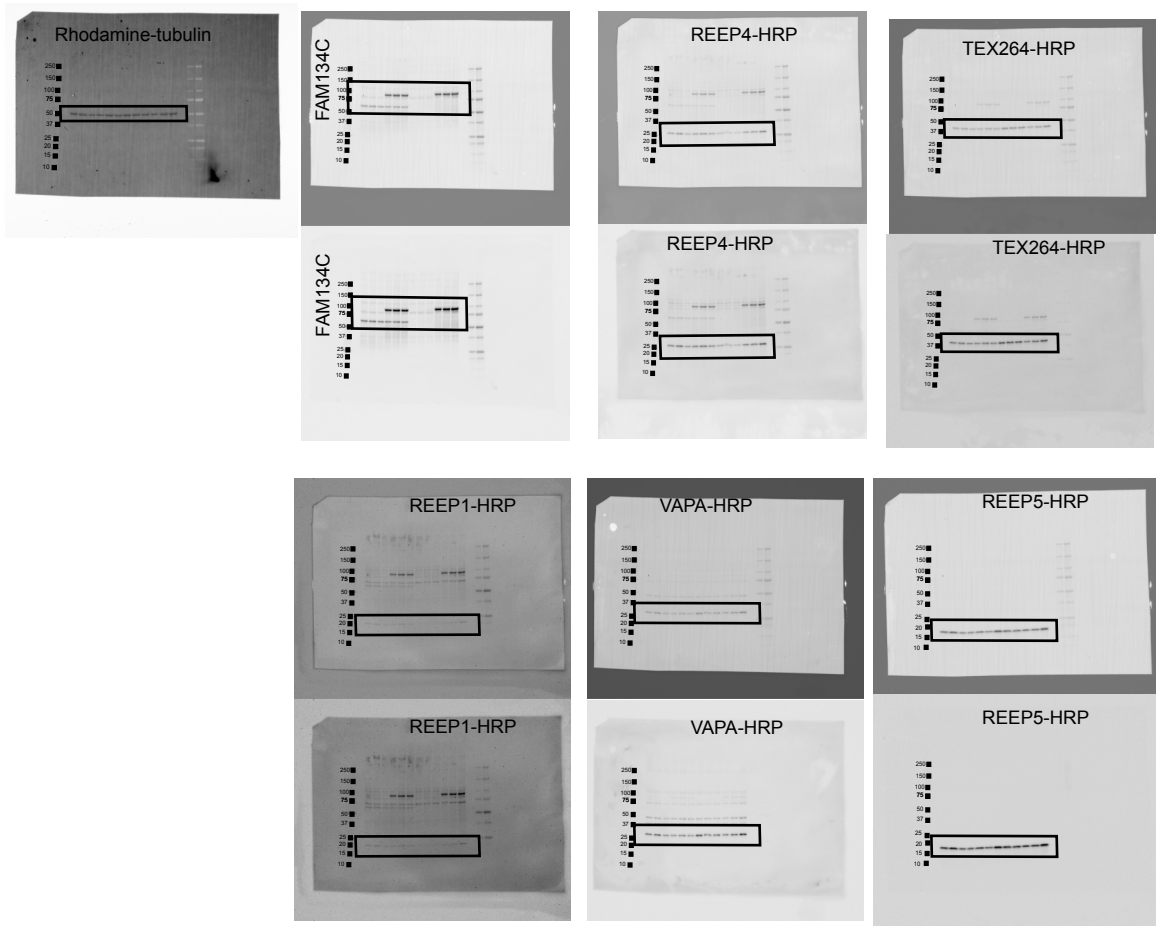

Supplement: Supplementary file 13 — Unprocessed western blots. [file 41556_2024_1356_MOESM13_ESM.pdf]
