# Supplementary material for: Combinatorial selective ER-phagy remodels the ER during neurogenesis
Source: Nat Cell Biol. 2024 Mar 1;26(3):378–92. doi: 10.1038/s41556-024-01356-4 (PMC10940164; doi:10.1038/s41556-024-01356-4)

# Hoyer et al source data
# All uncropped blots and gels
# Extended Figure 2

Extended Figure 2e

ATF4

ATF4

rhodaimine tubuin

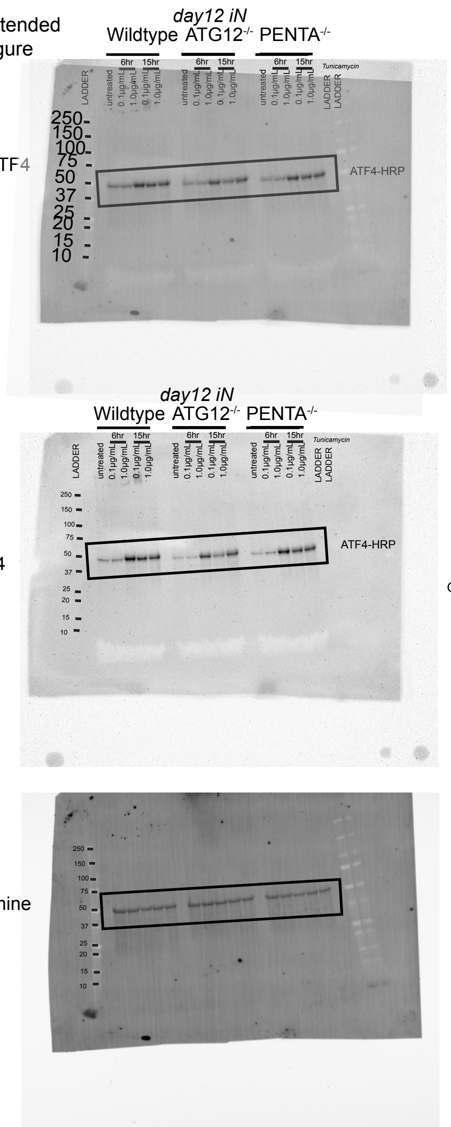


Extended Figure 2f

xbp-1

GAPDH

GAPDH

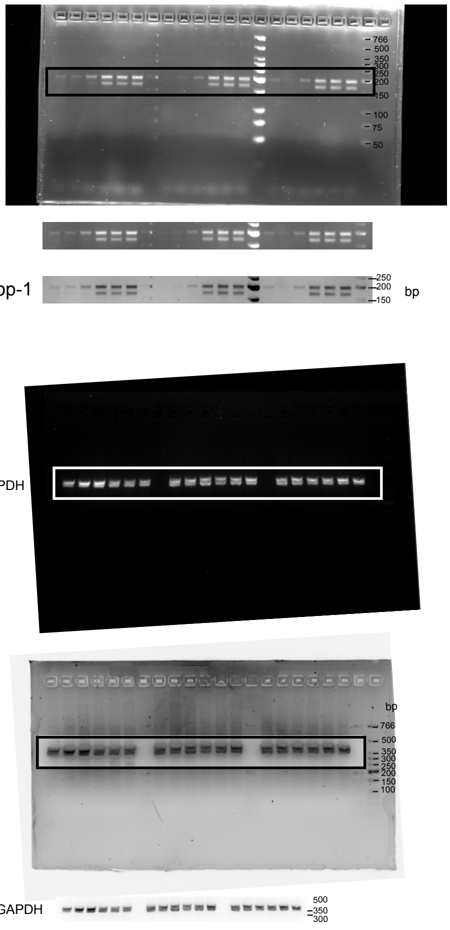


Extended Figure 2h

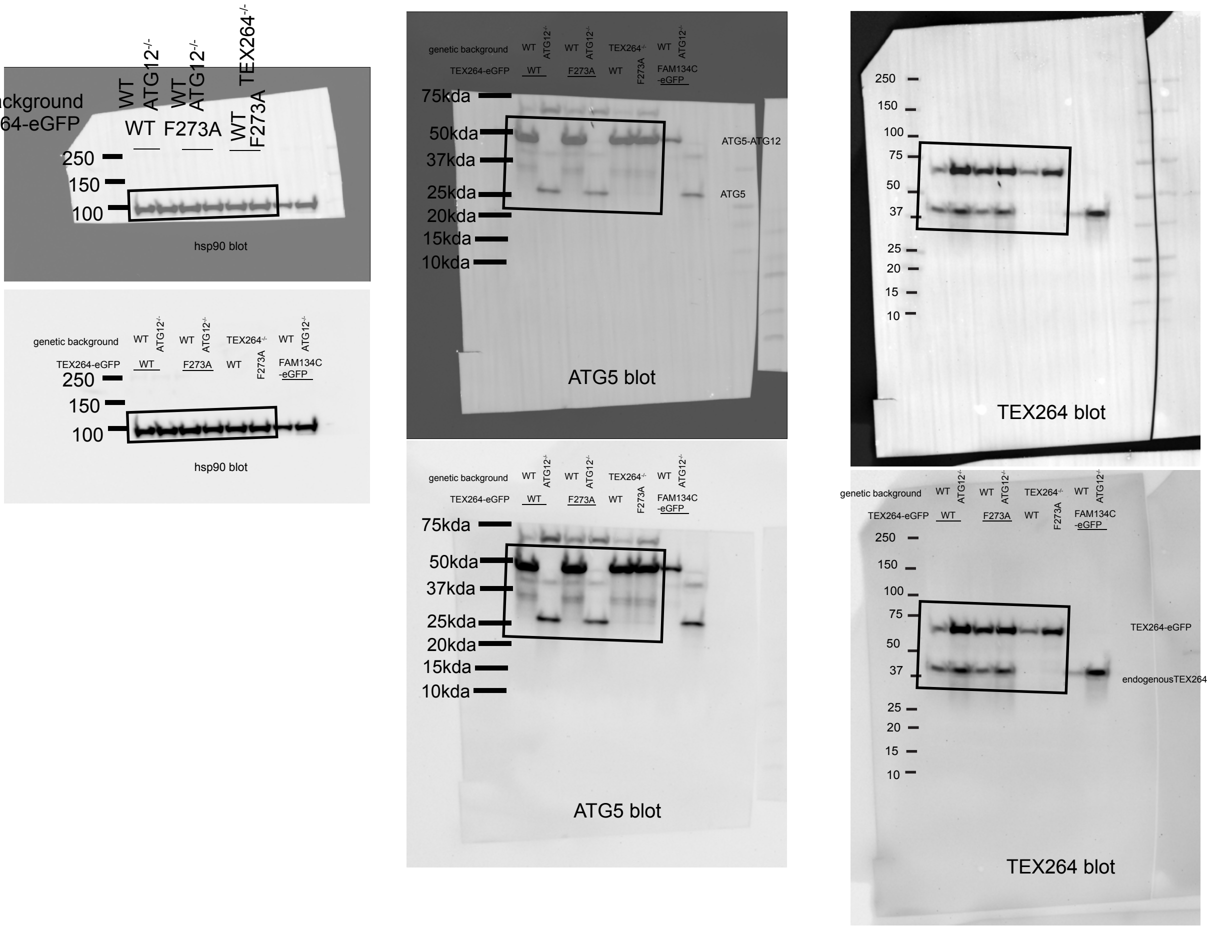

Supplement: Supplementary file 9 — Unprocessed western blots. [file 41556_2024_1356_MOESM9_ESM.pdf]
