# Supplementary material for: Combinatorial selective ER-phagy remodels the ER during neurogenesis
Source: Nat Cell Biol. 2024 Mar 1;26(3):378–92. doi: 10.1038/s41556-024-01356-4 (PMC10940164; doi:10.1038/s41556-024-01356-4)

Hoyer et al source data

Extended Figure 4h
Example flow
cytometry gating

Normal ER-Keima gating example:

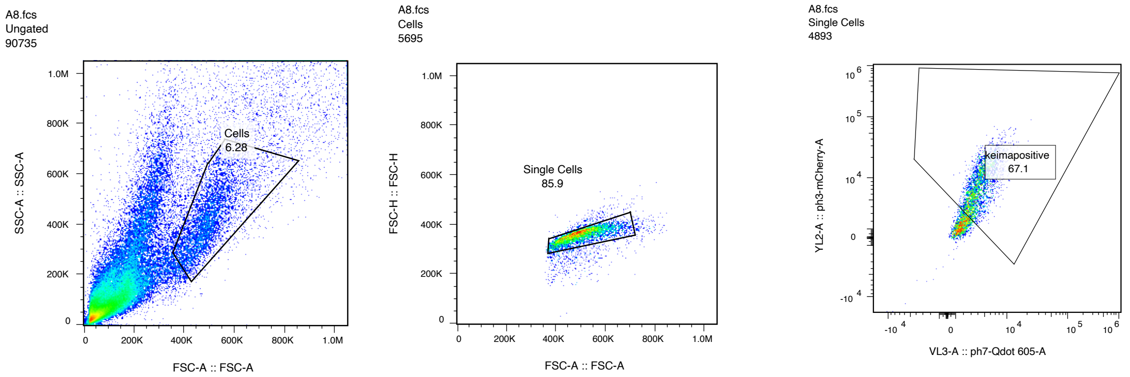


Cells spiked with Propidium iodine to test viability gating example:

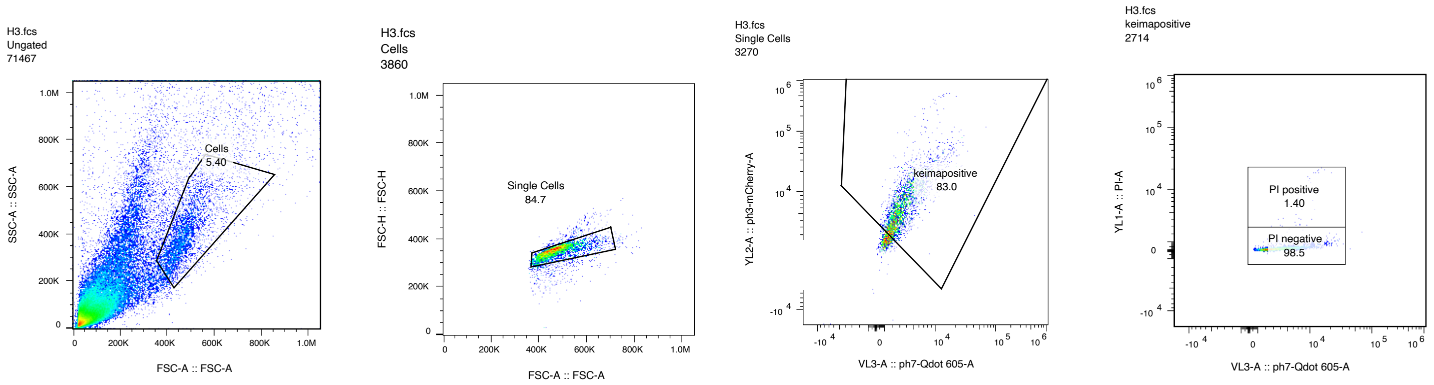


Extended
Figure
4b

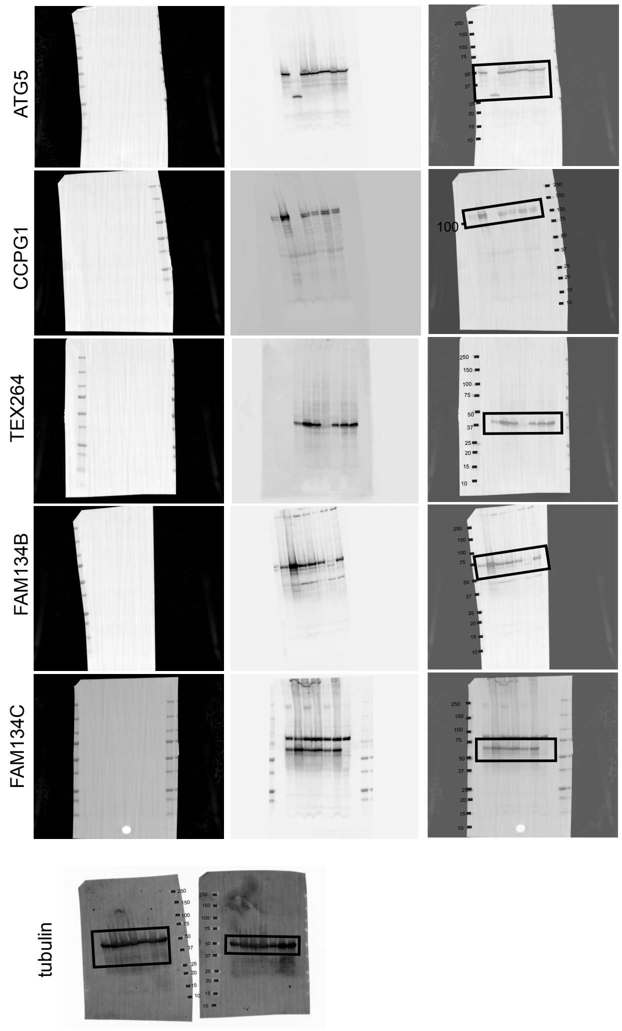


Extended
Figure
4d

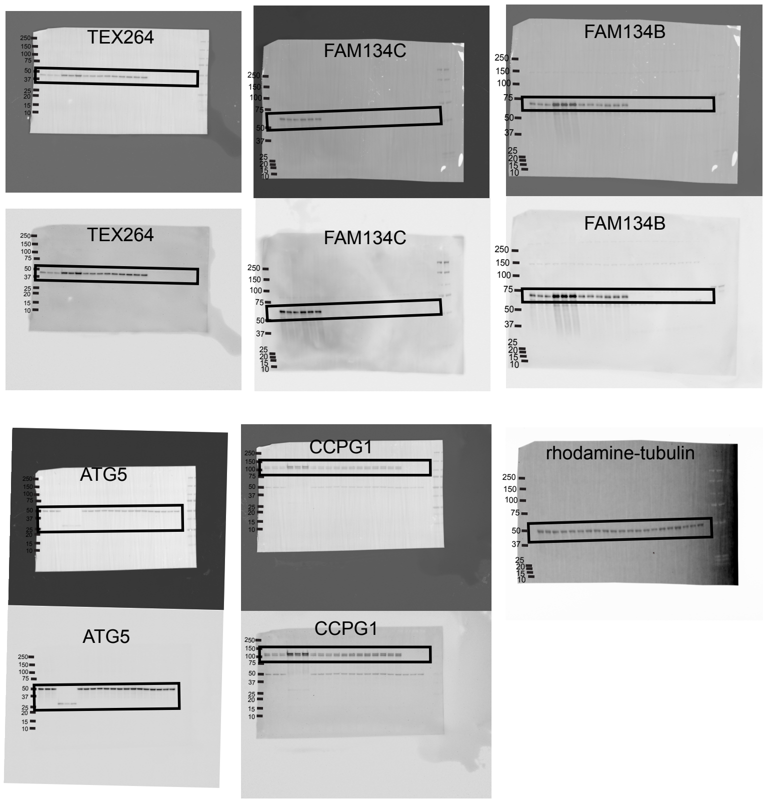

Supplement: Supplementary file 10 — Unprocessed western blots and flow cytometry gating strategy. [file 41556_2024_1356_MOESM10_ESM.pdf]
