# Supplementary material for: Combinatorial selective ER-phagy remodels the ER during neurogenesis
Source: Nat Cell Biol. 2024 Mar 1;26(3):378–92. doi: 10.1038/s41556-024-01356-4 (PMC10940164; doi:10.1038/s41556-024-01356-4)

Extended
Figure
8d

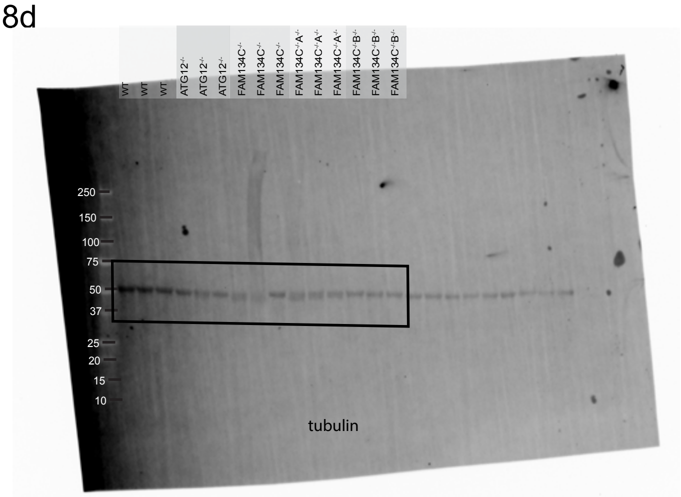

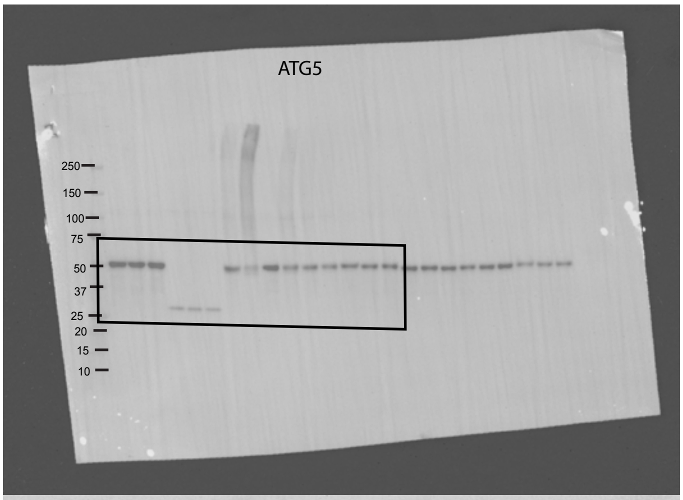

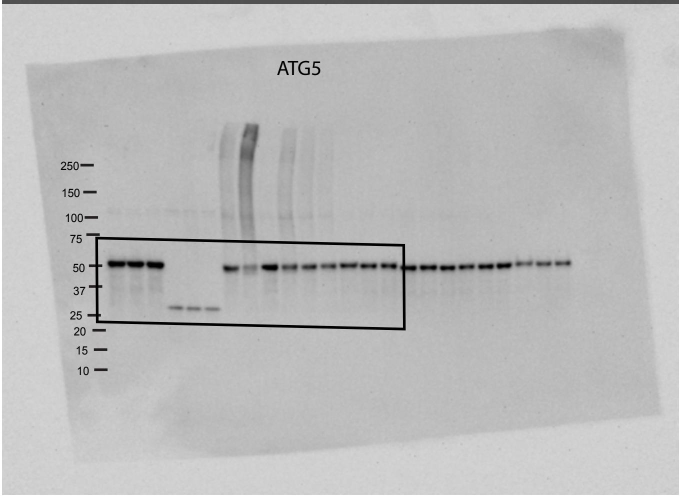

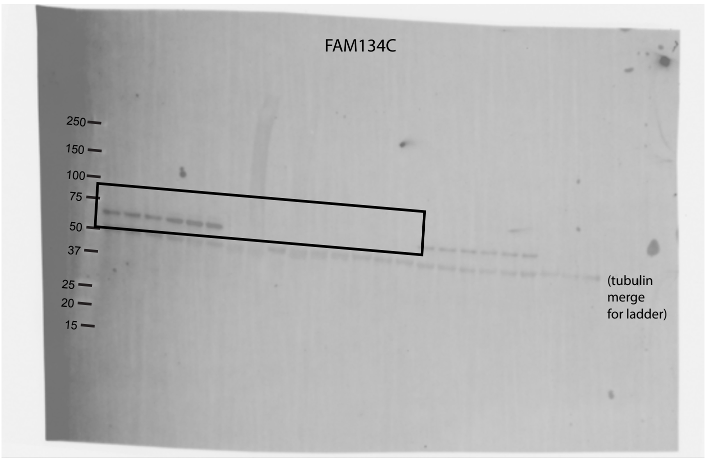

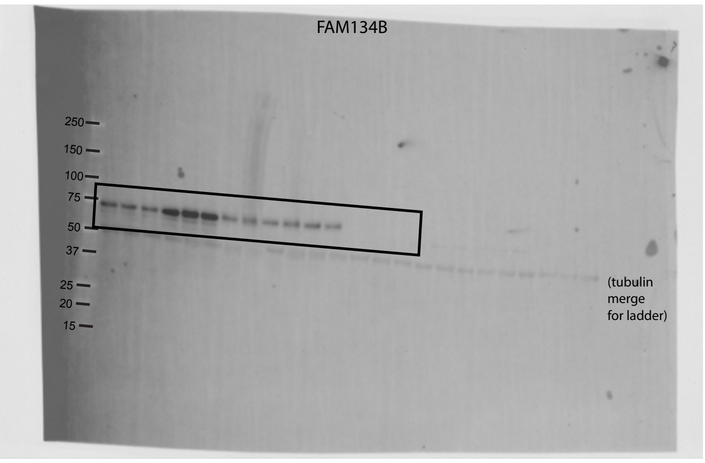

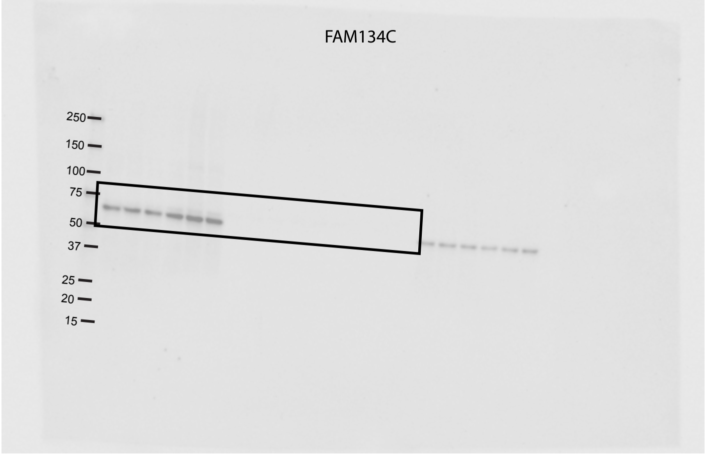

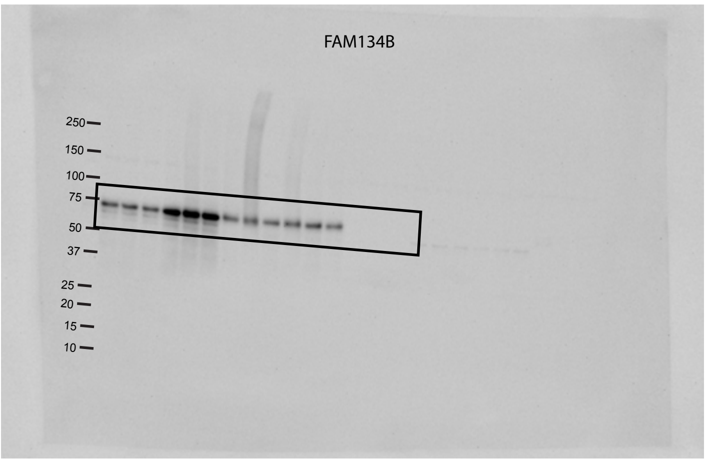

Supplement: Supplementary file 12 — Unprocessed western blots. [file 41556_2024_1356_MOESM12_ESM.pdf]
