# Supplementary material for: Combinatorial selective ER-phagy remodels the ER during neurogenesis
Source: Nat Cell Biol. 2024 Mar 1;26(3):378–92. doi: 10.1038/s41556-024-01356-4 (PMC10940164; doi:10.1038/s41556-024-01356-4)

Hoyer et al source data
Figure 7
Example bar plot for ratio check and corresponding normalization
Other bar plots for ratio check (pre-normalization)

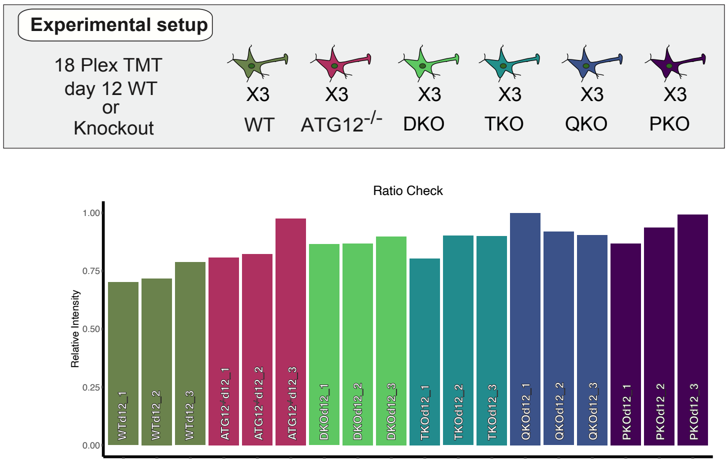

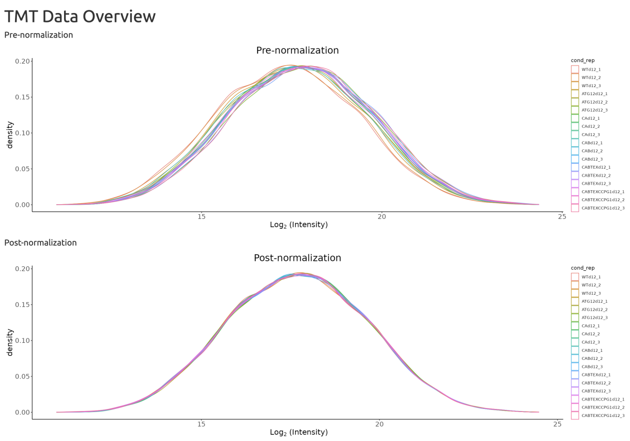

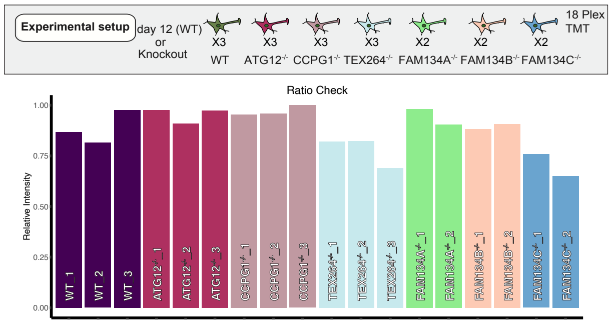

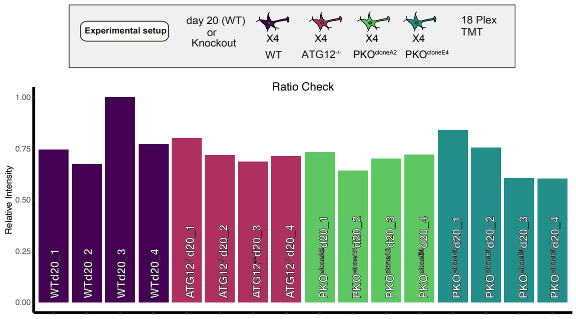

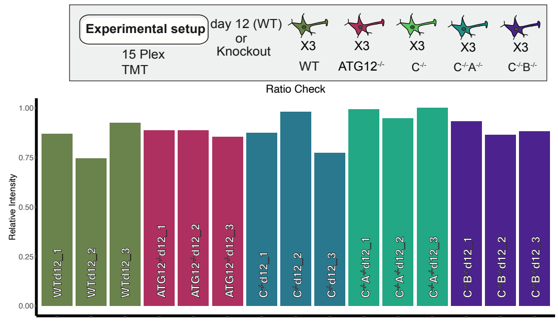

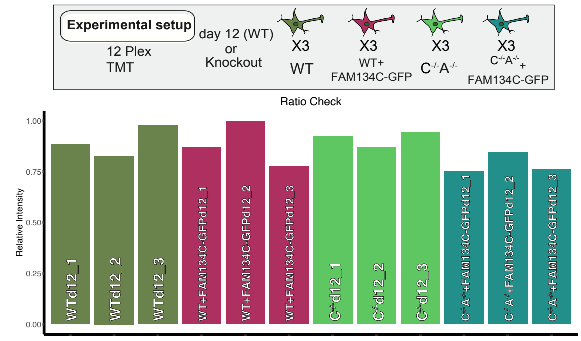

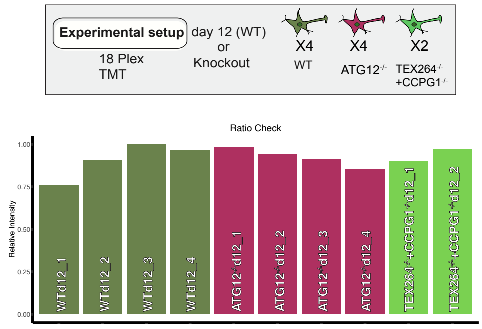

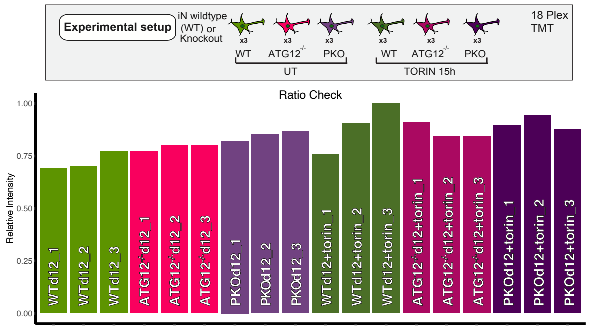

Supplement: Supplementary file 14 — Proteomics barplot ratio check and normalization strategy. [file 41556_2024_1356_MOESM14_ESM.pdf]
